# Acquisition of chess knowledge in AlphaZero

**DOI:** 10.1073/pnas.2206625119

**Published:** 2022-11-14

**Authors:** Thomas McGrath, Andrei Kapishnikov, Nenad Tomašev, Adam Pearce, Martin Wattenberg, Demis Hassabis, Been Kim, Ulrich Paquet, Vladimir Kramnik

**Affiliations:** ^a^DeepMind, London, United Kingdom;; ^b^Google Brain, Mountain View, CA 94043;; ^c^School of Engineering and Applied Sciences, Harvard University, Cambridge, MA 02134;; ^d^Google Research, Mountain View, CA 94043;; ^e^World Chess Champion, 2000–2007

**Keywords:** machine learning, artificial intelligence, interpretability, reinforcement learning, deep learning

## Abstract

We analyze the knowledge acquired by AlphaZero, a neural network engine that learns chess solely by playing against itself yet becomes capable of outperforming human chess players. Although the system trains without access to human games or guidance, it appears to learn concepts analogous to those used by human chess players. We provide two lines of evidence. Linear probes applied to AlphaZero’s internal state enable us to quantify when and where such concepts are represented in the network. We also describe a behavioral analysis of opening play, including qualitative commentary by a former world chess champion.

## Significance

Seventy years ago, Alan Turing conjectured that a chess-playing machine could be built that would self-learn and continuously profit from its own experience. The AlphaZero system—a neural network-powered reinforcement learner—passed this milestone. In this paper, we ask the following questions. How did it do it? What did it learn from its experience, and how did it encode it? Did it learn anything like a human understanding of chess, in spite of having never seen a human game? Remarkably, we find many strong correspondences between human concepts and AlphaZero’s representations that emerge during training, even though none of these concepts were initially present in the network.

Chess has been a testing ground for artificial intelligence since the time of Alan Turing (1). A quarter century ago, the first engines appeared that were able to outplay world champions, most famously DeepBlue (2). Although able to win against people, these engines relied largely on human knowledge of chess as encoded by expert programmers. By contrast, a new generation has appeared of highly successful chess engines that learn to play chess without using any human-crafted heuristics or even seeing a human game. The first such engine, the AlphaZero (3) system, has at its core an artificial neural network that is trained entirely through self-play. AlphaZero reliably won games not just against top human players but also, against the previous generation of chess engines.

The success of an entirely self-taught system raises intriguing questions. What exactly has the system learned? Having developed without human input, will it be inevitably opaque? Can its training history shed light on human progress in chess? A human player naturally picks up basic concepts while playing: that a queen is worth more than a pawn or that a check to the king is important. Can we find qualitative or quantitative evidence of such concepts in AlphaZero’s neural network?

Evidence from other domains suggests that deep learning often does produce correlates of human concepts. Neurons in an image classifier may signal the presence of human concepts in an image (4–6). Certain language models reproduce sentence parse trees (7). Networks may even learn to connect visual and textual versions of the same concept (8). Although these examples are compelling, each of these networks was exposed to human-generated data and (at least in the case of classifiers) human concepts via the choice of classification categories.

In this paper, we investigate how AlphaZero represents chess positions and the relation of those representations to human concepts in chess. Although the results are far from a complete understanding of the AlphaZero system, they show evidence for the existence of a broad set of human-understandable concepts within AlphaZero. We pair these quantitative results with qualitative analysis from a human world chess champion and compare AlphaZero’s choice of opening over its training to human progress in opening analysis. Furthermore, we observe interesting variance between concepts in terms of when they are learned during training, as well as where in the network’s reasoning chain they are learned. Our results suggest that it may be possible to understand a neural network using human-defined chess concepts, even when it learns entirely on its own.

## Our Approach

We take a quantitative and qualitative approach to interpreting AlphaZero. Quantitatively, we apply linear probes to assess whether the network is representing concepts familiar to chess players. Meanwhile, behavioral analysis of AlphaZero presents an obvious difficulty since its game play is so far beyond a typical player. We address this issue by using behavioral analyses from a former world chess champion, V.K.* With his unique perspective, we analyze qualitative aspects of AlphaZero, especially with regard to opening play. Thanks to databases, such as ChessBase, data on human games are plentiful, so we can compare the evolution of AlphaZero’s play during training to the evolution of move choices in top-level human chess. We leverage the existence of a broad range of human chess concepts in conventional chess engines, such as Stockfish, to annotate positions with concept data. We have made a curated set of key positions with both human and AlphaZero play data available online.

## Summary of Results

### Many Human Concepts Can Be Found in the AlphaZero Network.

We demonstrate that the AlphaZero network’s learned representation of the chess board can be used to reconstruct, at least in part, many human chess concepts. We adopt the approach of using concept activation vectors (6) by training sparse linear probes for a wide range of concepts, ranging from components of the evaluation function of Stockfish (9), a state-of-the-art chess engine, to concepts that describe specific board patterns.

### A Detailed Picture of Knowledge Acquisition during Training.

We use a simple concept probing methodology to measure the emergence of relevant information over the course of training and at every layer in the network. This allows us to produce what we refer to as what–when–where plots, which detail what concept is learned, when in training time it is learned, and where in the network it is computed. What–when–where plots are plots of concept regression accuracy across training time and network depth. We provide a detailed analysis for the special case of concepts related to material evaluation, which are central to chess play.

### Comparison with Historical Human Play.

We compare the evolution of AlphaZero play and human play by comparing AlphaZero training with human history and across multiple training runs, respectively. Our analysis shows that despite some similarities, AlphaZero does not precisely recapitulate human history. Not only does the machine initially try different openings from humans, it plays a greater diversity of moves as well. We also present a qualitative assessment of differences in play style over the course of training.

## Past and Related Work

Here, we review prior work on neural network interpretability and the role of chess as a proving ground for Artificial Intelligence (AI).

### Concept-Based Explanations.

Neural network interpretability is a research area that encompasses a wide range of approaches and challenges (10–12). Our interest in relating network activations to human concepts means that we focus primarily on so-called concept-based explanations.

Concept-based methods use human understandable concepts to explain neural network decisions (6, 13–16) instead of using input features (e.g., pixels) (17–19). Concept-based explanations have been successfully used in complex scientific and medical domains, as seen in refs. 20–27 and in concurrent work on an agent trained to play the board game Hex (28), as well as in studying Go-playing agents using concepts derived from natural language annotations (29). Similar to many input feature-based methods, concept methods also only represent correlation and not causation. While there exist causal concept-based methods (30, 31), this work focuses on analysis without having to assume a causal graph that could incorrectly represent the internal mechanism.

A key step for these methods is to operationalize human-understandable concepts. One common approach, for example, is to define a concept by a set of exemplars (6). In the case of chess, however, we do not need to start from scratch; many standard concepts (e.g., king safety or material advantage) are already expressed in simplified, easy-to-compute functions inside modern chess engines.

### Interpretability in Chess.

Progress in chess AI has followed the advent of modern computing, ranging from advances in symbolic AI to recent self-learned neural network engines, like AlphaZero (3) and Lc0 (32). Methods to interpret a chess engine’s decisions can be categorized depending on ingredients (medium) and the purpose of explanations. For example, tree structure explanations for teaching purposes (33) and saliency-based methods (i.e., highlighting the pieces of highest importance for playing a selected move) for understanding a trained model’s behavior (34) have been explored. Another medium explored is using natural language to generate commentary using data from social media (35), to craft a question-answering dataset for chess (36), or to learn evaluation functions (37) that reflect sentiment in chess discussion.

AI has also been used to analyze chess itself by modeling differences between the play style (38) or strength (39) of different human players as well as machine performance on chess variants (40–42). Our work uses an additional ingredient—concepts—to analyze what AlphaZero has learned.

## AlphaZero: Network Structure and Training

### Network Outputs.

This paper investigates the development of chess knowledge within the AlphaZero neural network. We first provide context for our analysis by briefly describing this system. AlphaZero (3) has two components. Its neural network

**Fig. 1.**
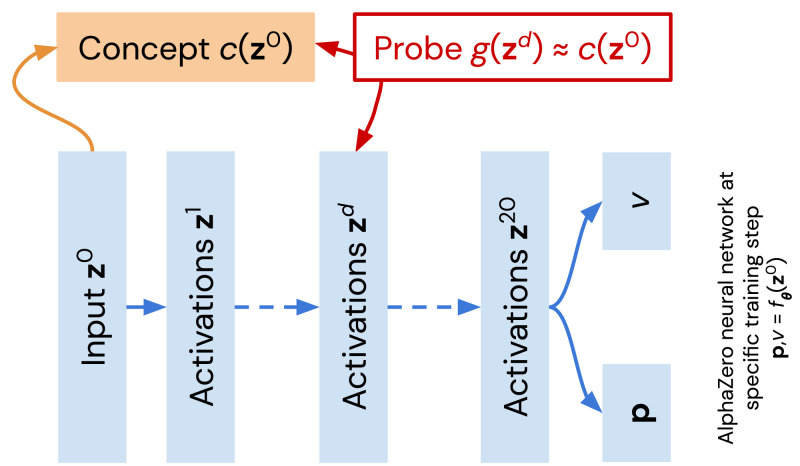
Probing for human-encoded chess concepts in the AlphaZero network (shown in blue). A probe—a generalized linear function $g(z^{d})$—is trained to approximate $c(z^{0})$, a human-interpretable concept of chess position $z^{0}$. The quality of approximation $g(z^{d})\approx c(z^{0})$, when averaged over a test set, gives an indication of how well a layer (linearly) encodes a concept. For a given concept, the process is repeated for the sequence of networks that are produced during training for all the layers in each network.

[1] $$p,v=f_{\theta }(z^{0})$$

computes a probability distribution **p** for a next move and the expected outcome *v* of the game from a state $z^{0}$. Its Monte Carlo tree search (MCTS) component uses the neural network to repeatedly evaluate states and update its action selection rule. A “state” consists of a current chess board position and a history of preceding positions along with ancillary information, such as castling rights, and it is represented as a real-valued vector $z^{0}$. The outputs **p** and *v* are computed by the “policy head” and the “value head” in the AlphaZero network in Fig. 1. The output **p** is typically referred to as AlphaZero’s “prior,” as it is a distribution over moves that is updated by the MCTS procedure. In this work, we only investigate the neural network component of AlphaZero, so we use the prior **p** directly rather than the move distribution following MCTS. This is not necessarily the move that AlphaZero will play when search is enabled, but training does update **p** toward the distribution after MCTS has been applied (*SI Appendix*, section 1 has details).

### AlphaZero Network Architecture.

The AlphaZero network in Fig. 1 contains a residual network (ResNet) backbone (43), also known as the torso, which is followed by separate policy and value heads. ResNets are made up of a series of layers connected by a network block and an identity connection (also known as a skip connection). This gives the activations of a ResNet the following simple structure:

[2] $$z^{d+1}=z^{d}+f_{\theta }^{d}(z^{d}),$$

where $z^{d}$ are the activations at depth d=1,…,D=20; $f_{\theta }^{d}$ is the function implemented by the *d*th residual block; and $z^{0}$ is the input. In the AlphaZero network, all residual blocks are two-layer rectified convolutions. The value head comprises a 1 × 1 rectified convolution followed by a tanh-activated linear layer, and the policy head comprises a 1 × 1 rectified convolution followed by a second 1 × 1 convolution and a softmax layer. Both heads are functions of $z^{20}$. The encoding of the input and details regarding the network are described in refs. 3 and 44 as well as *SI Appendix*, section 1.

### AlphaZero Training Iterations.

Starting with a neural network with randomly initialized parameters θ, the AlphaZero network is trained from data that are generated as the system repeatedly plays against itself. Our experimental setup updates the parameters θ of the AlphaZero network over 1 million gradient descent training steps, an arbitrary training time slightly longer than that of AlphaZero in ref. 3. Training data for each gradient step consist of input positions with their MCTS move probability vectors and final game outcomes. The network is trained to predict the MCTS move probabilities and game outcome in **p** and *v*. Positions and their associated data are sampled from the self-play buffer containing the previous 1 million positions. At most, 30 positions are sampled from a game. Stochastic gradient descent steps are taken with a batch size of 4,096 in training. After every 1,000 (1k; for brevity, the notation “k” denotes “thousand”; e.g., 32k is 32,000) training steps, the networks that are used to generate self-play games are refreshed, meaning that training data in the buffer are frequently generated by a different network than the network being updated.

## Encoding of Human Conceptual Knowledge

From random initialization, AlphaZero’s neural network learns a sophisticated evaluation of a position through many rounds of progressive improvement via self-play. One of our main contributions is to map this evolution in network parameters to changes in human-understandable concepts. We do this through a simple linear probing methodology described here.

### Human-Coded Concepts.

We adopt a simple definition of concepts as user-defined functions from network input $z^{0}$ to the real line, shown in orange in Fig. 1. A simple example concept could detect whether the playing side has a bishop (♗) pair (i.e., both a dark-squared bishop and a light-squared bishop):

[3] $$c(z^{0})=\{\begin{array}{ll} 1 & \;\text{if}\;z^{0}\;\text{has}\;\text{a}♗\;\text{pair}\;\text{for}\;\text{the}\;\text{playing}\;\text{side}\\ 0 & \;\text{otherwise}. \end{array}$$

Most concepts are more intricate than merely looking for bishop pairs and can take a range of integer values (for instance, the difference in the number of pawns) or continuous values (such as the total score as measured by the Stockfish 8 chess engine). An example of a more intricate concept is mobility, where a chess engine designer can write a function that gives a score for how mobile pieces are in $z^{0}$ for the playing side compared with that of the opposing side. For our purposes, concepts are prespecified functions that encapsulate a particular piece of domain-specific knowledge.

We use components of Stockfish 8’s position evaluation function as concepts (9), along with our own implementations of 116 concepts (*SI Appendix*, Tables S1–S3). The use of Stockfish 8 is intentional, as it allows for insights from this paper to refer to observations in ref. 45.

### Concepts and Activations Dataset.

We randomly selected 10^5^ games from the full ChessBase archive (46) and computed concept values and AlphaZero activations for every position in this set. Any duplicate positions in this set were removed using the positions’ Forsyth–Edwards notation (FEN) strings. We then randomly sampled training, validation, and test sets from the deduplicated data. Continuous-valued concepts used training sets of 10^5^ unique positions (not games), with validation and test sets consisting of a further $3\times 10^{4}$ unique positions each. Binary-valued concepts were balanced to give equal numbers of positive and negative examples, which restricted the data available for training as some concepts only occur rarely. The minimum training dataset size for any binary concept was 50,363, and the maximum size was 10^5^.

### Probing for Human Concepts.

We search for human concepts at scale by using a simple linear probing methodology; for a given concept *c*, we train a sparse regression probe *g* from the network’s activations at depth *d* to predict the value of *c*. The training set consists of 10^5^ naturally occurring chess positions from the ChessBase dataset, following Fig. 1. The score (test accuracy) we report is measured in a separate test set. By comparing the scores from different concept probes both for networks at different training steps in AlphaZero’s self-learning cycle as well as for different layers in each network, we can extract when and where the concept is learned in the network. Let notation $z^{d}=f^{1:d}(z^{0})$ refer to the *d*th residual block’s activations $z^{d}$ as a function of $z^{0}$. More formally, given $f^{1:d}(z^{0})$ and a concept $c(z^{0})$, we train a probe $g_{w}(z^{d})$ to minimize a loss function L,

[4] $$w^{*}=\arg\underset{{}_{w}}{\min}\;L\left(g_{w}\left(f^{1:d}(z^{0})\right),\;c(z^{0})\right)+\lambda |w|,$$

as illustrated in Fig. 1. For brevity, we drop the dependence on the “what” (the name of specific concept *c*), the “when” (the network’s specific training step *t* in AlphaZero’s self-learning cycle), and the “where” (the layer index *d*) when denoting **w**, but we train a new set of regression weights in every case. Scalar λ≥0 is an *L*_1_ regularizer that is chosen by cross-validation. For binary-valued concepts (for instance, whether the current player is in check), we use logistic regression, and for all other concepts, we use linear regression:

[5] $$g_{w}(z^{d})=w^{T}[z^{d},1]\;(\text{continuous}\;\text{concepts})$$

[6] $$g_{w}(z^{d})=\sigma \left(w^{T}[z^{d},1]\right)\;(\text{binary}\;\text{concepts}).$$

During probe training, we train only the probe parameters **w**, while the neural network parameters θ are left unchanged. We train using the squared error loss $L(x,y)={\left(x-y\right)}^{2}$. Probes are evaluated on a test set of 30k positions from the ChessBase dataset. During evaluation, we score binary-valued probes using the fraction of correct class predictions (normalized so that 50% accuracy gives a score of zero), and all other probes are scored using the coefficient of determination *r*^2^.

### Tracking Concept Emergence across Training Time and Network Depth.

**Fig. 2.**
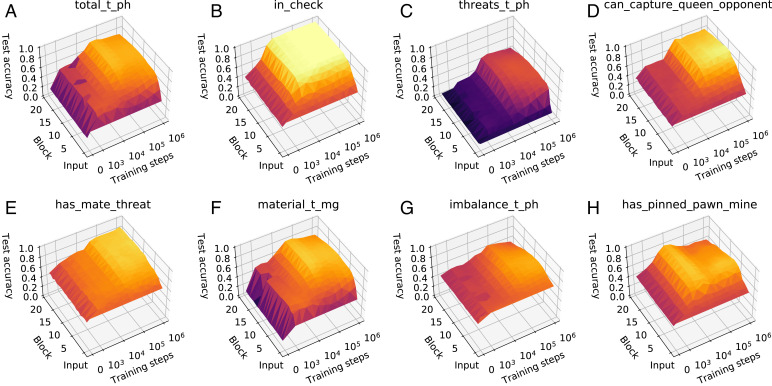
What–when–where plots for a selection of Stockfish 8 and custom concepts. Following Fig. 1, we count a ResNet “block” as a layer. (*A*) Stockfish 8’s evaluation of total score. (*B*) Is the playing side in check? (*C*) Stockfish 8’s evaluation of threats. (*D*) Can the playing side capture the opponent’s queen? (*E*) Could the opposing side checkmate the playing side in one move? (*F*) Stockfish 8’s evaluation of “material score.” (*G*) Stockfish 8’s material score. Past 10^5^ training steps this becomes less predictable from AlphaZero’s later layers. (*H*) Does the playing side have a pawn that is pinned to the king?.

For a given concept, we can train and evaluate a separate probe for every network depth *d*. Plotting the change in test set scores of a concept probe against network depth generates a profile of where (if anywhere) the concept is being computed. Comparing across multiple training steps *t* allows us to track the evolution of these profiles. We combine these regression scores into a single plot per concept that we refer to as what–when–where plots because they visualize what concept is being computed, where in the network this computation happens, and when during network training this concept emerged. What–when–where plots for a selection of concepts are given in Fig. 2. Notably, Stockfish 8’s threats function and AlphaZero’s representation of thereof, as detectable by a linear probe, become more and finally, less correlated as AlphaZero becomes stronger (Fig. 2*C*). By using a wide range of concepts, we can build up a detailed picture of the emergence of human concepts over the course of AlphaZero’s training. What–when–where plots for the full set of concepts are given in *SI Appendix*, Figs. S2–S13, while *SI Appendix*, Figs. S21–S32 show plots for AlphaZero trained from a different seed.

### Interpreting What–When–Where Plots.

What–when–where plots naturally incorporate two baselines needed for comparisons in probing methodologies (47): regression from input, which is shown at layer zero, and regression from activations of a network with random weights, which is shown at training step 0. These allow us to conclude that changes in regression accuracy are solely due to changes in the network’s representations. We show a third baseline, regression to random labels, in *SI Appendix*, Fig. S7, which is zero everywhere.

There are formal information-theoretic answers to the question of what an increase in *r*^2^ means. Predictive V-information (48) quantifies the idea that although deterministic functions cannot create mutual information, they can render it more accessible to a computationally bounded observer. When the observer is restricted to using linear functions, predictive V-information is proportional to the *r*^2^ of the best such function. What–when–where plots can hence be interpreted as measuring increases in predictive V-information. In our case, the network’s computations $f^{1:d}(z^{0})$ are making more information about $c(z^{0})$ accessible to an observer restricted to using a linear model.

### Consistent Patterns in Concept Development.

Many of these what–when–where plots show a consistent pattern:low regression accuracy at network initialization (*t* = 0) and from the network input (*d* = 0)—showing that the what–when–where plots are measuring concept-relevant changes in computation rather than the concept being predictable from the input or due to its projection into a higher-dimensional space. In many cases, regression accuracy remains low throughout the network until around 32k steps, demonstrating a key point in the development of AlphaZero’s representations. After this point, regression accuracy increases rapidly with network depth before plateauing and remaining constant through later layers (typically past *d* = 10). This indicates that all concept-relevant computations have occurred relatively early in the network and that later residual blocks are either performing move selection or computing features outside our concept set.

### Insights from Structure in Regression Errors: Look ahead.

**Fig. 3.**
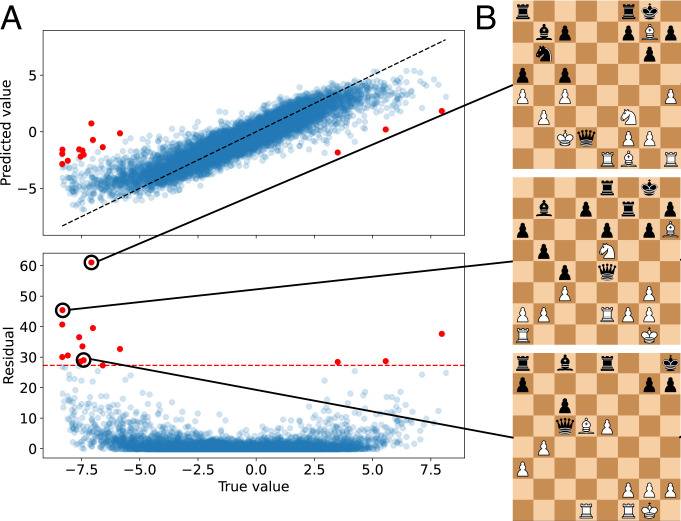
Evidence of patterns in regression residuals. (*A*, *Upper*) True and predicted values for Stockfish 8 score total_t_ph on a test set for the probe at depth 10 for the network after a million training steps. The dashed line indicates a perfect fit. The red markers indicate predictions in the 99.95th percentile of residuals. (*A*, *Lower*) True value and residual for Stockfish 8 score as in *A*, *Upper*. The red dashed line indicates the 99.95th percentile cutoff. (*B*) High-residual positions corresponding to the data points marked in *A*. Note that black’s queen can be taken in all positions. This is true for all 12 high-residual pieces where the regressed score is more favorable to white than the Stockfish score.

In this section, we explore a potential semantic meaning in error outliers for the total_t_ph (Stockfish 8 “total score”) concept. To do this, we computed the Stockfish 8 score concept $c_{\mathrm{total}}(z^{0})$ for each position $z^{0}$ in the test set as well as $g_{w}^{\mathrm{total}}(z^{0})$ from Eq. **5** for the probe’s highest accuracy point (*d* = 10 and after a million training steps). In Fig. 3, we show the highest residuals ${\left(c_{\mathrm{total}}(z^{0})-g_{w}^{\mathrm{total}}(z^{0})\right)}^{2}$ and the positions corresponding to the residuals in the 99.95th percentile, representing the positions with the most extreme prediction errors.

The outliers highlight potential differences between AlphaZero’s search and Stockfish’s concept calculation. In all 12 outlier positions where the regressed score is more favorable to white than the Stockfish score, black’s queen can be recaptured as part of an exchange sequence. We hypothesize that this is due to AlphaZero network’s value head and Stockfish 8’s evaluation function taking on fundamentally different roles, purely because of the way search differs between the engines. AlphaZero’s MCTS simulations run to fixed depth. The AlphaZero network, therefore, has to encode some minimal form of look ahead; it seems to be required when an exchange sequence is simulated only halfway before the maximum MCTS simulation depth is reached. This is similar to the way that finite time horizons lead learned reward functions to encode search information (49). Stockfish, on the other hand, dynamically increases evaluation depth during exchange sequences. There is information that comes from looking ahead that Stockfish’s evaluation function does not have to encode simply because there is other code that ensures that such information is incorporated in the final evaluation.

Additional evidence of look ahead is found in Fig. 2*E*. The concept measuring the existence of a mate threat involves looking ahead one move and demonstrates that some one-step planning is arguably encoded. In Fig. 2*E*, accuracy for has_mate_threat rises throughout the network, suggesting that further processing of threats occurs in later layers and that some (important) direct consequences of its opponent’s potential moves are represented.

### Relating Concepts to AlphaZero’s Position Evaluation.

Many human-defined concepts can be predicted with high but not perfect accuracy from intermediate representations in AlphaZero when training has progressed sufficiently, but this does not determine how these concepts relate to the outputs of the network. In this section, we investigate the relation of piece count differences and high-level Stockfish concepts to AlphaZero’s value predictions. Given a vector of human-encoded concepts $c(z^{0})=[c_{1}(z^{0}),c_{2}(z^{0}),\ldots ,c_{N}(z^{0})]$, we train a generalized linear model

**Fig. 4.**
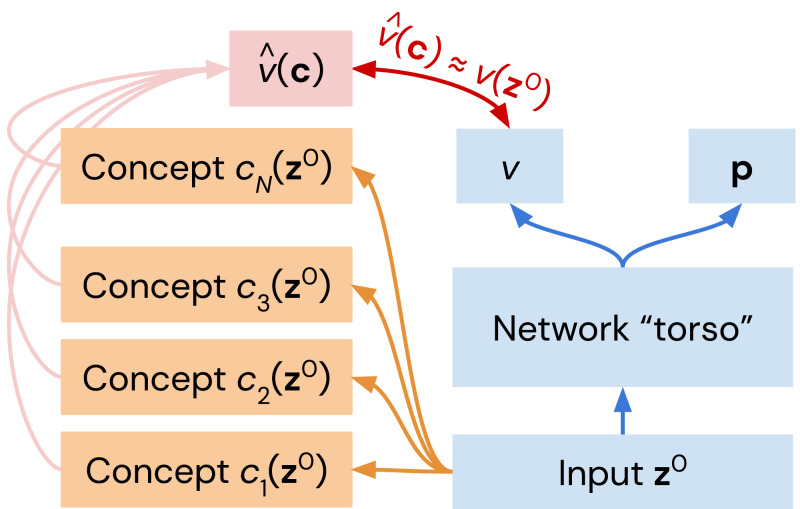
Value regression methodology. We train a generalized linear model on concepts to predict AlphaZero’s value head for each neural network checkpoint.

[7] $${\hat{v}}_{w}(z^{0})=\tanh(w^{T}[c(z^{0}),1])$$

to predict the output of the AlphaZero network value head $v(z^{0})$. This approach is schematically shown in Fig. 4. The use of tanh nonlinearity is because $v(z^{0})\in (-1,1)$, and tanh also corresponds to the final nonlinear function in the value head. The weights are found by minimizing the *L*_1_ loss between ${\hat{v}}_{w}(z^{0})$ and the neural network output $v(z^{0})$ over 10^5^ training positions, deduplicated in the same way as the positions used for concept regression.

### Piece Value.

**Fig. 5.**
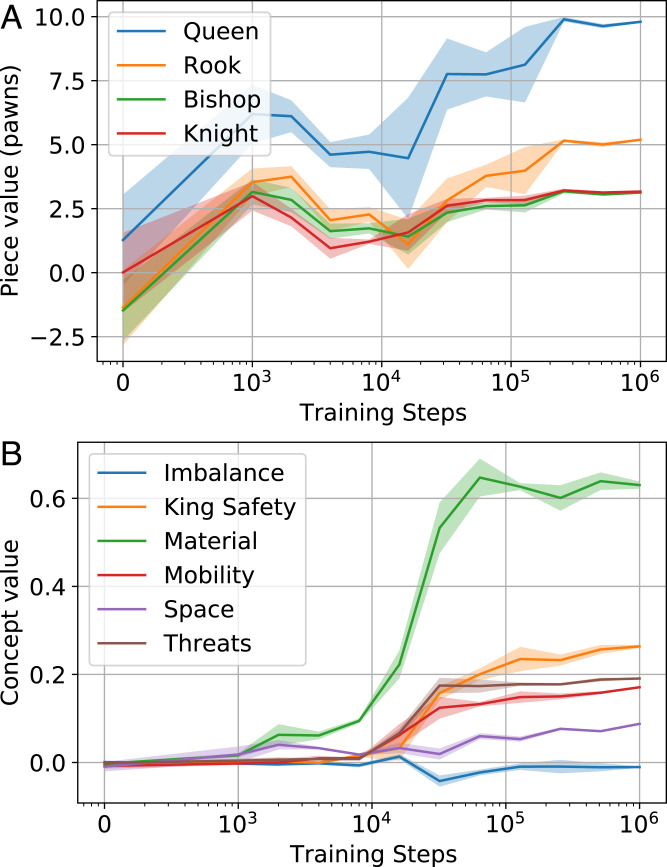
Value regression from human-defined concepts over time. (*A*) Piece value weights converge to values close to those used in conventional analysis. Error bars show 95% CIs of the mean across three seeds, with three samples per seed. (*B*) Material predicts value early in training, with more subtle concepts, such as mobility and king safety, emerging later. Error bars show 95% CIs of the mean across three seeds, with three samples per seed.

Simple values for material are one of the first things a beginner chess player learns, and they allow for basic assessment of a position. We began our investigation of the value function by using only the piece count difference as the “concept vector.” In this case, similar to ref. 41, the vector $c(z^{0})=[d_{♙}(z^{0}),d_{♘}(z^{0}),d_{♗}(z^{0}),d_{♖}(z^{0}),d_{♛}(z^{0})]$ contains the difference in numbers of pawns $d_{♙}$, knights $d_{♘}$, bishops $d_{♗}$, rooks $d_{♖}$, and queens $d_{♛}$ between the current player and the opponent. For piece value regression, we use only positions where at least one piece count differs between white and black. The evolution of piece weights **w**, normalized by the corresponding pawn weight, is shown in Fig. 5*A*. The figure shows that piece values converge toward commonly accepted values after 128k training steps.

### Higher-Level Concepts.

We explore the relative contributions of the high-level Stockfish concepts imbalance, king_safety, material, mobility, space, and threats (*SI Appendix*, Table S1) to predicting *v*. We normalized each of the high-level Stockfish concepts by dividing by their respective SDs and used the vector $c(z^{0})\text{​}=\text{​}[\text{​}c_{\mathrm{imb}}(z^{0}),c_{\mathrm{king}\_\mathrm{sf}}(z^{0}),c_{\mathrm{mat}}(z^{0}),$ $c_{\mathrm{mob}}(z^{0}),c_{\mathrm{space}}(z^{0}\text{​}),c_{\mathrm{threats}}(z^{0})]$ with the concepts’ “t_ph” function calls^†^ in the generalized linear model of Eq. **7**. Fig. 5*B* illustrates the progression of the components of **w** from Eq. **7** over training steps *t*.

At initialization, no concept weights are statistically significantly different from zero at 95% confidence. Material and space are the first concepts to differ significantly from zero at 2k training steps. More sophisticated concepts, such as king safety, threats, and mobility, become statistically significantly nonzero only past 8k steps and only increase substantially at 32k steps and beyond. This coincides with the point when there is a sharp rise in *r*^2^ in their what–when–where plots in Fig. 2 and *SI Appendix*, Figs. S2–S6. The imbalance concept appears to play a marginal role in position evaluation; although it receives nonzero weight at 32k training steps, the weight assigned to it is minimal and rapidly reverts to being approximately zero. Interestingly, although imbalance-related information can be regressed from early layers of the network (Fig. 2*G*), it cannot be regressed from the final layer of the torso, which is consistent with it not playing a direct role in position evaluation.

### Discovering Other Concepts.

As a first step toward unsupervised concept discovery, we use nonnegative matrix factorization (NMF) (50) to decompose AlphaZero’s activation space, as has been done in vision (5) and Reinforcement Learning (RL) models (51). We share the full results as an interactive visualization showing all NMF factors for a range of inputs online, and more details are in *SI Appendix*. Note that most of the factors identified by NMF in later layers remain unexplained; developing methods to explain these factors is an important avenue for future work. A striking feature of the majority of the what–when–where plots is the rapid increase in regression accuracy early in the network followed by a later plateau or decrease. This indicates that our concept set is only probing earlier layers of the network, and to understand later layers, we will need new techniques for concept discovery.

## The Evolution of Opening Play

The opening moves of a human chess game depend on the players’ understanding of strategic and tactical concepts. Choosing an opening depends on the value that the players implicitly place on concepts, like space, material, king safety, or mobility. Historically, advances in human understanding in chess have been reflected in changes in opening style. By analogy, investigating AlphaZero’s preferences in the opening stage of the game may provide a useful window on its progress as it trains. Such an investigation also allows us to shed light on whether AlphaZero in any way recapitulates the history of human chess or wŁhether it develops its understanding of the game in a radically different path. Is there a single “natural” way of acquiring chess concepts?

### The First Move.

**Fig. 6.**
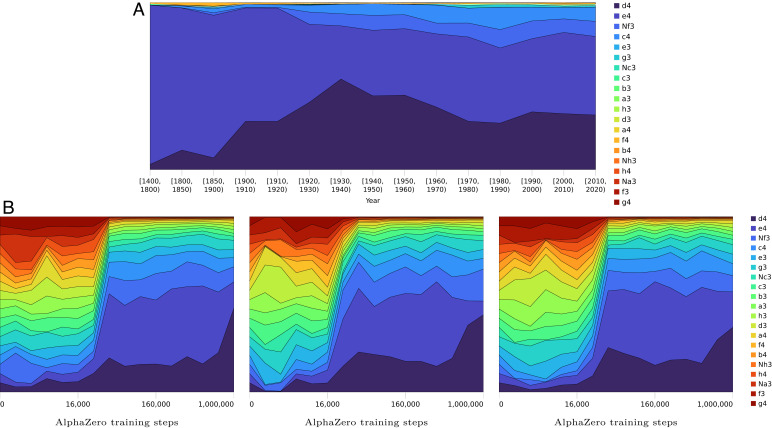
A comparison between AlphaZero’s and human first move preferences over training steps and time. (*A*) The evolution of the first move preference for white over the course of human history, spanning back to the earliest recorded games of modern chess in the ChessBase database. The early popularity of 1. e4 gives way to a more balanced exploration of different opening systems and an increasing adoption of more flexible systems in modern times. (*B*) The AlphaZero policy head’s preferences of opening move as a function of training steps. Training steps are shown on a logarithmic scale. Here, AlphaZero was trained three times from three different random seeds. AlphaZero’s opening evolution starts by weighing all moves equally, no matter how bad, and then, narrows down options. It stands in contrast with the progression of human knowledge, which gradually expanded from 1. e4. The AlphaZero prior swings to a marked preference for 1. d4 in the later stages of training. This preference should not be overinterpreted, as self-play training is based on quick games enriched with stochasticity for boosting exploration.

An analysis of AlphaZero’s prior shows that it starts with an effectively uniform distribution of opening moves, allowing it to explore all options equally, and then remove narrows down options over time (Fig. 6*B*). In quantitative terms, the entropy of AlphaZero’s first move prior reduces from 4.32 bits per move at the beginning of training to an average of 2.86 bits per move after 1 million training steps, averaged across different seeds. By comparison, recorded human games over the last five centuries^‡^ point to an opposite pattern: an initial overwhelming preference for 1. e4, with an expansion of plausible options over time (Fig. 6*A*). In modern tournament play, 1. d4 became slightly more popular in the early twentieth century, along with an increasing popularity of less forcing systems, like 1. c4 and 1. ♘f3. Human first move preferences in the early recorded games between the years 1400 and 1800 empirically encode a mere 0.33 bits of information, increasing to 1.87 bits of information in top-level play at the end of the twentieth century.

In other words, we see two different paths to mastery. Over time, AlphaZero narrowed down options; humans seem to have increased them. The reason for this difference is unclear. It could reflect fundamental differences between people and neural networks. Another possible factor may be that our historical data on human games emphasize collective knowledge of relatively expert players, while AlphaZero’s data include its “beginner-level” games and a single evolving strategy. In the remainder of this section, we study opening move strategy in more detail to refine our understanding.

### Preferences of Main Lines across AlphaZero Versions.

One way to further compare AlphaZero and human play is to focus on opening lines that people currently consider equivalent. When AlphaZero is trained more than once, do the resulting networks show a stable preference for certain lines? We have found many cases where its preferences are not stable over different training runs. We describe one such example in detail, a very important theoretical battleground in top-level human play.

Table 1 gives an overview of AlphaZero’s prior preference for early choices for black in the Ruy Lopez opening following its characteristic sequence of moves 1. e4 e5 2. ♘f3 ♘c6 3. ♗b5. Two responses, 3 … ♘f6 and 3 … a6, are considered equally good by many today, with a rich history behind this assessment. The Berlin defense with 3 … ♘f6 was first championed by GM V.K. at top-level play and has since become a highly fashionable modern equalizing attempt. For many years, it was considered a slightly worse response than 3 … a6, and human chess opening theory has relatively recently appreciated the benefits of the Berlin defense and established effective ways of playing with black in this position.

**Table 1.** The AlphaZero prior network preferences after 1. e4 e5 2. ♘f3 ♘c6 3. ♗b5 for four different training runs of the system

	Seed (left), %	Seed (center), %	Seed (right), %	Seed (additional), %
3 … ♘f6	5.5	92.8	88.9	7.7
3 … a6	89.2	2.0	4.6	85.8
3 … ♗c5	0.7	0.8	1.3	1.3

The prior is given after 1 million training steps, and the seeds (left, center, right) correspond to those in Fig. 6*B*.

**Fig. 7.**
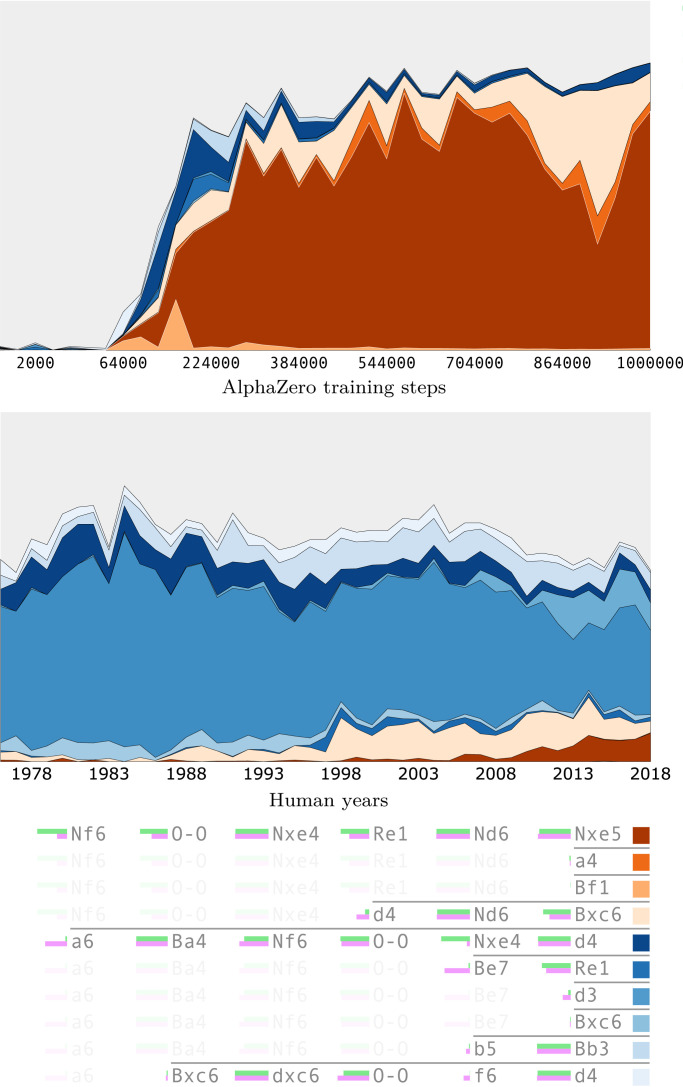
The top four six-ply continuations from AlphaZero’s prior after 1. e4 e5 2. ♘f3 ♘c6 3. ♗b5 in shades of red and the top six six-ply human grandmaster continuations in shades of blue. Assuming 25 moves per ply, there are around a quarter of a billion (25^6^) such six-ply lines of play. *Top* and *Middle* show the relative contribution of these 10 lines in AlphaZero’s prior as training progresses and their frequency in 40 y of human grandmaster play. The AlphaZero training run rapidly adopts the Berlin defense (3 … ♘f6) after around 100k training steps (Table 1). The rows of the table in *Bottom* show the 10 lines, with identical plies being faded to show branching points. The light green bars next to the moves compare the prior preference of the fully trained AlphaZero network (green) with grandmaster move frequencies in 2018 (pink).

We can find different training runs in which AlphaZero converges to either of these two main moves (Table 1). Moreover, individual AlphaZero models encode a strong preference for one move over the other, despite their apparent near equivalence. (That different training runs converge on different lines adds weight to the human opinion that neither provides a definitive advantage.) Given the results of the previous section, where AlphaZero showed a greater diversity in opening moves, one might have expected a more uniform distribution of moves. Furthermore, as illustrated in Fig. 7, the preference is developed rapidly and established early on in training. This is further evidence that there are multiple paths to chess mastery—not just between humans and machine but across different training runs of AlphaZero itself.

### Connection to the Rapid Increase of Basic Knowledge.

To link these results on opening lines to the earlier analysis of concept formation, it is natural to compare the plots of opening move preference to the what–when–where diagrams for different concepts. There seems to be a distinct inflection point in various conceptual plots that coincides with dramatic shifts in opening move preferences. We hypothesize that this is the point in time where the network potentially “understands” the right concepts to correctly recognize good opening lines.

In particular, concepts of material and mobility seem directly relevant to opening theory. Evidence from concept and value regression, notably in Fig. 5, indicates that Stockfish’s evaluation subfunction of material is strongly indicative of the AlphaZero network’s value assessment. Fig. 5 suggests that the concept of material and its importance in the evaluation of a position are largely learned between training steps 10k and 30k and then, refined. Furthermore, the concept of piece mobility is progressively incorporated in AlphaZero’s value head in the same period. It seem reasonable that a basic understanding of the material value of pieces should precede a rudimentary understanding that greater piece mobility is advantageous. This early theory is then incorporated in the development of opening preferences between 25k and 60k training steps.

**Fig. 8.**
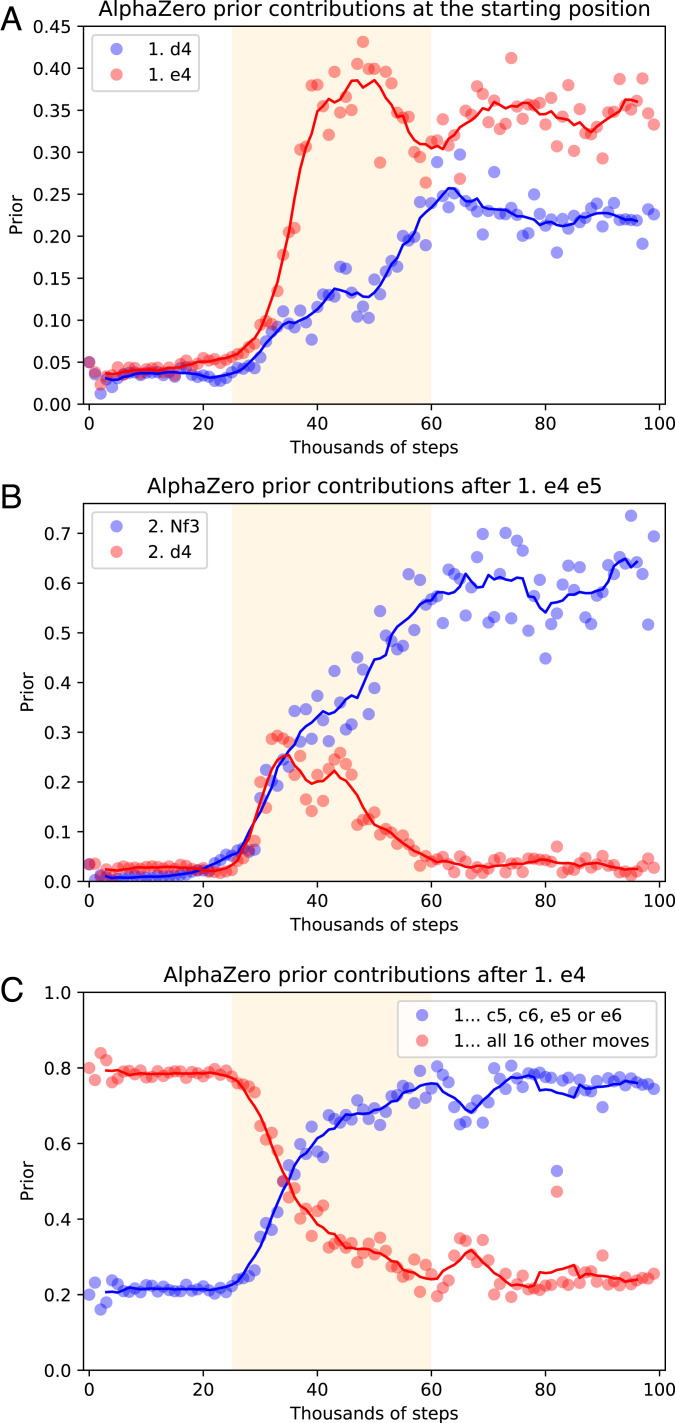
Rapid discovery of basic openings. The randomly initialized AlphaZero network gives a roughly uniform prior over all moves. The distribution stays roughly uniform for the first 25k training iterations, after which popular opening moves quickly gain prominence. In particular, 1. e4 is fully adopted as a sensible move in a window of 10k training steps or in a window of 1% of AlphaZero’s training time. (*A*) After 25k training iterations, e4 and d4 are discovered to be good opening moves and rapidly adopted within a short period of around 30k training steps. (*B*) Rapid discovery of options given 1. e4 e5. Within a short space of time, ♘f3 is settled on as a standard reply, whereas d4 is considered and discarded. (*C*) In the window between 25k and 60k training steps, AlphaZero learns to put 80% of its mass on four replies to e4 and 20% of its mass on all other 16 moves.

To examine the transition, we consider the contribution of classically popular opening moves as a fraction of the AlphaZero prior over all moves (Fig. 8). Fig. 8*A* shows that after 25k training iterations, 1. d4 and 1. e4 are discovered to be good opening moves and are rapidly adopted within a short period of around 30k training steps. Similarly, AlphaZero’s preferred continuation after 1. e4 e5 is determined in the same short temporal window. Fig. 8*B* illustrates how both 2. d4 and 2. ♘f3 are quickly learned as reasonable white moves, but 2. d4 is then dropped almost as quickly in favor of 2. ♘f3 as a standard reply. Looking beyond individual moves, we group AlphaZero’s responses to 1. e4 into two sets: 1 … c5, c6, e5, or e6 and then, all 16 other moves. Fig. 8*C* shows how 1 … c5, c6, e5, or e6 together account for 20% of the prior mass when the network is initialized, as they should, as they are 4 of 20 possible black responses. However, after 25k training steps, their contribution rapidly grows to account for 80% of the prior mass. After the rapid adoption of basic opening moves in the window from 25k to 60k training steps, opening theory is progressively refined through repeatedly updating the training buffer of games with fresh self-play games. While these examples of the rapid discovery of basic openings are not exhaustive, further examples across multiple training seeds are given in *SI Appendix*, Figs. S14–S19.

To sum up, the evidence suggests a natural sequence. First, piece value is discovered; next comes an explosion of basic opening knowledge in a short time window. Finally, the network’s opening theory is refined over hundreds of thousands of training steps. This rapid development of specific elements of network behavior mirrors the recent observation of “phase transition”–like shifts in the inductive ability of large language models (52). In both cases, although overall learning occurs over a long time period, specific foundational capacities occur rapidly in a relatively short period of time.

### Qualitative Evaluation.

To further shed light on the early period in the evolution of chess knowledge within the AlphaZero network, a qualitative assessment is provided by Grand Master V.K., a former world chess champion. This assessment consisted of an analysis of games played between different versions of AlphaZero at different points during its training, prior to having seen other results reported in this paper. In particular, we generated games played between AlphaZero at four distinct points during a single training run: 16k, 32k, 64k, and 128k training steps. The versions at these training steps were played against one another in three different pairings (16k steps vs. 32k steps, 32k vs. 64k steps, and 64k vs. 128k steps), with 100 games generated for each pairing. In this setup, the 32k model wins all 100 games against the 16k; the 64k model wins 79, draws 20, and loses once to the 32k model, and the 128k model wins 52, draws 20, and loses 8 to the 64k model. The assessments were as follows.^§^

16k to 32k.The comparison between 16k and 32k was the simplest. AlphaZero at 16k has a crude understanding of material value and fails to accurately assess material in complex positions. This leads to potentially undesirable exchange sequences and ultimately, losing games on material. On the other hand, AlphaZero at 32k seemed to have a solid grasp on material value, thereby being able to capitalize on the material assessment weakness of 16k.

32k to 64k.The main difference between 32k and 64k, in GM V.K.’s view, is in the understanding of king safety in imbalanced positions. This manifests in the 32k version potentially underestimating the attacks and the long-term material sacrifices of the 64k version as well as the 32k version overestimating its own attacks, resulting in losing positions. Despite the emphasis on king safety, GM V.K. also comments that 64k appears to be somewhat stronger in all aspects than 32k, which is well aligned with our quantitative observations for the changes in the period.

64k to 128k.The theme of king safety in tactical positions resurfaces in the comparisons between 64k and 128k, where it seems that 128k has a much deeper understanding of which attacks will succeed and which would fail, allowing it to sometimes accept material sacrifices made by 64k for purposes of attack yet then, proceed to defend well, keep the material advantage, and ultimately convert to a win. There seems to be less of a difference in terms of positional and endgame play.

These observations echo the analysis of opening lines and value regression; piece value is a keystone concept, developed first. Subsequently, issues around mobility (king safety, attack, and defense) arise. Finally, there is a refinement stage, in which the network learns to make sophisticated trade-offs.

## Conclusions

In this work, we studied the evolution of AlphaZero’s representations and play through a combination of concept probing, behavioral analysis, and examination of AlphaZero’s activations. Examining the evolution of human concepts using probing showed that many human concepts can be accurately regressed from the AlphaZero network after training, even though AlphaZero has never seen a human game of chess, and there is no objective function promoting human-like play or activations. Following on from our concept probing, we reflected on the challenges associated with this methodology and suggested future directions of research. The idea that the neural network encodes human understandable concepts is further supported by the results of the unsupervised NMF method that found multiple human-interpretable factors in AlphaZero’s activations. We have also analyzed the evolution of AlphaZero’s play style through a study of opening theory and an expert qualitative analysis of AlphaZero’s self-play during training. Our analysis suggests that opening knowledge undergoes a period of rapid development around the same time that many human concepts become predictable from network activations, suggesting a critical period of rapid knowledge acquisition. The fact that human concepts can be located even in a system trained by self-play broadens the range of systems in which we should expect to find existing or new human-understandable concepts. We believe that the ability to find human-understandable concepts in the AlphaZero network indicates that a closer examination could reveal more. The next question is as follows. Can we go beyond finding human knowledge and learn something new?

## Supplementary Material

Supplementary File

## Data Availability

Move trees and factorized representation data have been deposited in Google apis (https://storage.googleapis.com/uncertainty-over-space/alphachess/index.html) (53). However, sharing the AlphaZero algorithm code, network weights, or generated representation data would be technically infeasible at present.

## Footnotes

^*^V.K. is a Classical World Chess Champion (2000 to 2006) and an International Chess Federation (FIDE) and Undisputed World Chess Champion (2006 and 2007).

^†^Details are in *SI Appendix*, Tables S1–S3. “t” stands for the “total” side (white–black difference), while “ph” indicates a phase value, a composition that is made up of a middle game “mg,” end game “eg,” and the phase of the game evaluations.

^‡^We base our analysis on a subsample of games from the ChessBase Mega Database (46), which spans from 1475 until the present day.

^§^These qualitative assessments were provided prior to seeing our other quantitative results in order to avoid introducing biases into the analysis.
