# Supplementary material for: Acquisition of chess knowledge in AlphaZero
Source: Proc Natl Acad Sci U S A. 2022 Nov 14;119(47):e2206625119. doi: 10.1073/pnas.2206625119 (PMC9704706; doi:10.1073/pnas.2206625119)
Supplement: Supplementary File [file pnas.2206625119.sapp.pdf]

PNAS
www.pnas.org

Supplementary Information for

Acquisition of Chess Knowledge in AlphaZero

Thomas McGrath, Andrei Kapishnikov, Nenad Tomašev, Adam Pearce, Martin Wattenberg, Demis Hassabis, Been Kim, Ulrich Paquet and Vladimir Kramnik

Corresponding Author name.
E-mail: mcgrathtom@google.com

This PDF file includes:

Supplementary text
Figs. S1 to S79
Tables S1 to S3
SI References

Supporting Information Text

Contents

1 Details of the AlphaZero network architecture and training algorithm 2
A Network architecture . . . 2
A.1 Input encoding . . . 2
A.2 Layers . . . 3
B Network training . . . 5
C Move selection by Monte Carlo Tree Search (MCTS) . . . 5

2 Full concept list 6

3 Regression results for all concepts 9
A Regression results for Stockfish concepts from Table S1 . . . 9
B Regression results for custom concepts from Table S2, excluding capture-related concepts . . . 15
C Regression results for custom concepts from Table S2 related to captures . . . 19
D Regression results for custom pawn-related concepts from Table S3 . . . 20

4 AlphaZero policy progression through training for different training seeds 23

5 Outlier positions in score regression 30

6 Concept regression results for second seed 31
A Regression results for Stockfish concepts from Table S1 (second seed) . . . 31
B Regression results for custom concepts from Table S2, excluding capture-related concepts (second seed) . . . 36
C Regression results for custom concepts from Table S2 related to captures (second seed) . . . 40
D Regression results for custom pawn-related concepts from Table S3 (second seed) . . . 41

7 Exploring activations with unsupervised methods 43
A Non-negative matrix factorisation . . . 43
A.1 Methodology . . . 43
A.2 Results . . . 43

8 Comparison of results across regularisers 45

1. Details of the AlphaZero network architecture and training algorithm

The text below is adapted from our technical report (1).

A. Network architecture. The neural network architecture is illustrated in in Figure S1. The network takes input $\mathbf{z}^0 \in \mathbb{R}^{d_0}$ and predicts a move prior distribution $\mathbf{p}$ and the expected outcome v of the game,

$$\mathbf{p}, v = \mathbf{p}_{\boldsymbol{\theta}}(\mathbf{z}^0), v_{\boldsymbol{\theta}}(\mathbf{z}^0) = f_{\boldsymbol{\theta}}(\mathbf{z}^0) \ , \qquad [1]$$

both of which are used in Monte Carlo tree search.

A.1. Input encoding. In Figure S1, the input is $\mathbf{z}^0 \in \mathbb{R}^{8\times8\times(14h+7)}$ for a history length of h plies. If $h = 1$ and only the current position is represented, $\mathbf{z}^0 \in \mathbb{R}^{8\times8\times21}$. The first twelve 8×8 channels in $\mathbf{z}^0$ are binary, encoding the positions of the playing side and opposing side's king, queen(s), rooks, bishops, knights and pawns respectively. It is followed by 8×8 binary channels representing the number of repetitions (for three-fold repetition draws), the side to play, and four binary channels for whether the player and opponent can still castle king and queenside. Finally, the last two channels are an irreversible move counter (for 50 move rule) and total move counter, both scaled down. The input representation is always oriented toward the playing side, so that the board position with black to play is first flipped horizontally and vertically before being represented in the stack of 8×8 channels $\mathbf{z}^0$. Even though the state is fully captured with $h = 1$ when only the current position is encoded, there is a marginal empirical increase in performance when a few preceding positions are also incorporated into $\mathbf{z}^0$, and $\mathbf{z}^0 \in \mathbb{R}^{8\times8\times119}$ if the board positions of the last eight plies are stacked. Unless otherwise stated, $h = 8$ is used, following (2).

A.2. Layers. The network in Figure S1 has a residual neural network (ResNet) backbone (3), and every ResNet block will form a layer indexed by $d = 1, \ldots, D$. Each ResNet block contains internal layers, and in this paper we index layers at the points where the skip-connections meet. We denote the activations at layer d with $\mathbf{z}^d$, with $\mathbf{z}^0$ being the input. In the AlphaZero network, as illustrated in Figure S1, $\mathbf{z}^d \in \mathbb{R}^{8\times8\times256}$ for each $d = 1, \ldots, 20$. There are therefore 16384 activations at the end of each layer.

The network progressively transforms input $\mathbf{z}^0$ to $\mathbf{z}^1$, then $\mathbf{z}^2$, and so on through a series of residual blocks and final policy/value heads, as shown in Figure S1. The activations of layer d is given by the function $\mathbf{z}^d = f^d_{\boldsymbol{\theta}}(\mathbf{z}^{d-1})$, and hence $f^d_{\boldsymbol{\theta}} : \mathbb{R}^{8\times8\times256} \to \mathbb{R}^{8\times8\times256}$ for layers $d \geq 2$, and $f^1_{\boldsymbol{\theta}} : \mathbb{R}^{8\times8\times(14h+7)} \to \mathbb{R}^{8\times8\times256}$ in an initial convolution to 256 channels. We are going to omit the dependence of the layer on its parameters where it is clear from the context. For layers $d \geq 2$ the ResNet backbone in Figure S1 has the form

$$\mathbf{z}^d = f^d(\mathbf{z}^{d-1}) = \mathrm{ReLU}(\mathbf{z}^{d-1} + \gamma^d(\mathbf{z}^{d-1})) \; , \qquad [2]$$

which directly copies activations $\mathbf{z}^{d-1}$, adds an additional nonlinear function $\gamma^d(\mathbf{z}^{d-1})$ composed of two more convolution layers to it, and clips the result to be nonnegative through a rectified nonlinar unit (ReLU). For stability, activations $\mathbf{z}^d$ are additionally clipped to a maximum value of 15.

Layers 1 to 20 in Figure S1 form the 'torso' of the network. Two 'heads' complete the neural network by performing a computation on $\mathbf{z}^{20}$, the activations of the last layer in the torso. The 'value head' computes v in Eq. (1), while the 'policy head' computes $\mathbf{p}$, a distribution over all moves. The policy head, before flattening, produces a $8 \times 8 \times 73$ tensor. For every square, it encodes 73 possible moves to a next square: 7 horizontally left and right; 7 vertically up and down; 7 diagonal moves north west, north east, south west and south east; 8 knight moves; 3 promotion options to ♗, ♘, ♖ (a ♕ is default when a pawn reaches the eight rank) for the three single-square forward moves.

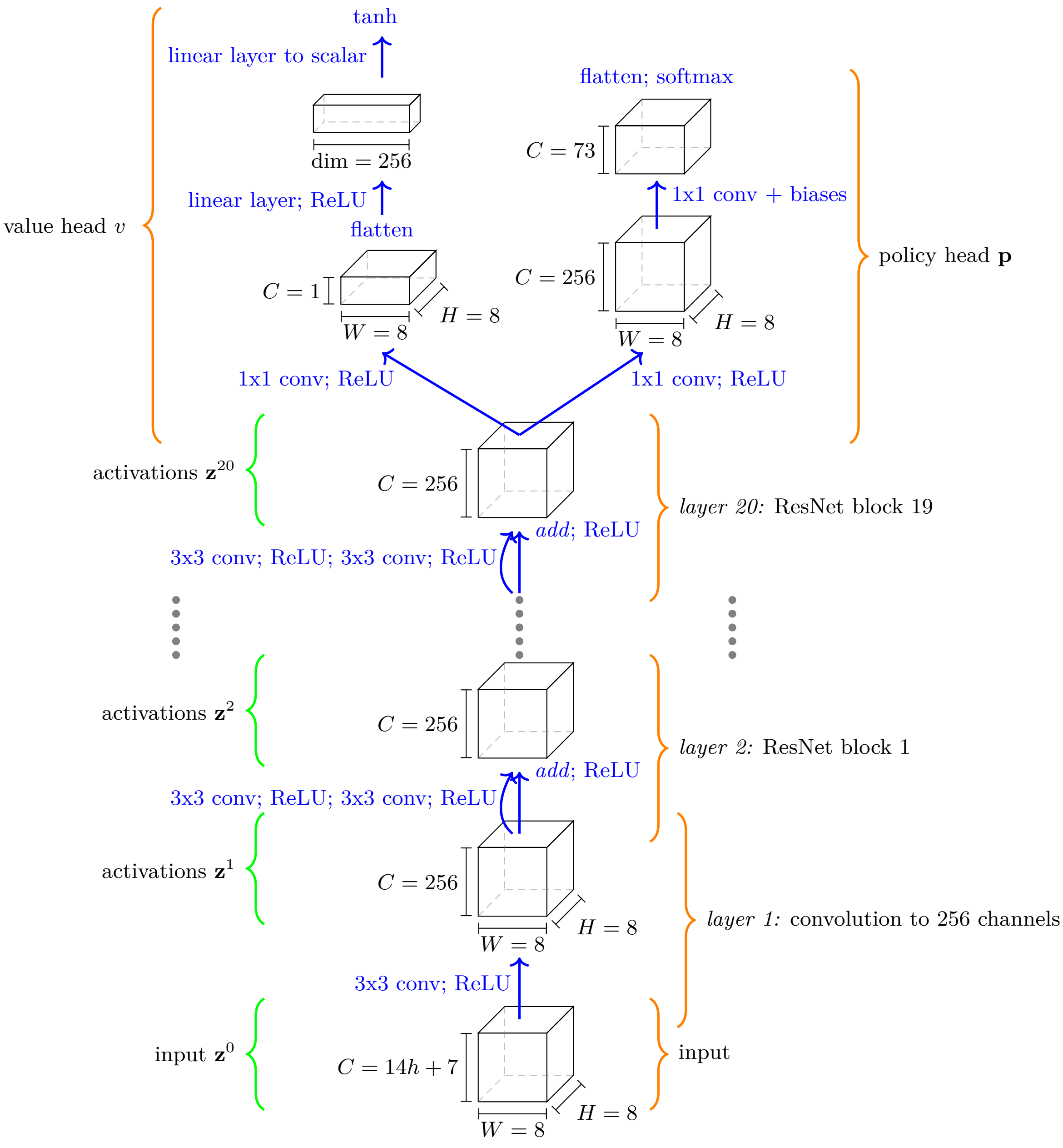

Fig. S1. The AlphaZero network. Each 3×3 convolution indicates the application of 256 filters of kernel size 3×3 with stride 1. A ResNet block contains two rectified batch-normalized convolutional layers with a skip connection. In the input $\mathbf{z}^0$, a history length of $h = 8$ plies is used, encoding the current board position and those of the seven preceding plies. The input is a $8 \times 8 \times 119$-dimensional tensor.

B. Network training.

Data generation by self-play Our experimental setup updates the parameters $\boldsymbol{\theta}$ of the AlphaZero network over 1,000,000 gradient descent training steps. A million steps is an arbitrary training time slightly longer than that of AlphaZero in (2). We will use t to index the training step, and $\boldsymbol{\theta}_t$ the network parameters after gradient descent step t.

The network is trained through positions with their associated MCTS move probability vectors that are sampled from self-play buffer containing the previous 1 million positions. At most 30 positions are sampled from a game on average, as positions on subsequent moves are strongly correlated, and including all of them may lead to increased overfitting. Stochastic gradient descent steps are taken with a batch size of 4096 in training. A synchronous decaying optimizer is used with initial learning rate 0.2, which is multiplied by 0.1 after 100k, 300k, 500k and 700k iterations.

After every 1000 training steps, the networks that are used to generate self-play games are refreshed, so that MCTS search uses a newer network. We refer to networks and their parameters $\boldsymbol{\theta}_t$ that are saved to disk at these points as 'checkpoints'. Self-play moves are executed upon reaching 800 MCTS simulations. Diversity in self-play is increased in two ways: through stochastic move sampling and through adding noise to the prior. The first thirty plies are sampled according to the softmax probability of the visit counts, and only after the thirtieth move are the moves with most visits in the MCTS simulations played deterministically. To further increase diversity in the self-play games, Dirichlet(0.3) noise is added to 25% of all priors $\mathbf{p}$ from Eq. (1) and renormalized. Of all self-play games, 20% are played out until the end, whereas in the remaining 80%, an early termination condition is introduced where a game is resigned if the value gives an expected score of 5% or less. The maximum game length is capped at 512 plies.

Training loss The network parameters $\boldsymbol{\theta}$ are updated using the gradient of the loss

$$\text{loss}(\boldsymbol{\theta}; \mathbf{z}^0) = -\boldsymbol{\pi}(\mathbf{z}^0)^T \log \mathbf{p}_{\boldsymbol{\theta}}(\mathbf{z}^0) + (v_{\boldsymbol{\theta}}(\mathbf{z}^0) - z)^2 + c\|\boldsymbol{\theta}\|_2^2, \qquad [3]$$

across a minibatch of $\mathbf{z}^0$-inputs drawn from the self-play buffer. The first term $-\boldsymbol{\pi}(\mathbf{z}^0)^T \log p_\theta(\mathbf{z}^0)$ trains the policy prior $\mathbf{p}$ to mimic the MCTS output $\boldsymbol{\pi}$ by minimising the Kullback-Leibler divergence between the two distributions. The second term trains the value head $v_{\boldsymbol{\theta}}$ to approximate the true return. The final term is L_2 regularisation, with c controlling the level of regularisation.

C. Move selection by Monte Carlo Tree Search (MCTS). Move selection is done by Monte Carlo tree search (MCTS), which runs repeated search simulations of how the game might unfold up to a pre-set maximum ply depth. A node in the tree is a state or position $\mathbf{z}^0$, and a branch is state-action pair $(\mathbf{z}^0, a)$, or position and a move that was played in the position. To be consistent with the paper, we donate a state by $\mathbf{z}^0$. Every branch tracks four numbers:

1. $N(\mathbf{z}^0, a)$, the visit count of the branch;
2. $W(\mathbf{z}^0, a)$, the total action-value, equal to the sum of backed-up leaf note v estimates;
3. $Q(\mathbf{z}^0, a) = W(\mathbf{z}^0, a)/N(\mathbf{z}^0, a)$, the mean action-value;
4. $P(\mathbf{z}^0, a) = \mathbf{p}_{\boldsymbol{\theta}}(\mathbf{z}^0)_a$, the prior probability of playing move a (selecting action a) in $\mathbf{z}^0$.

In one MCTS simulation, $f_{\boldsymbol{\theta}}$ is recursively applied to a sequence of positions until a maximum depth leaf node is reached. The sequence of moves in a simulation depends on an action selection criterion that is applied at each node along the path; it is version of the PUCT algorithm (4) that trades off exploration against revisiting more promising moves more frequently over consecutive simulations. The simulation begins at the root note of the search tree. If $\mathbf{z}_l^0$ indicates the position l plies deep in the search simulation, then the action is selected with

$$a_l = \arg\max_a \left(U(\mathbf{z}_l^0, a) + Q(\mathbf{z}_l^0, a) \right) . \qquad [4]$$

The action selection criterion has two terms; the U-term encourages exploration, while the Q term exploits the best move found thus far. The action selection criterion is such that before a state is first encountered in a simulation, its resulting prior vector $\mathbf{p}$ assigns weights to candidate moves at a "first glance" of the board. It can be seen in the exploration term

$$U(\mathbf{z}^0, a) = C(\mathbf{z}^0)\, P(\mathbf{z}^0, a)\, \frac{\sqrt{N(\mathbf{z}^0)}}{1 + N(\mathbf{z}^0, a)} , \qquad [5]$$

where $N(\mathbf{z}^0)$ is the branch's parent node visit count. The exploration rate $C(\mathbf{z}^0) = \log((1 + N(\mathbf{z}^0) + c_{\text{base}})/c_{\text{base}}) + c_{\text{init}}$, which is essentially constant for fast training games (settings $c_{\text{init}} = 1.25$ and $c_{\text{base}} = 19652$ were used).

When a leaf node is reached, its position's evaluation v is "backed up" to the root, with each node along the path incrementing its visit count $N(\mathbf{z}^0, a) \leftarrow N(\mathbf{z}^0, a) + 1$, and including the leaf's v in its action-value estimate, $W(\mathbf{z}^0, a) \leftarrow W(\mathbf{z}^0, a) + v$. After a number of such MCTS simulations, the root move that was visited most is played.

2. Full concept list

Table S1 summarizes 93 concepts from Stockfish 8's public API. The concepts are taken from Stockfish 8 and not a later version, as there is a wealth of observations on the difference between AlphaZero and Stockfish 8 (5). We implemented a long list of additional concepts programatically, and these are presented in Tables S2 and S3.

Table S1. A summary of 93 concepts from Stockfish 8's public API. The concepts are enumerated as <concept_name>_<side>_<game_phase>, where the side is `[w|b|t]` for White, Black, total (difference) respectively. The game phase abbreviations `[mg|eg|ph]` stands for middle game, end game and phased value respectively. The phased value is a weighted sum of the middle and end game values based on the actual phase of a given position. As AlphaZero represents positions from the playing side's view, "side" is orientated and represented as `mine` and `opponent` instead of `w` and `b`.

Concept names	Description
`material [t] [mg|eg|ph]`	Material score, where each piece on the board has a predefined value that changes depending on the phase of the game. The value of each piece is computed in isolation, independently from other pieces on the board. E.g., at a given phase of the game, the value of a knight doesn't depend on whether the opponent has a pair of bishops, an extra pawn or a rook. Compare this concept to `imbalance`.
`imbalance [t] [mg|eg|ph]`	Imbalance score compares each piece value with respect to all other pieces on the board. Rather than consider the value of a *single* piece depending on position and phase, imbalance accounts for the way that other pieces affect the value of a given piece. It allows adjusting the `material` score of each piece by favoring or penalizing particular imbalances in piece counts between the players. E.g., the score favors having a pair of bishops vs. a bishop and a knight. Compare it to the `material` score that assigns value to individual pieces in isolation from other pieces. Computed as a quadratic form of piece counts with pre-set weight matrix.
`pawns [t] [mg|eg|ph]`	Evaluation of the pawn structure. E.g., the evaluation considers isolated, double, connected, backward, blocked, weak, etc. pawns.
`knights [t] [mg|eg|ph]`	Evaluation of knights. E.g., extra points are given to knights that occupy outposts protected by pawns.
`bishops [t] [mg|eg|ph]`	Evaluation of bishops. E.g., bishops that occupy the same color squares as pawns are penalised.
`rooks [t] [mg|eg|ph]`	Evaluation of rooks. E.g., rooks that occupy open or semi-open files have higher valuation.
`queens [t] [mg|eg|ph]`	Evaluation of queens. E.g., queens that have relative pin or discovered attack against them are penalized.
`mobility [t] [mg|eg|ph]`	Evaluation of piece mobility score. It depends on the number of squares attacked by the pieces.
`king_safety [t] [mg|eg|ph]`	A complex concept related to king safety. It depends on the number and type of pieces that attack squares around the king, shelter strength, number of pawns around the king, penalties for being on pawnless flank, etc.
`threats [t] [mg|eg|ph]`	Evaluation of threats to pieces, such as whether a pawn can safely advance and attack an opponent's higher value piece, hanging pieces, possible xray attacks by rooks, etc.
`passed_pawns [t] [mg|eg|ph]`	Evaluates bonuses for passed pawns. The closer a pawn is to the promotion rank, the higher is the bonus.
`space [t] [mg|eg|ph]`	Evaluation of the space. It depends on the number of safe squares available for minor pieces on the central four files on ranks 2 to 4.
`total [t] [mg|eg|ph]`	The total evaluation of a given position. It encapsulates all the above concepts.

Table S2. Custom chess concepts (self implemented; i.e. not from Stockfish 8's API) used in this paper. We use `m` as shorthand for `mine` and `o` as shorthand for `opponent`. `diff` stands for the `difference` between the mine and opponent values of the same concept.

<table>
<tr><th>Concept names</th><th>Description</th></tr>
<tr><td>pawn_fork [m|o]</td><td>True if a pawn is attacking two pieces of higher value (knight, bishop, rook, queen, or king) and is not pinned.</td></tr>
<tr><td>knight_fork [m|o]</td><td>True if a knight is attacking two pieces of higher value (rook, queen, or king) and is not pinned.</td></tr>
<tr><td>bishop_fork [m|o]</td><td>True if a bishop is attacking two pieces of higher value (rook, queen, or king) and is not pinned.</td></tr>
<tr><td>rook_fork [m|o]</td><td>True if a rook is attacking two pieces of higher value (queen, or king) and is not pinned.</td></tr>
<tr><td>has_pinned_pawn [m|o]</td><td>True if the side has a pawn that is pinned to the king of the same colour.</td></tr>
<tr><td>has_pinned_knight [m|o]</td><td>True if the side has a knight that is pinned to the king of the same colour.</td></tr>
<tr><td>has_pinned_bishop [m|o]</td><td>True if the side has a bishop that is pinned to the king of the same colour.</td></tr>
<tr><td>has_pinned_rook [m|o]</td><td>True if the side has a rook that is pinned to the king of the same colour.</td></tr>
<tr><td>has_pinned_queen [m|o]</td><td>True if the side has a queen that is pinned to the king of the same colour.</td></tr>
<tr><td>material [m|o|diff]</td><td>Material calculated as (#♙) + 3 × (#♘) + 3 × (#♗) + 5 × (#♖) + 9 × (#♕)</td></tr>
<tr><td>num_pieces [m|o|diff]</td><td>Number of pieces that a side has.</td></tr>
<tr><td>in_check</td><td>True if the side that makes a turn is in check.</td></tr>
<tr><td>has_bishop_pair [m|o]</td><td>True if the side has a pair of bishops.</td></tr>
<tr><td>has_connected_rooks [m|o]</td><td>True if the side has connected rooks.</td></tr>
<tr><td>has_control_of_open_file [m|o]</td><td>True if the side controls an open file (with the rooks, queen)</td></tr>
<tr><td>has_mate_threat</td><td>True if the opponent could mate the current side in a single move if the turn was passed to the opponent.</td></tr>
<tr><td>has_check_move [m|o]</td><td>True if the side can check the opponent's King.</td></tr>
<tr><td>can_capture_queen [m|o]</td><td>True if the side can capture the opponent's queen.</td></tr>
<tr><td>num_king_attacked_squares [m|o|diff]</td><td>The number of squares around the opponent's king that the playing side attacks. Can include occupied squares.</td></tr>
<tr><td>has_contested_open_file</td><td>True if an open file is occupied simultaneously by a rook and/or queen of both colours.</td></tr>
<tr><td>has_right_bc_ha_promotion [m|o]</td><td>True if 1) the side has a passed pawn on a or h files and 2) the side has a bishop that is of the colour of the promotion square of that pawn.</td></tr>
<tr><td>num_scb_pawns_same_side [m|o|diff]</td><td>The number of own pawns that occupy squares of the same colour as the colour of own bishop. Applicable only when the side has a single bishop.</td></tr>
<tr><td>num_ocb_pawns_same_side [m|o|diff]</td><td>The number of own pawns that occupy squares of the opposite colour to that of own bishop. Applicable only when the side has a single bishop.</td></tr>
<tr><td>num_scb_pawns_other_side [m|o|diff]</td><td>The number of opponent's pawns that occupy the squares of the same colour as the colour of own bishop. Applicable only when the side has a single bishop.</td></tr>
<tr><td>num_ocb_pawns_other_side [m|o|diff]</td><td>The number of opponent's pawns that occupy the squares of the opposite colour to the colour of own bishop. Applicable only when the side has a single bishop.</td></tr>
<tr><td>capture_possible_on_{sq} [m|o]
sq=[d1|d2|d3|e1|e2|e3|g5|b5]</td><td>True is the side can capture a piece on the given square.
The squares are named as if the side were playing White.</td></tr>
<tr><td>capture_happens_next_move_ ...
...on_{sq}
sq=[d1|d2|d3|e1|e2|e3|g5|b5]</td><td>True if the capture of a piece on the given square had happened according to the game data. The squares are named as if the side were playing White.</td></tr>
</table>

Table S3. Custom chess concepts related to pawns (self implemented; i.e. not from Stockfish 8's API) used in this paper. We use `m` as shorthand for `mine` and `o` as shorthand for `opponent`. `diff` stands for the `difference` between the mine and opponent values of the same concept.

Concept names	Description
num_double_pawn_files [t] [m|o|diff]	Number of files that contain one or more pawns of a given colour.
has_double_pawn [m|o]	True if a file contains one or more pawns of a given colour.
num_isolated_pawns [m|o|diff]	Number of pawns that have no friendly pawns in the files to their left and right.
has_isolated_pawn [m|o]	True if there is a pawn that has no pawns in the file to their left or right.
has_pawn_on_7th_rank [m|o]	True if the side has a pawn that reached the 7th rank.
pawns_on_7th_rank [m|o|diff]	Number of pawns that reached the 7th rank.
has_passed_pawn [m|o]	True if the side has a pawn with no opposing pawns to prevent it from advancing to the eighth rank.
num_passed_pawns [m|o|diff]	The number of passed pawns.
has_protected_passed_pawn [m|o]	True if the side has a passed pawn that is protected by its own pawn.
num_protected_passed_pawns [m|o|diff]	The number of protected passed pawns.
num_pawn_islands [m|o|diff]	The number of pawn islands.
has_iqp [m|o]	True if the side has an isolated queen's pawn (d file).
has_connected_passed_pawns [m|o]	True if the side has two or more passed pawns on adjacent files.
num_connected_passed_pawns [m|o|diff]	The number of connected passed pawns that the side has.

3. Regression results for all concepts

In our experiments, we have considered a large number of potential human chess concepts within a set of concepts we have tried to identify, localize, and explore the acquisition of within the AlphaZero chess model. Even this extended list is far from being able to explicitly capture the vast chess knowledge that has accumulated over centuries and the multitude of patterns that can appear on the board. It is merely a starting point for further exploration.

While we focused our discussion in the main text on a smaller number of relevant human concepts and their regression from different layers in the AlphaZero network over time, we present the results for the extended list of concepts we've used in our experiments here in Figures S2 to S13.

A. Regression results for Stockfish concepts from Table S1.

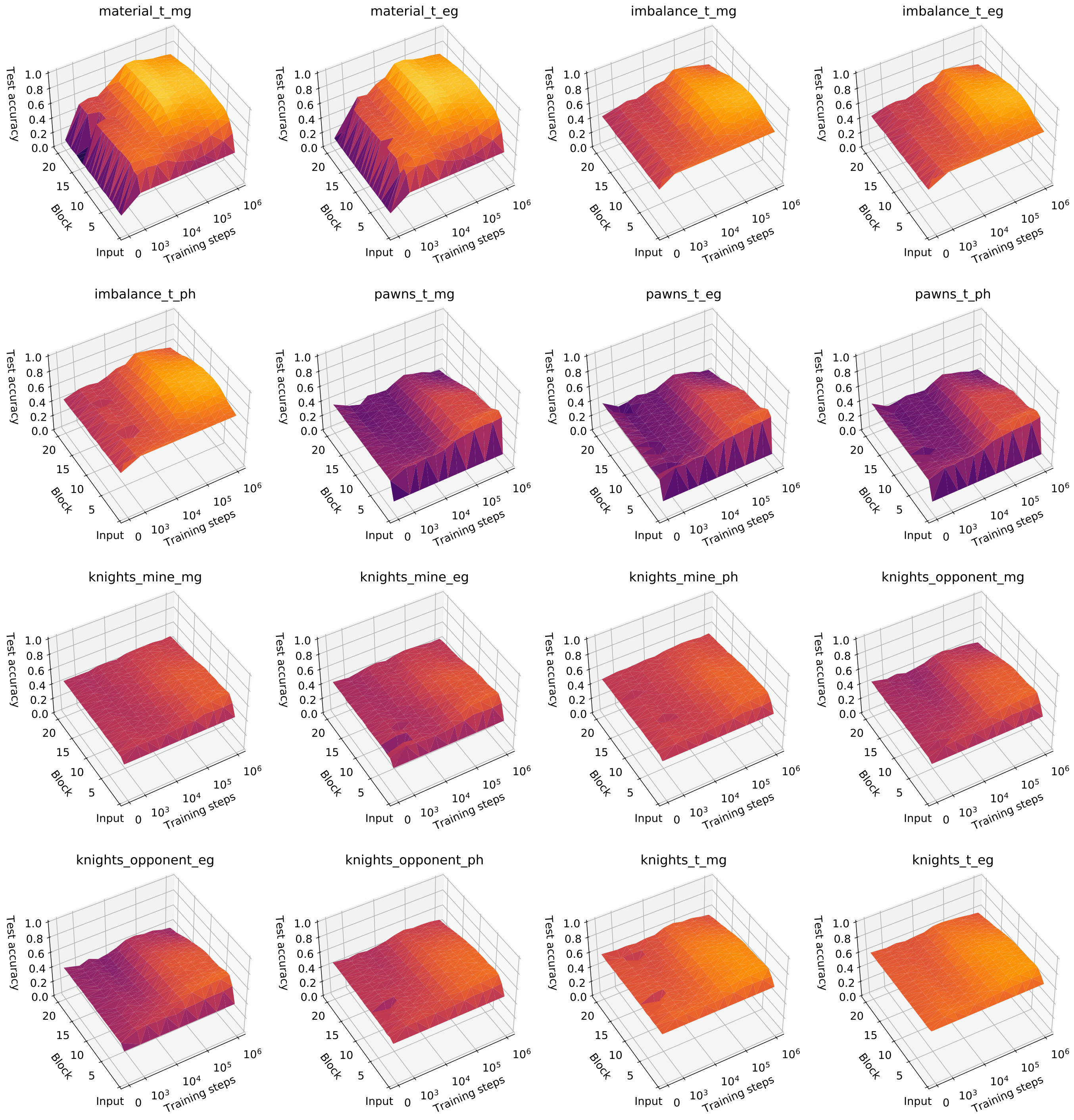

Fig. S2. Regression results for Stockfish concepts from Table S1.

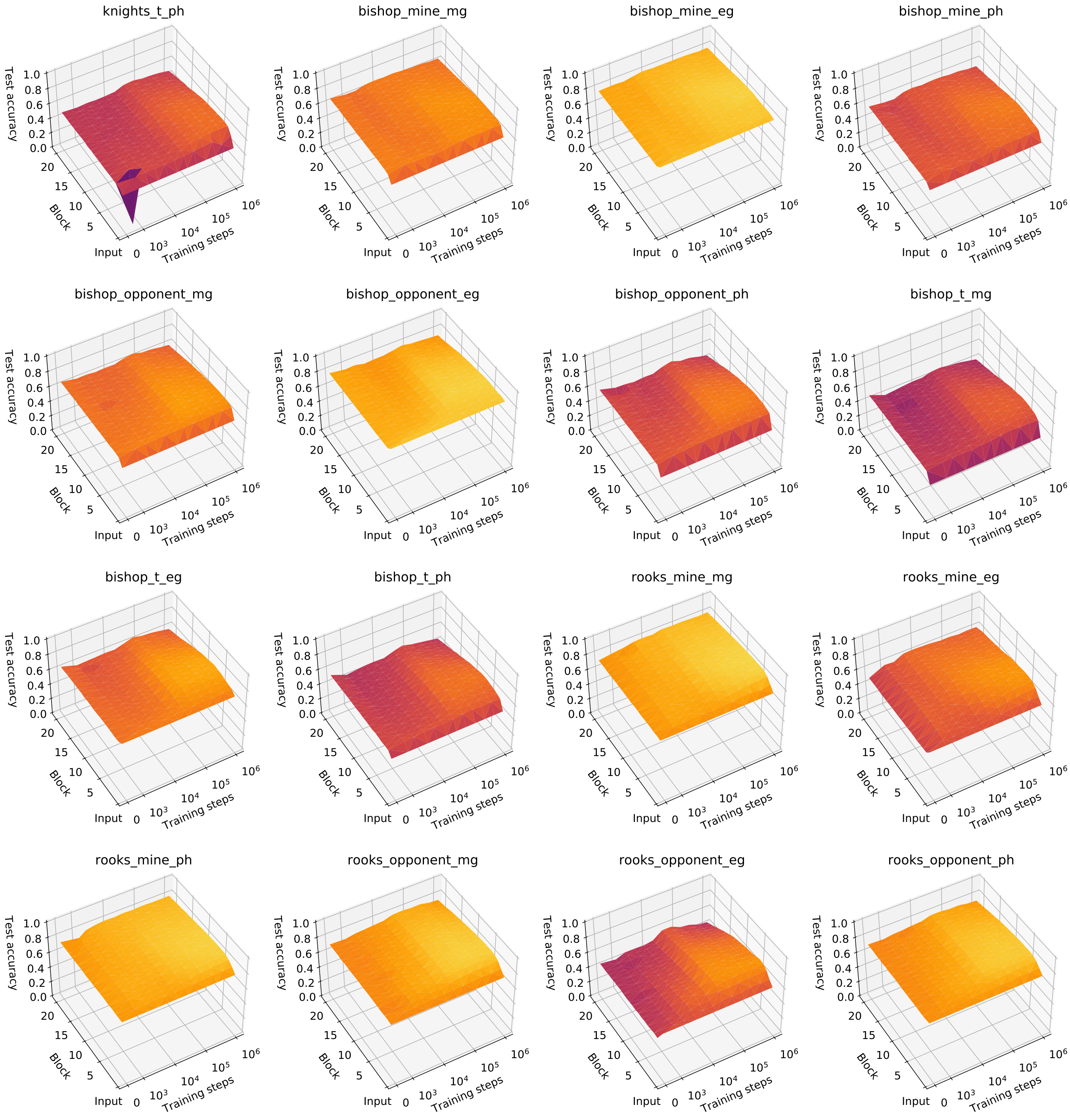

Fig. S3. Regression results for Stockfish concepts from Table S1, continued.

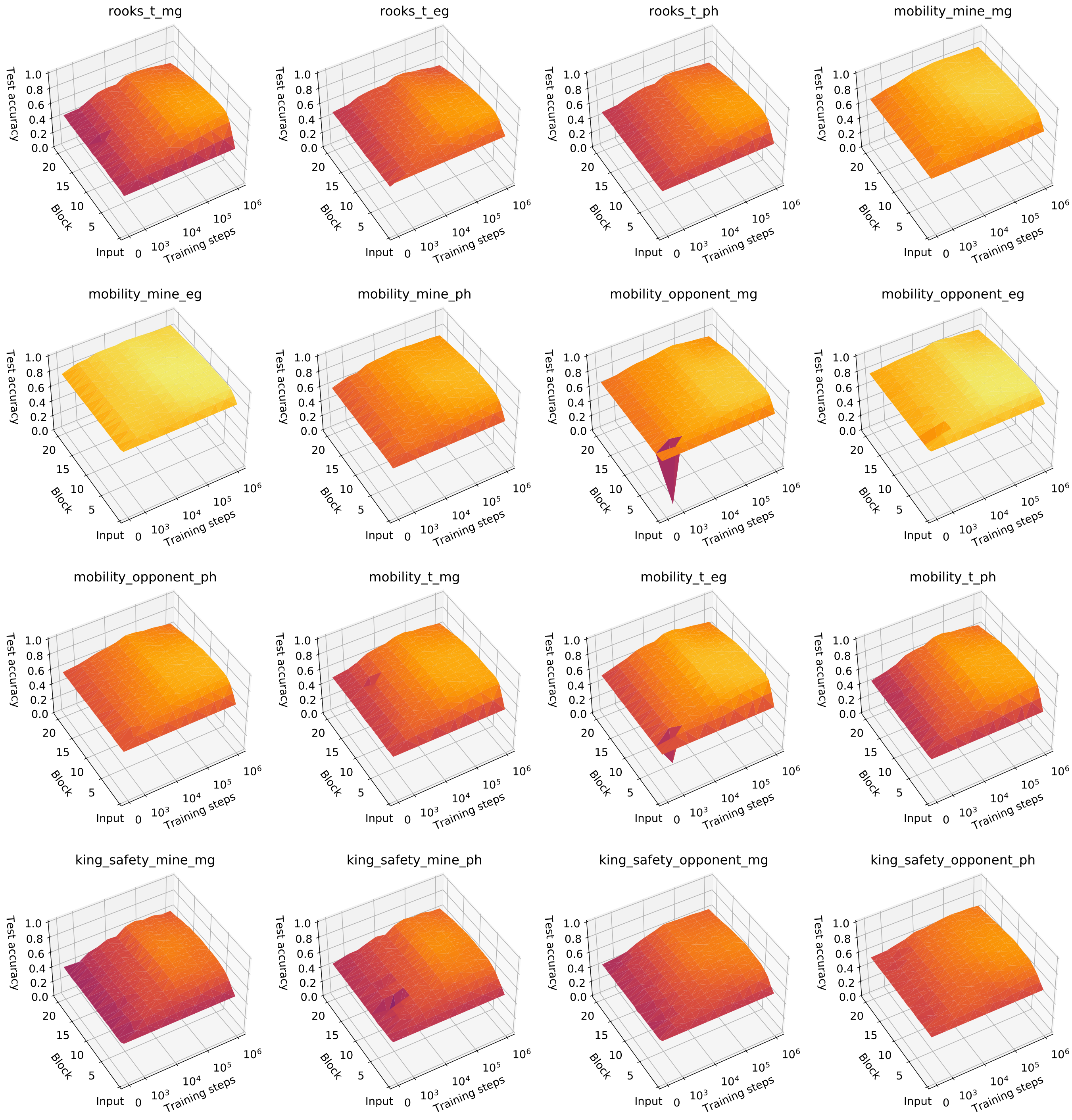

Fig. S4. Regression results for Stockfish concepts from Table S1, continued.

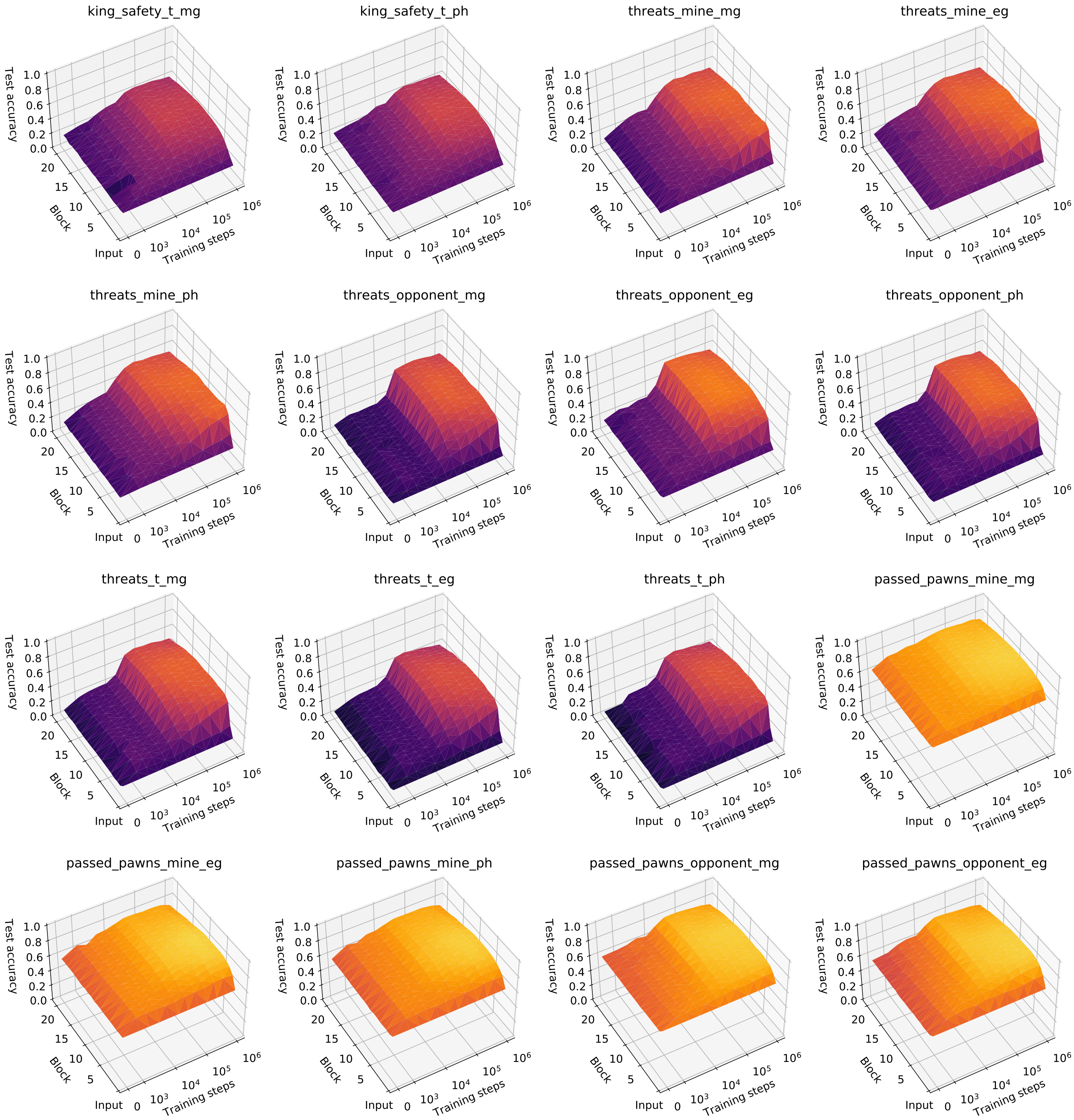

Fig. S5. Regression results for Stockfish concepts from Table S1, continued.

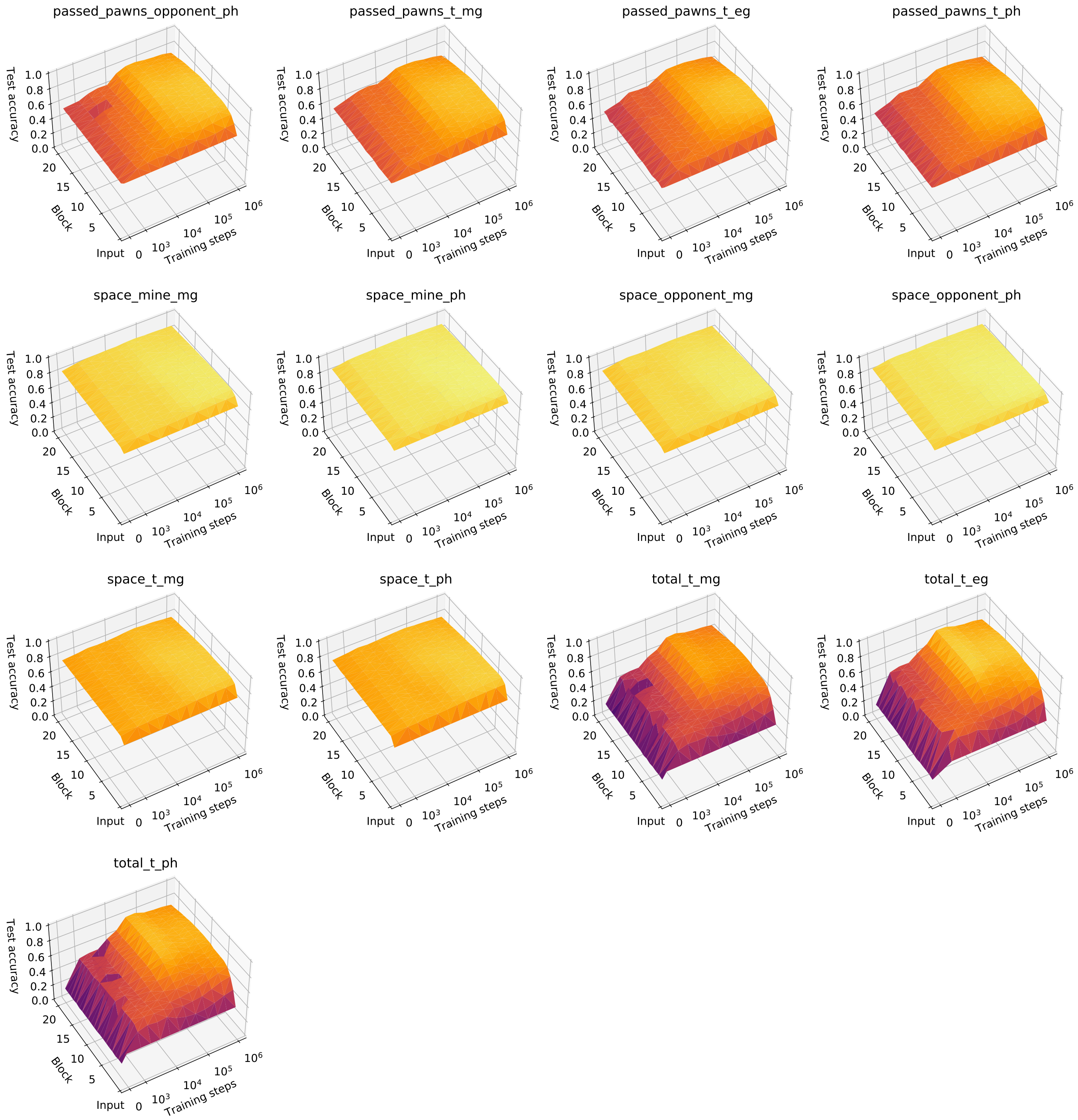

Fig. S6. Regression results for Stockfish concepts from Table S1, continued.

B. Regression results for custom concepts from Table S2, excluding capture-related concepts.

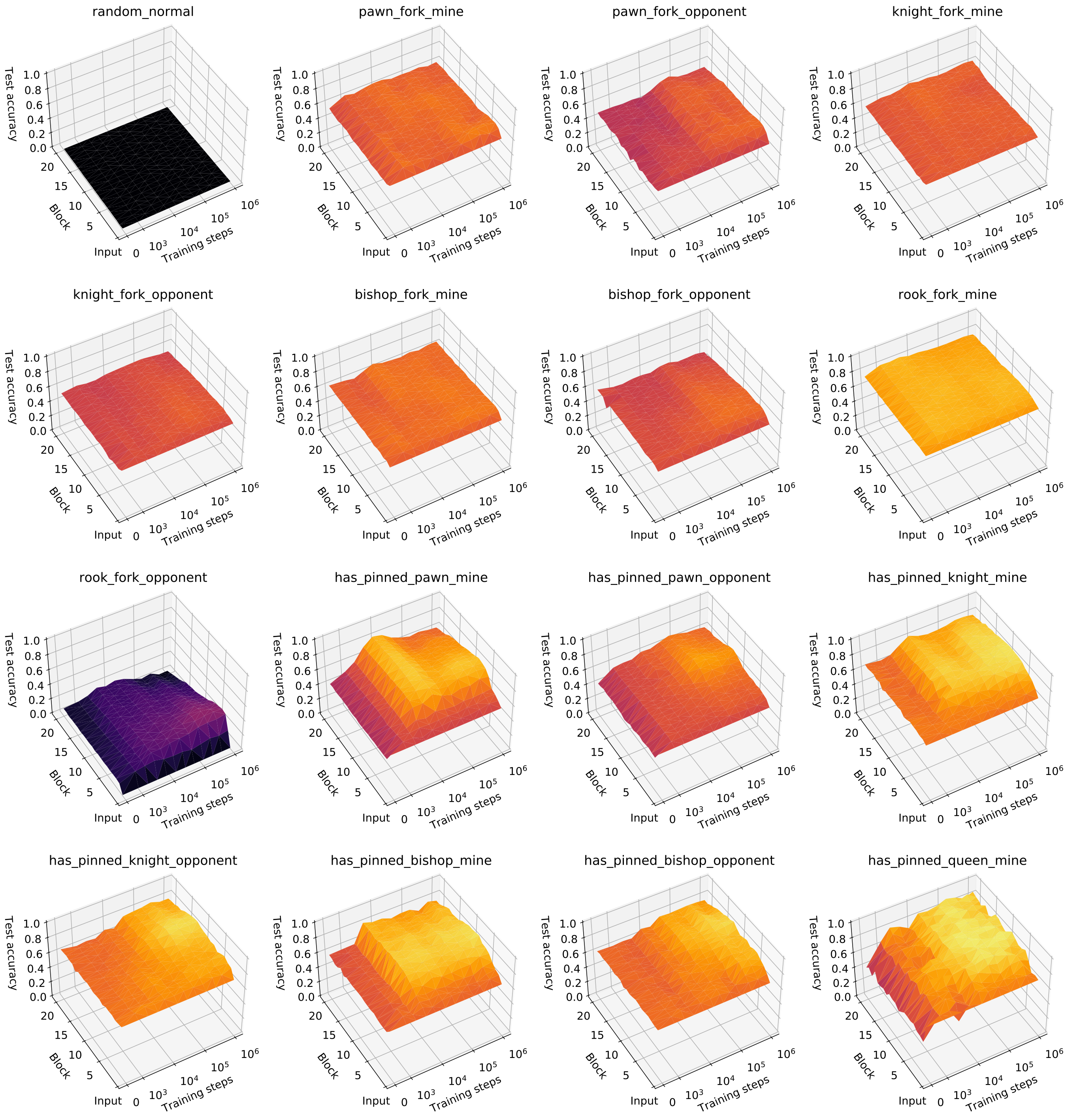

Fig. S7. Regression results for custom concepts from Table S2, excluding capture-related concepts. `random_normal` refers to random Gaussian labels with zero mean and variance of one.

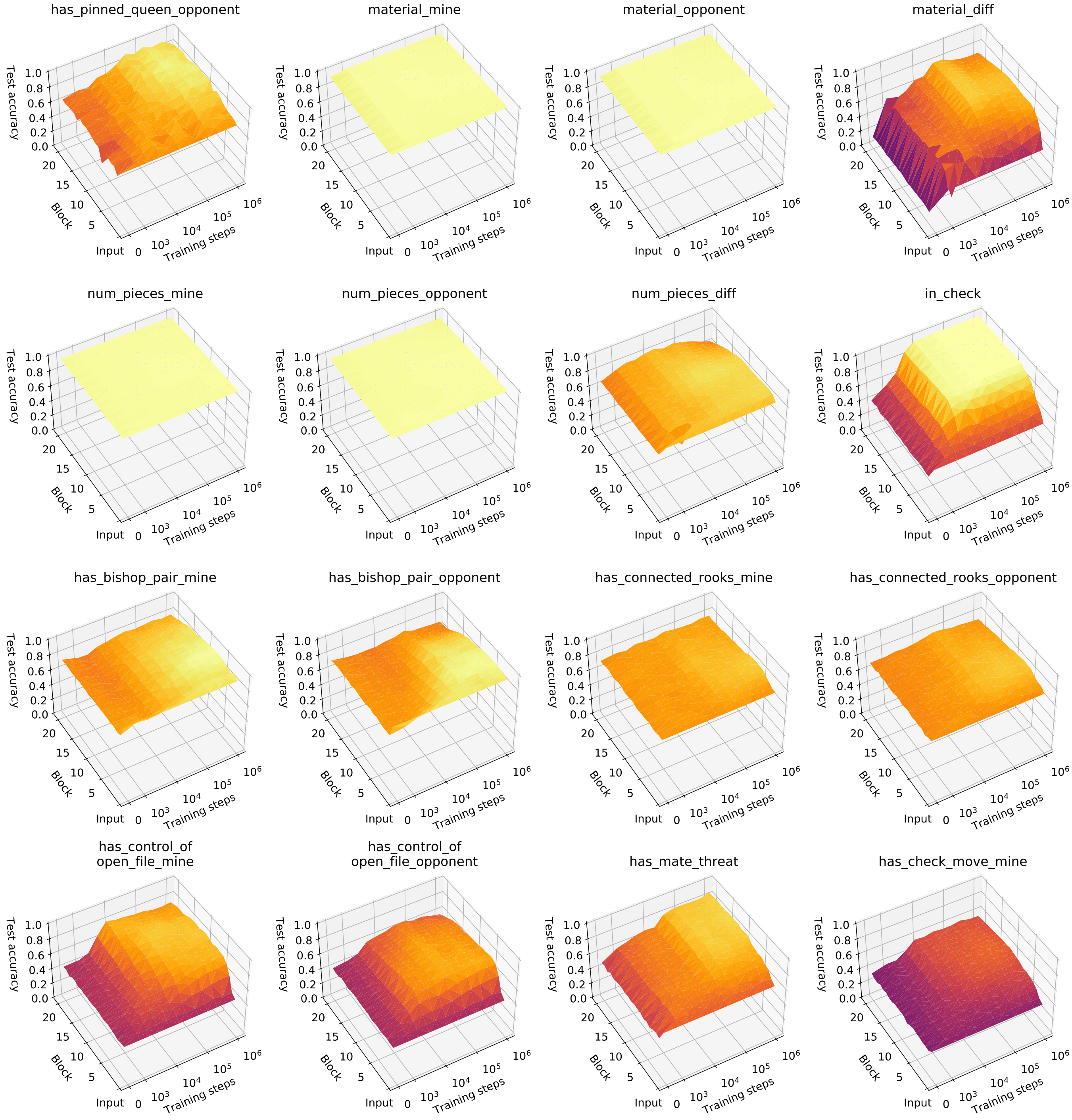

Fig. S8. Regression results for custom concepts from Table S2, excluding capture-related concepts, continued.

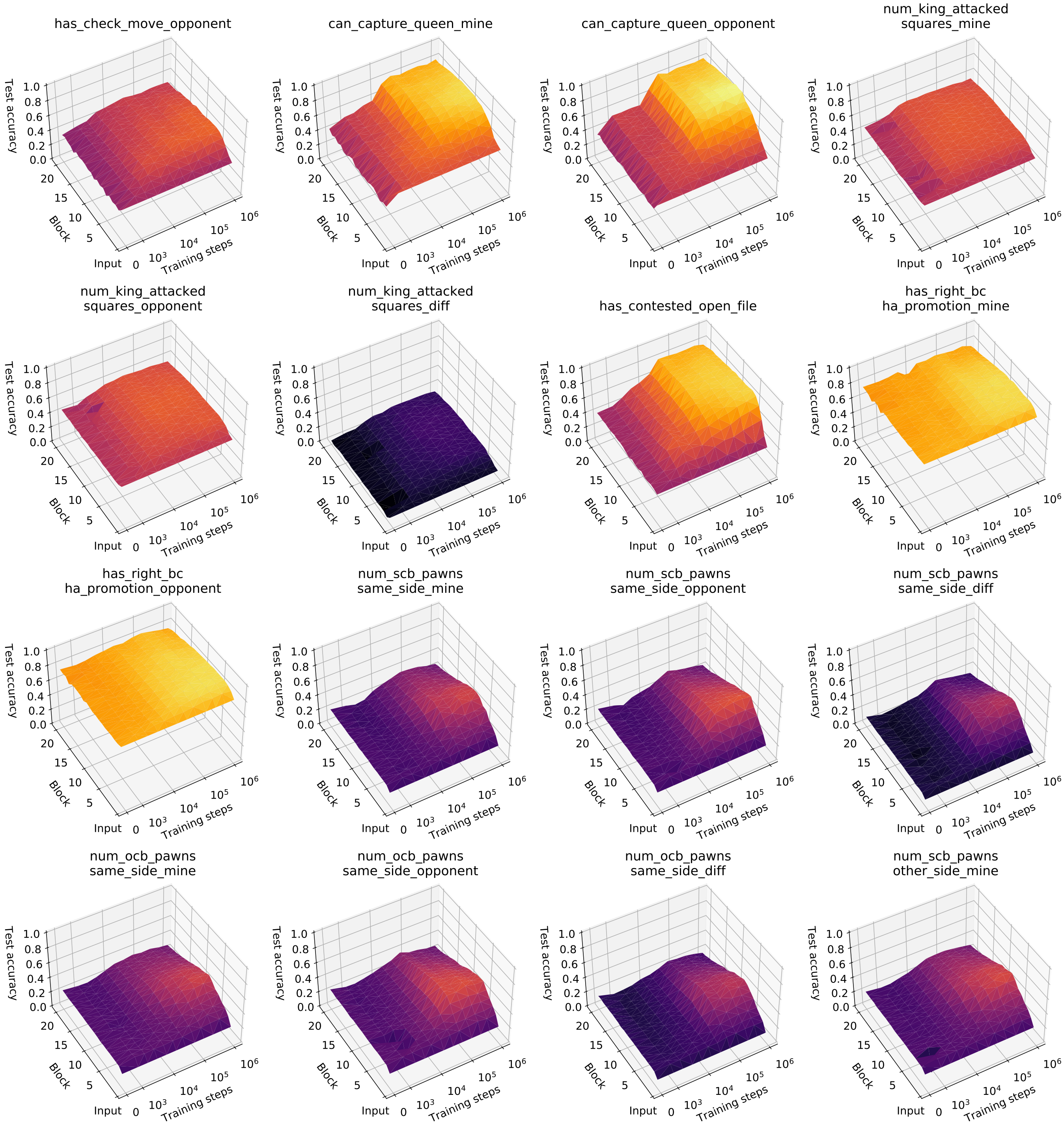

Fig. S9. Regression results for custom concepts from Table S2, excluding capture-related concepts, continued.

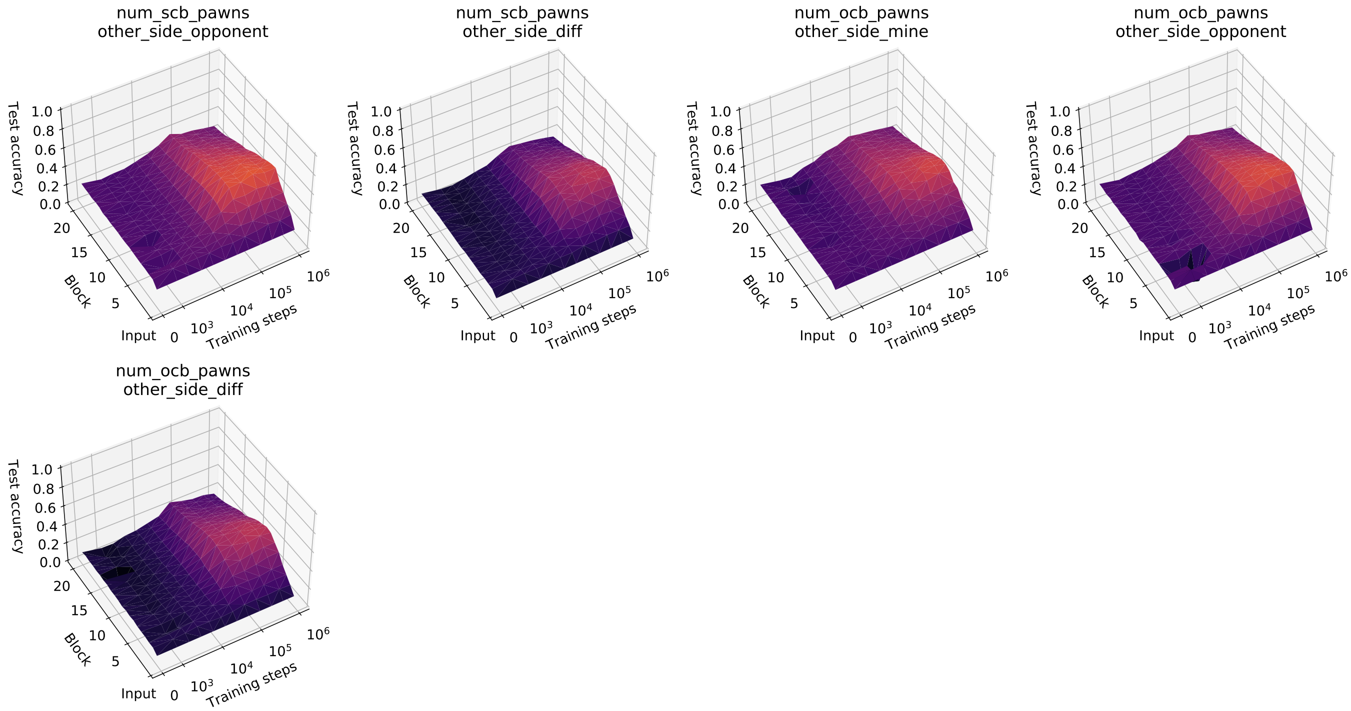

Fig. S10. Regression results for custom concepts from Table S2, excluding capture-related concepts, continued.

C. Regression results for custom concepts from Table S2 related to captures.

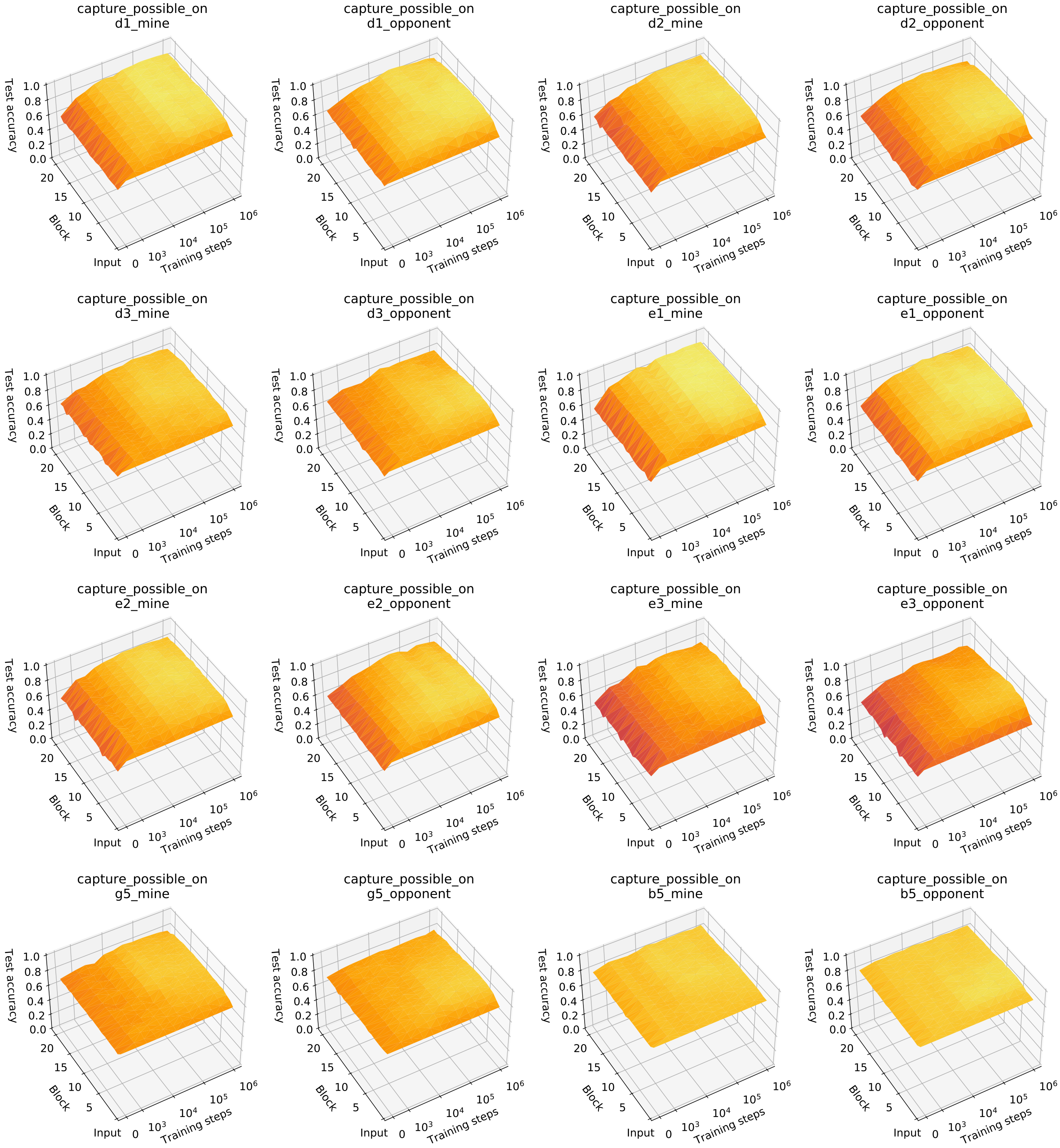

Fig. S11. Regression results for custom concepts from Table S2 related to captures.

D. Regression results for custom pawn-related concepts from Table S3.

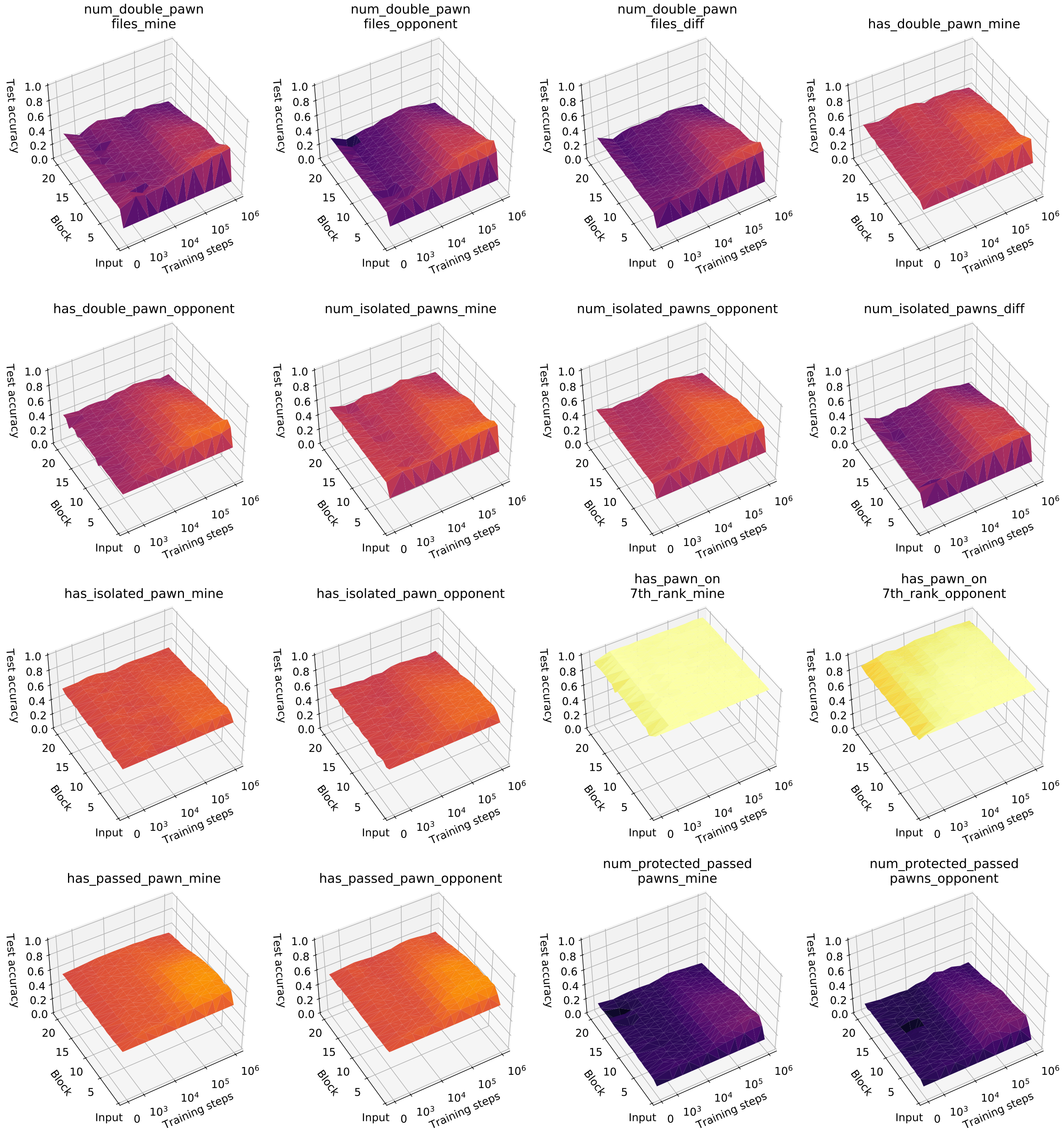

Fig. S12. Regression results for custom pawn-related concepts from Table S3.

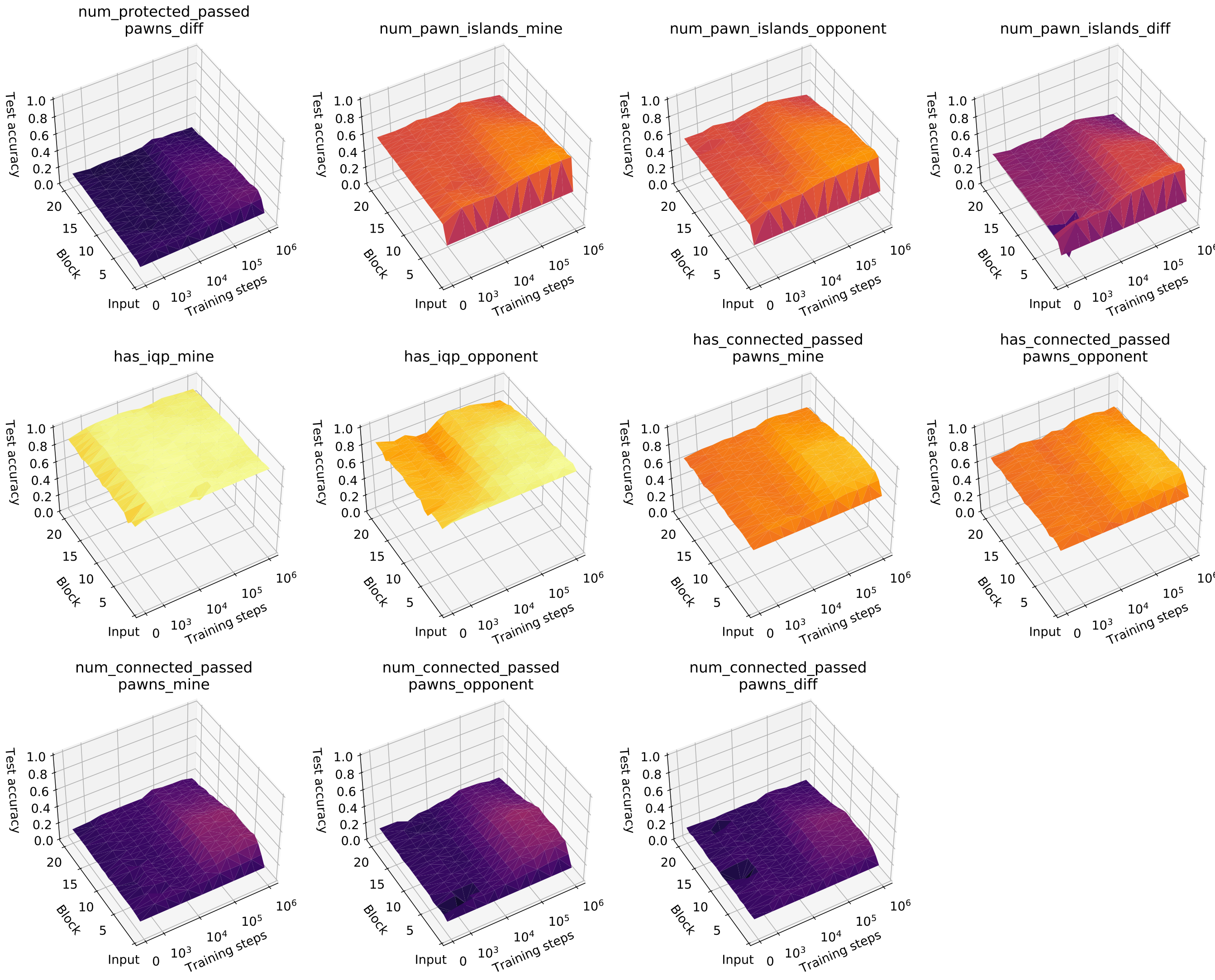

Fig. S13. Regression results for custom pawn-related concepts from Table S3, continued.

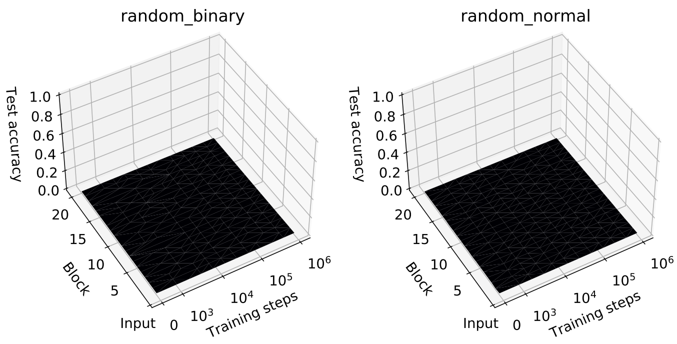

Fig. S14. Regression results for random baselines.

4. AlphaZero policy progression through training for different training seeds

Figures S15 to S20 show the distribution of move sequences with the highest probabilities as predicted by the policy network. Every figure provides the sequence start position (a) and the probability distribution for different initial training seeds (c)-(f). The x axis shows the training iteration for the given seed. The white area on the plots show the cumulative probability of all move sequences that are not included in (b). Only moves that were made at least once by human players were considered.

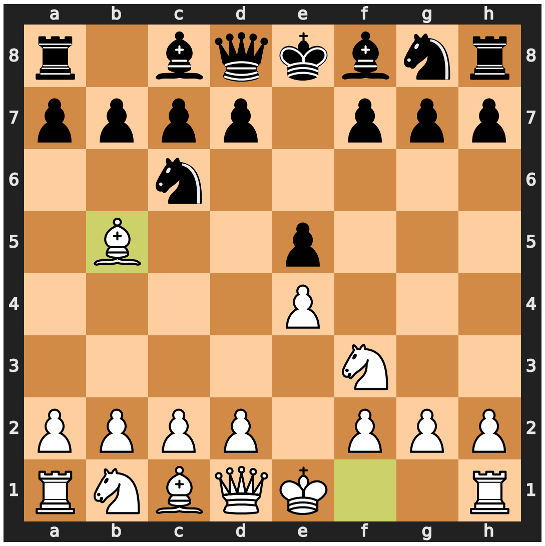

(a) Sequence start position.

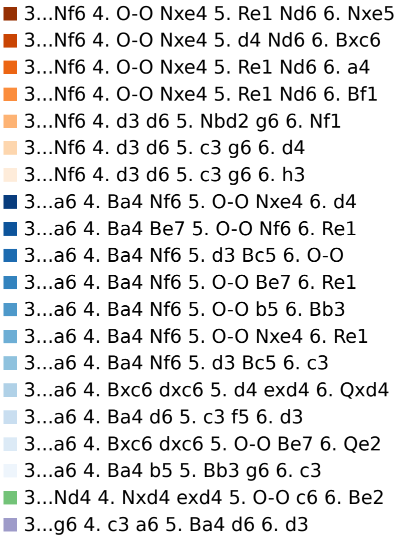

(b) Legend.

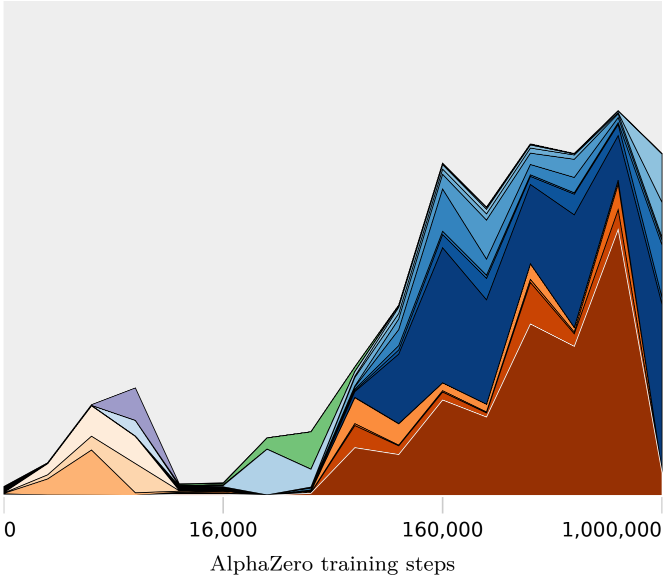

(c) Training seed 1.

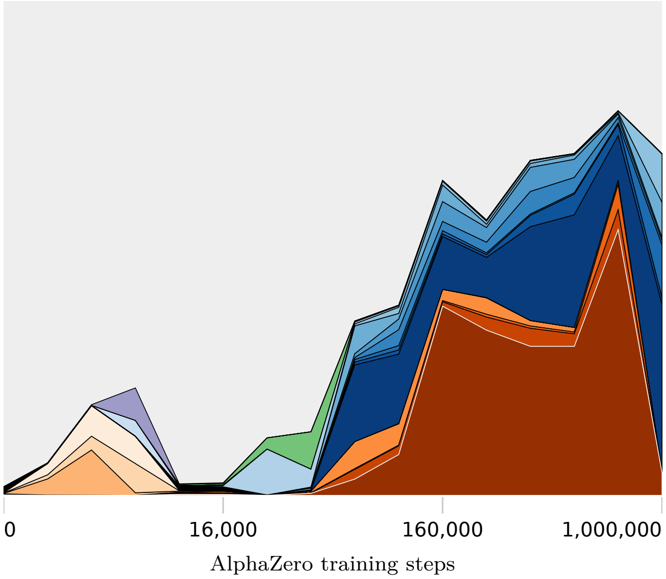

(d) Training seed 2.

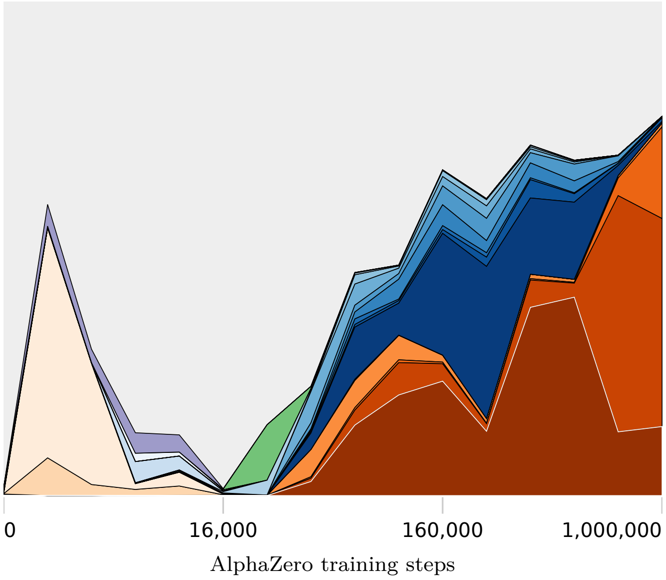

(e) Training seed 3.

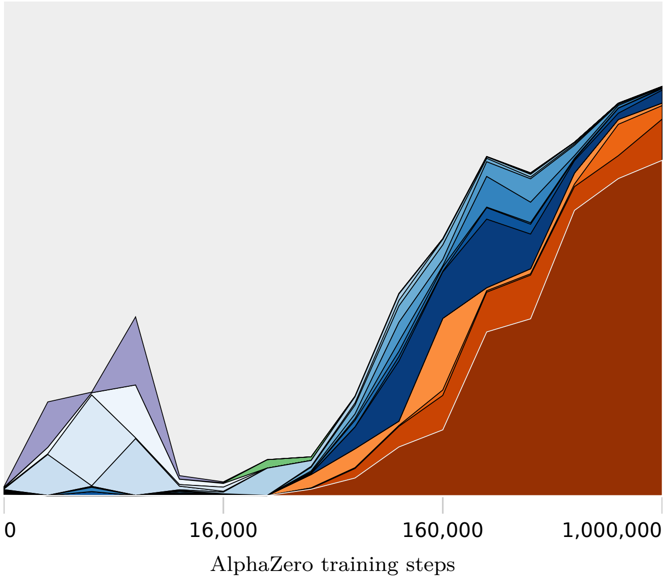

(f) Training seed 4.

Fig. S15. Top 20 move sequences with the highest joint probability after 1. e4 e5 2. ♘f3 ♘c6 3. ♗b5.

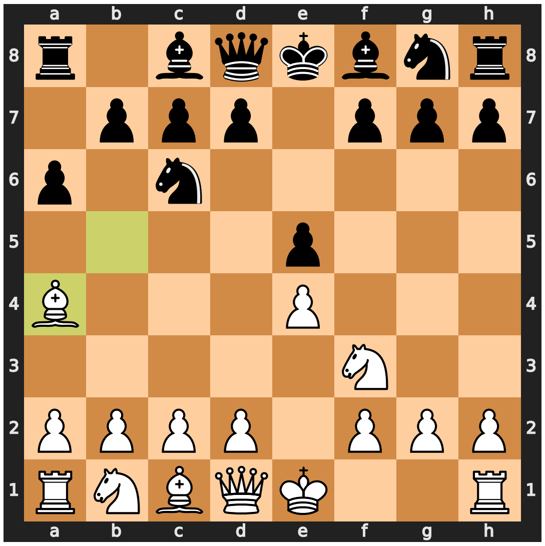

(a) Sequence start position.

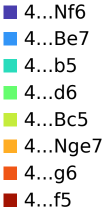

(b) Legend.

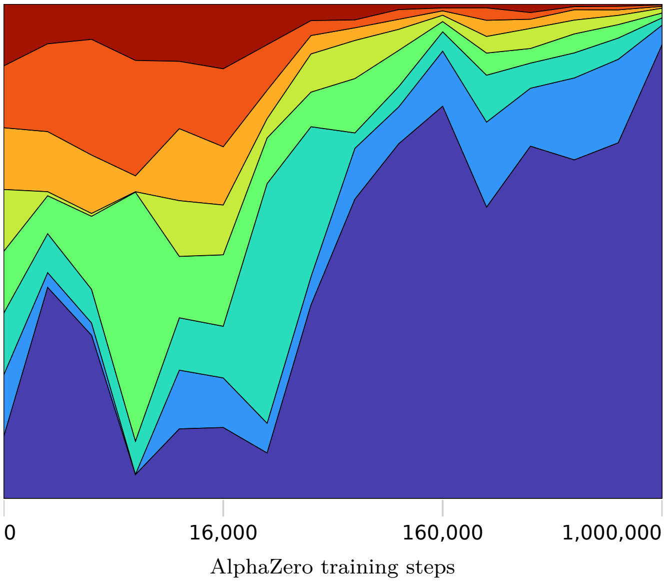

(c) Training seed 1.

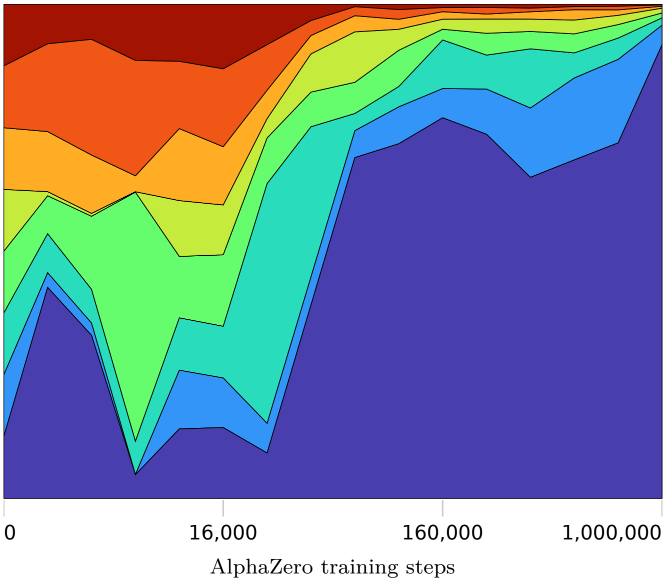

(d) Training seed 2.

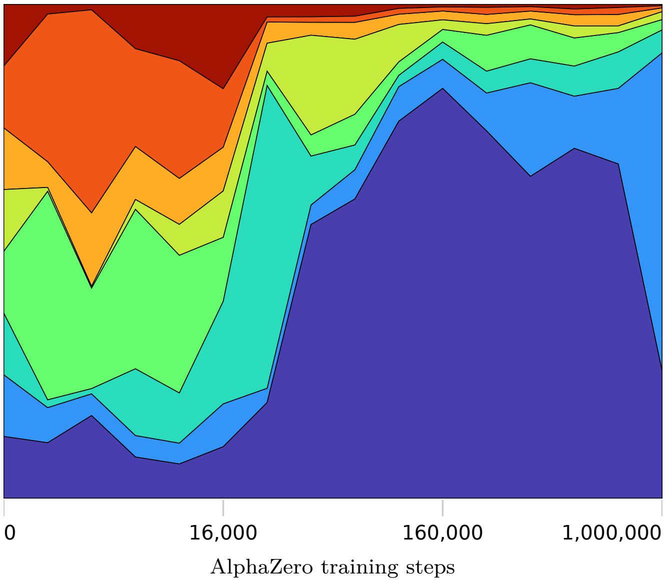

(e) Training seed 3.

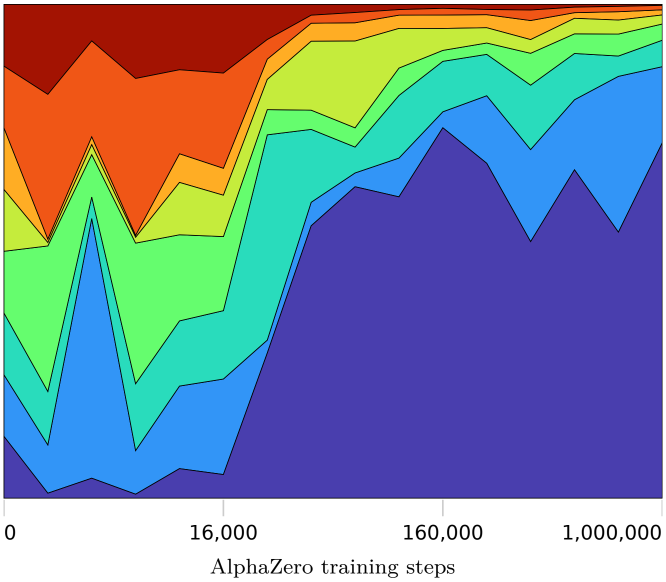

(f) Training seed 4.

Fig. S16. Top 8 moves with the highest probability after 1. e4 e5 2. ♘f3 ♘c6 3. ♗b5 a6 4. ♗a4.

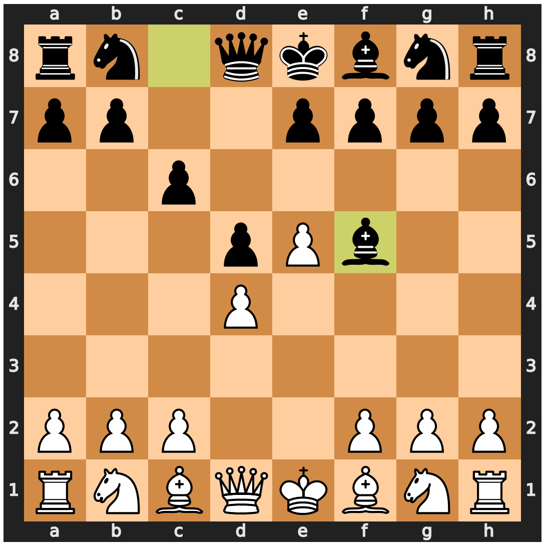

(a) Sequence start position.

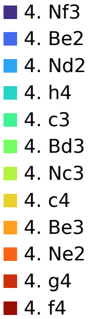

(b) Legend.

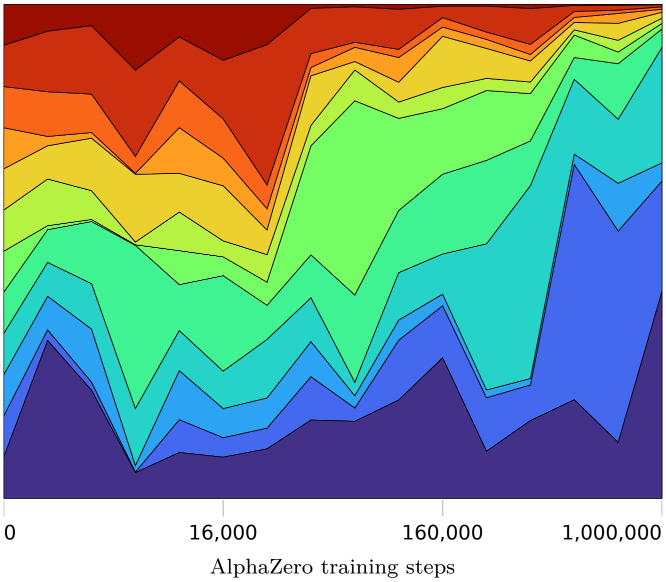

(c) Training seed 1.

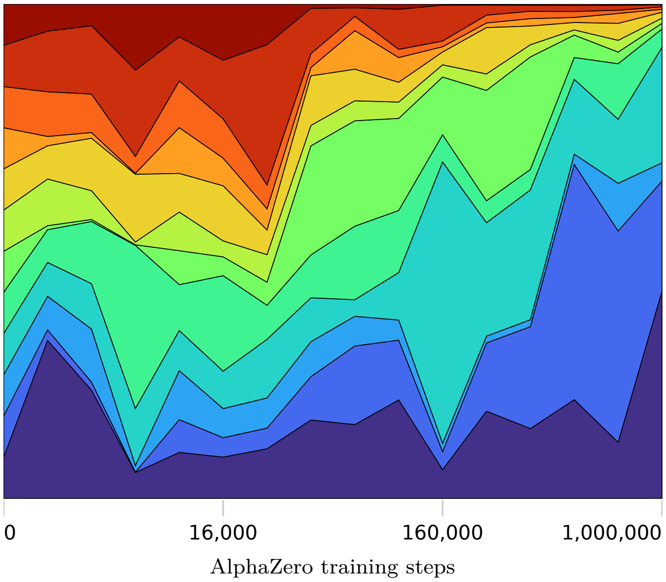

(d) Training seed 2.

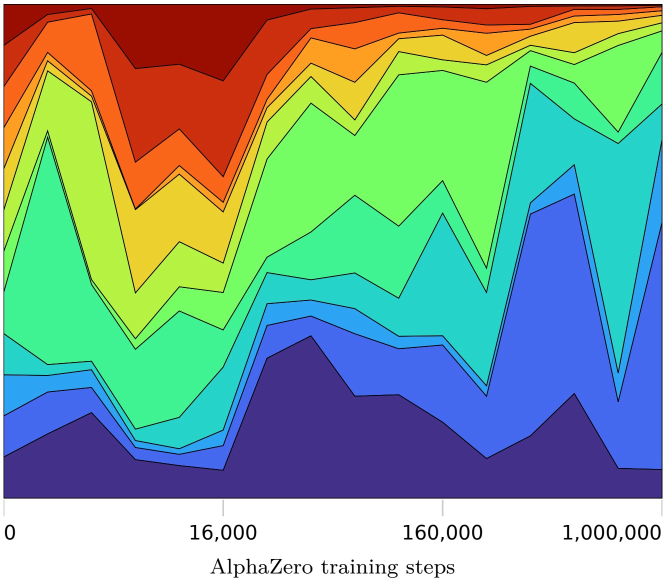

(e) Training seed 3.

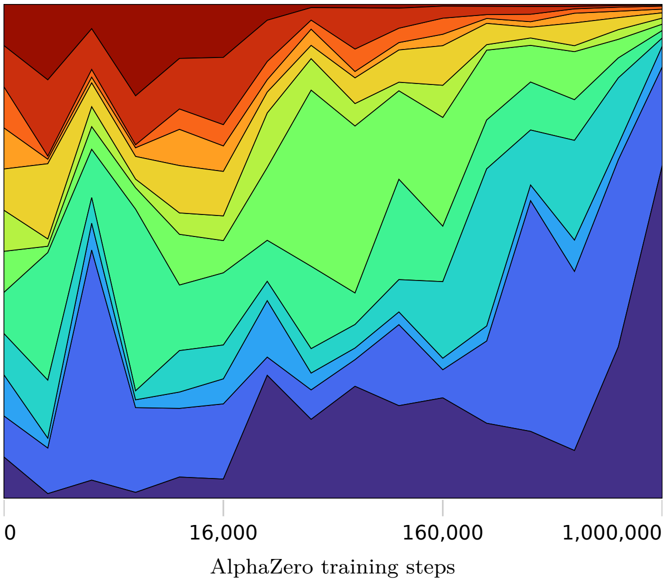

(f) Training seed 4.

Fig. S17. Top 12 moves with the highest probability after 1. e4 c6 2. d4 d5 3. e5 ♗f5.

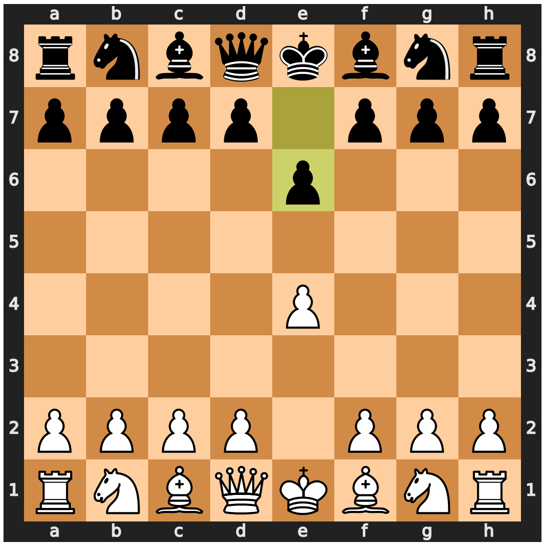

(a) Sequence start position.

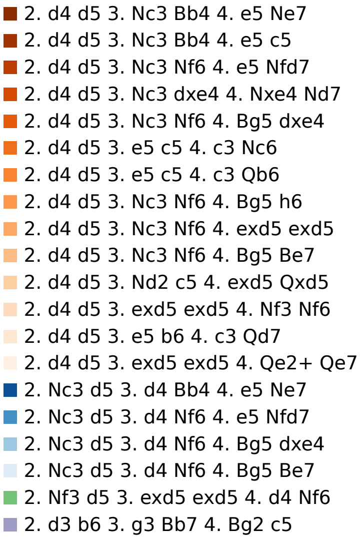

(b) Legend.

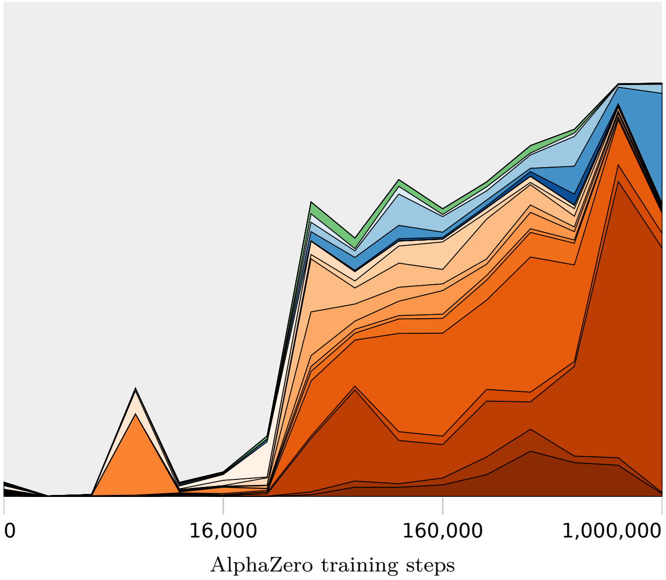

(c) Training seed 1.

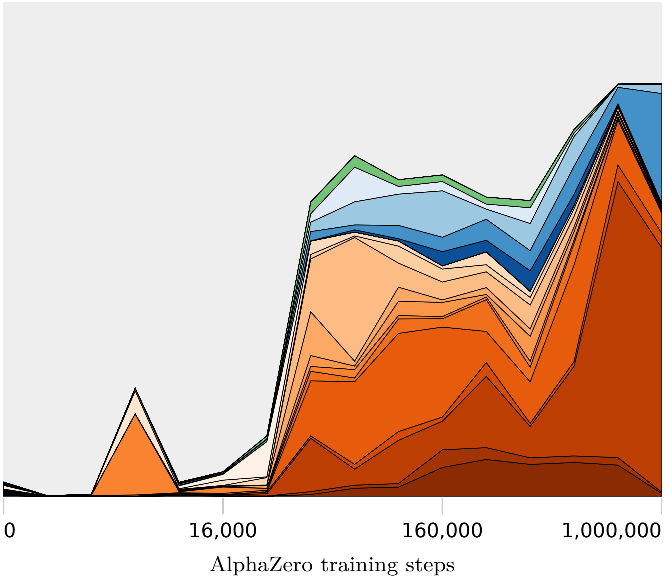

(d) Training seed 2.

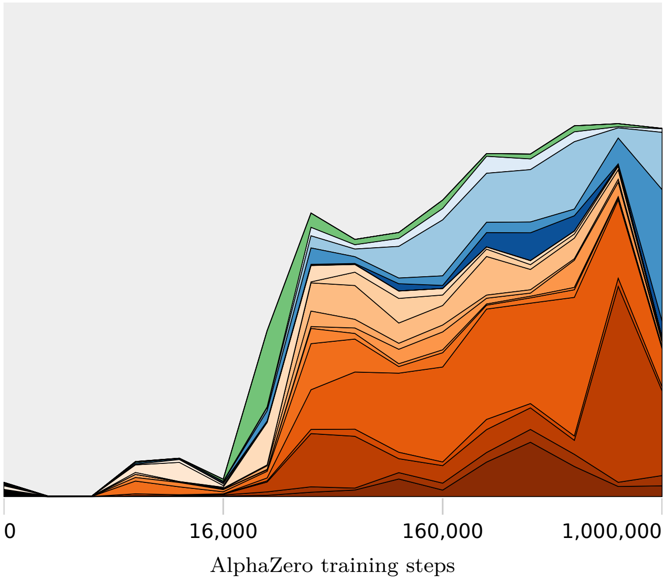

(e) Training seed 3.

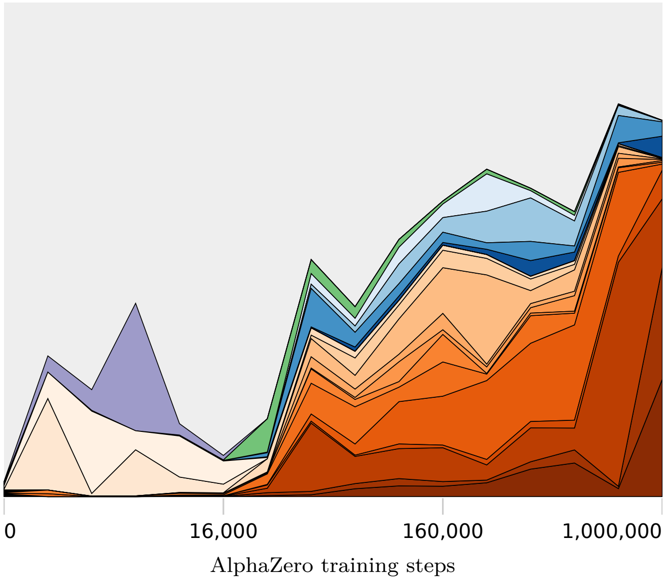

(f) Training seed 4.

Fig. S18. Top 20 move sequences with the highest joint probability after 1. e4 e6.

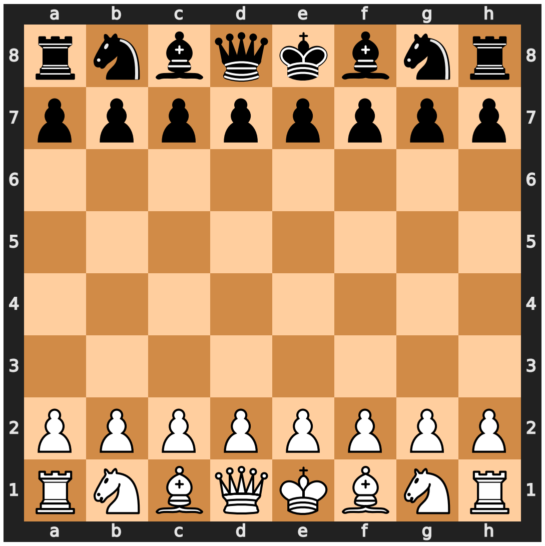

(a) Sequence start position.

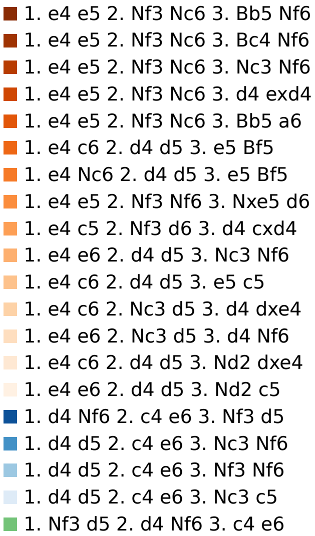

(b) Legend.

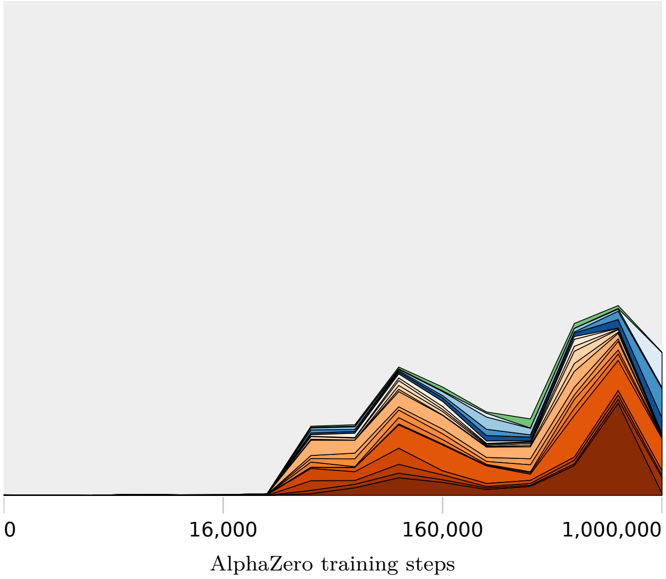

(c) Training seed 1.

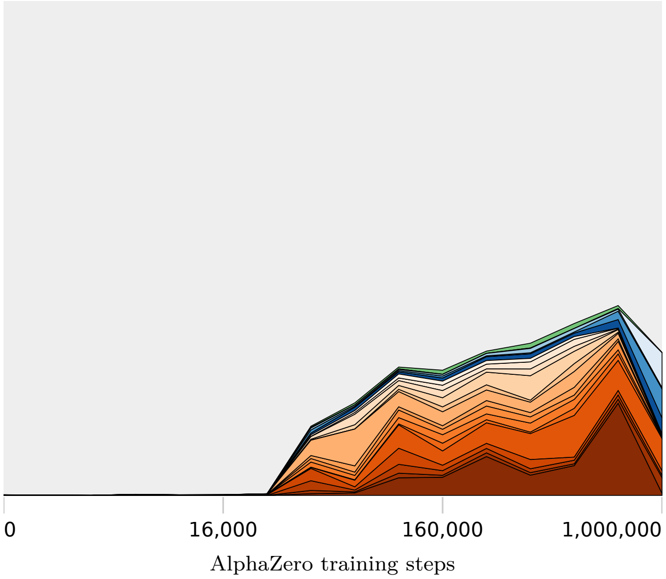

(d) Training seed 2.

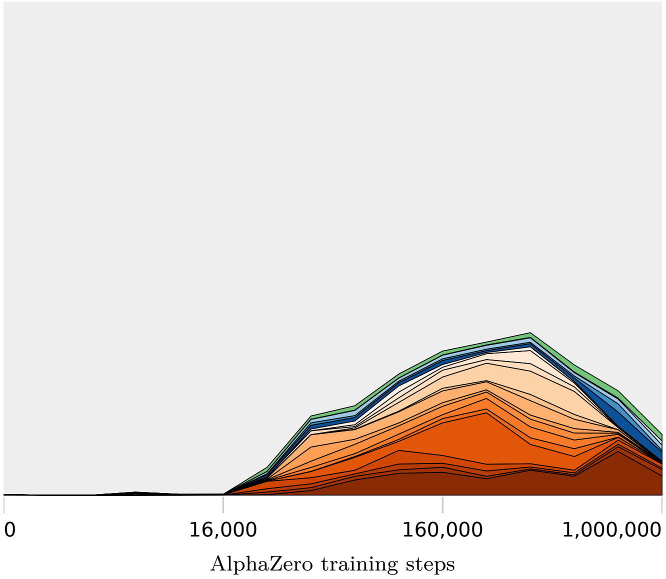

(e) Training seed 3.

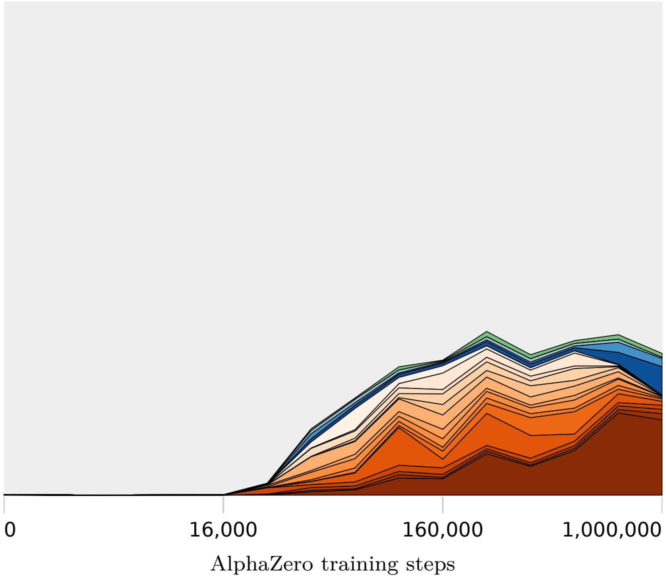

(f) Training seed 4.

Fig. S19. Top 20 move sequences with the highest joint probability at the start position.

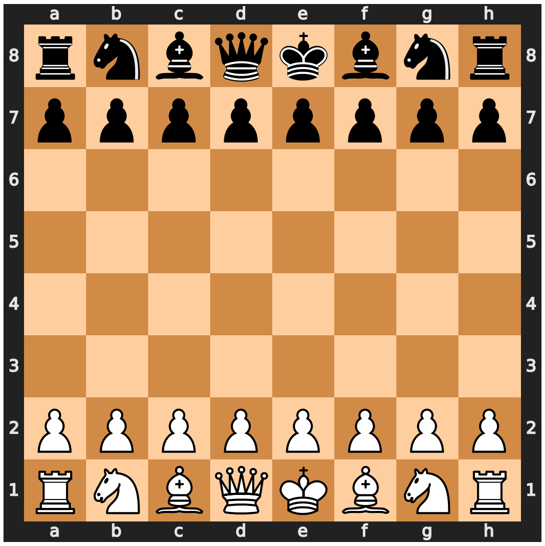

(a) Sequence start position.

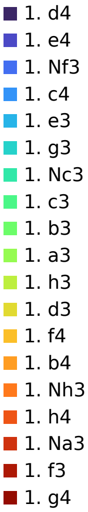

(b) Legend.

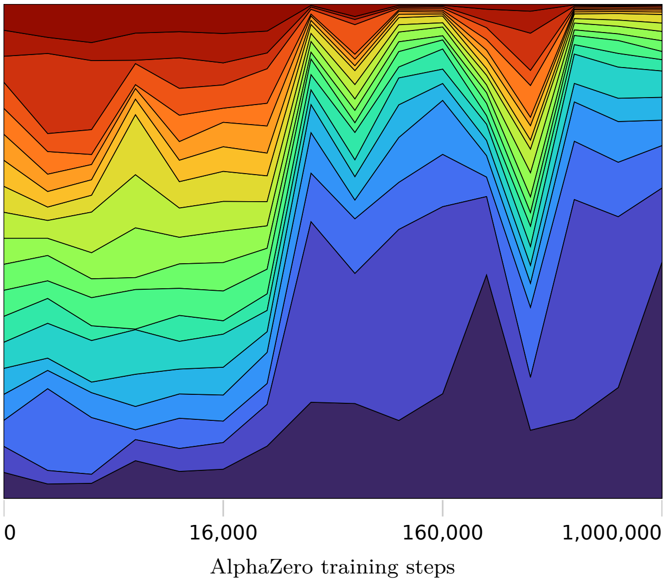

(c) Training seed 1.

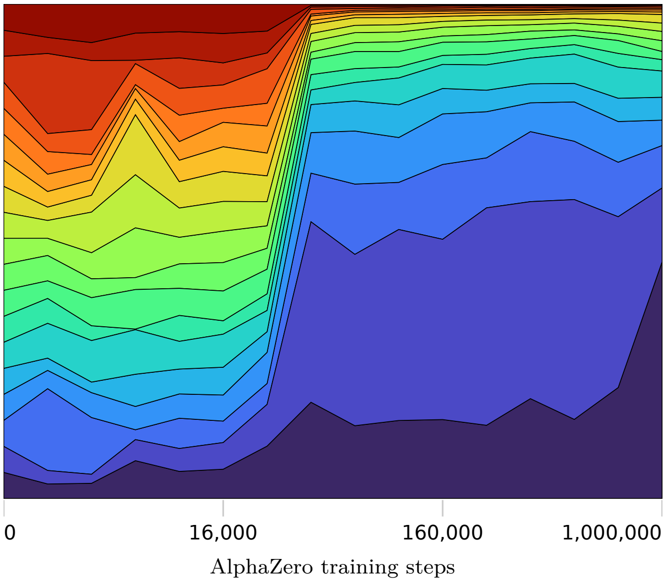

(d) Training seed 2.

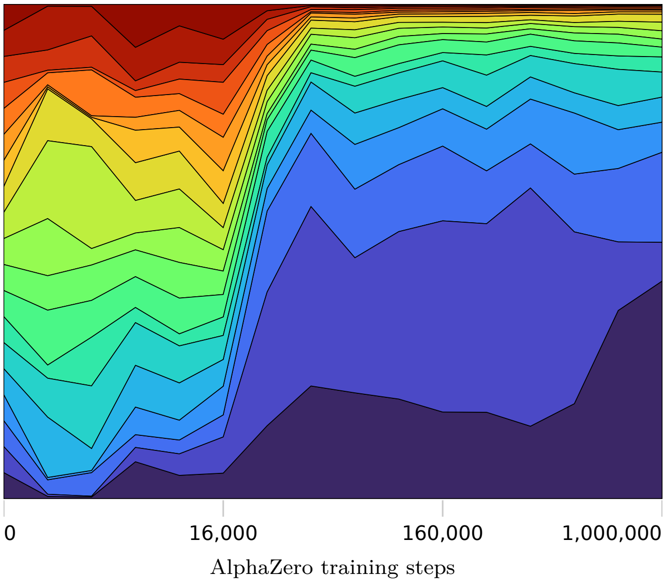

(e) Training seed 3.

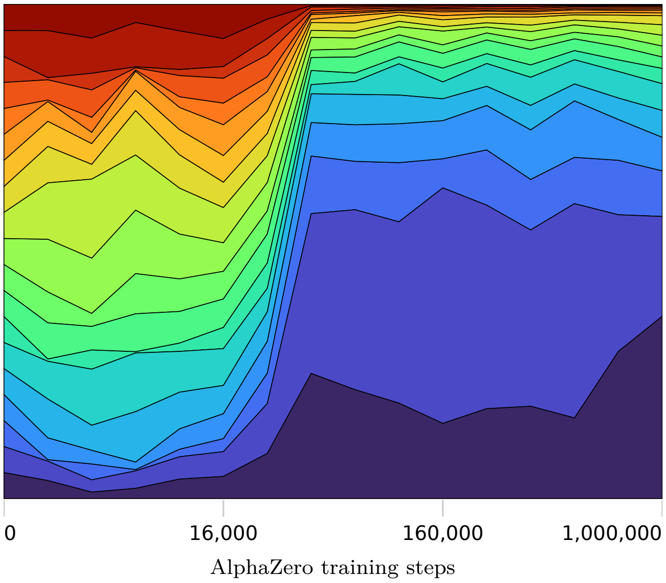

(f) Training seed 4.

Fig. S20. Top 19 moves with the highest probability at the start position.

5. Outlier positions in score regression

This section shows all the negative outlier positions shown in Figure ?? (those highlighted in red with true value less than zero). Black's queen can be captured in every position.

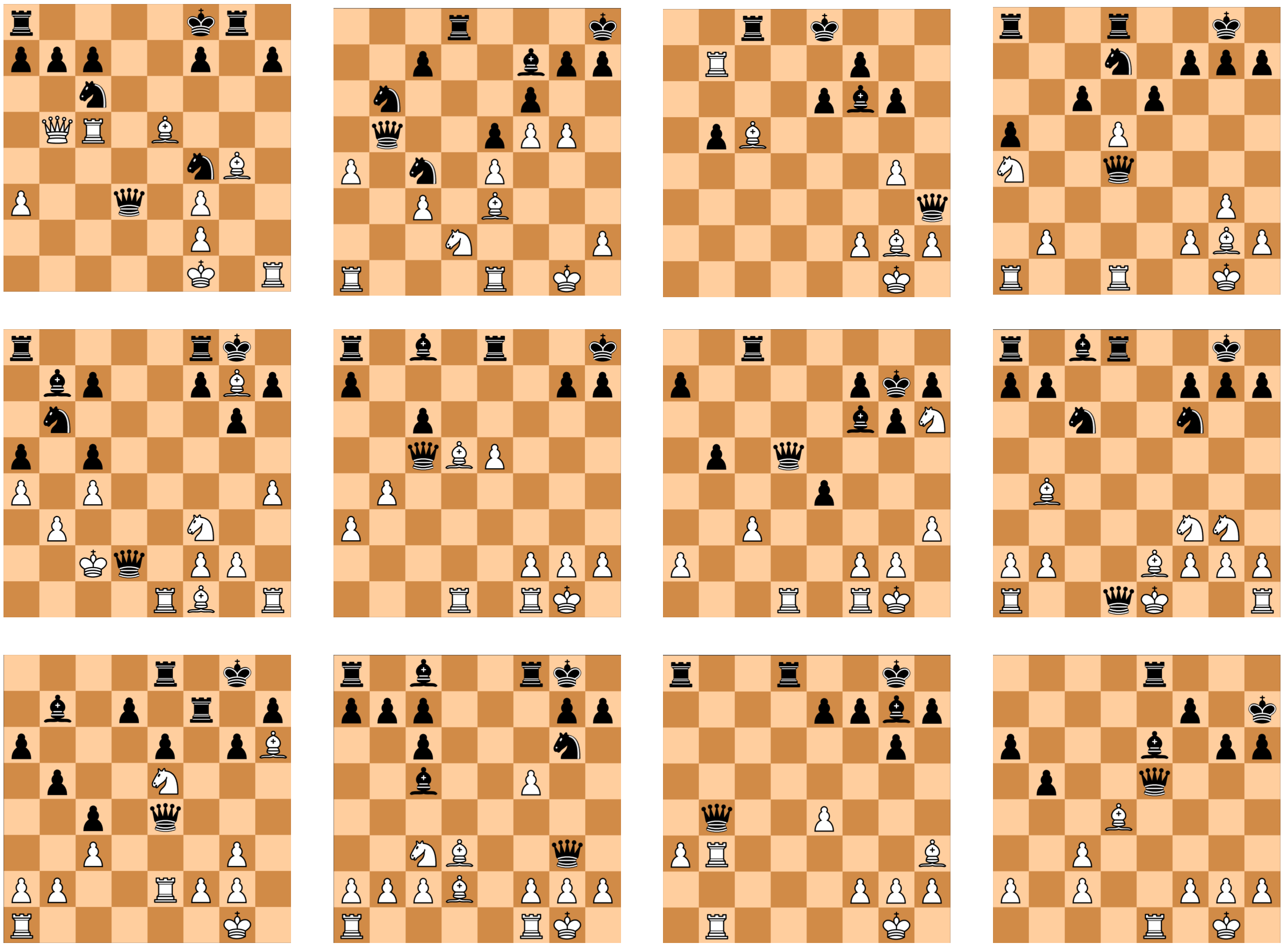

Fig. S21. All negative outliers shown in Figure 3 in the main text. Black's queen can be taken in every position.

6. Concept regression results for second seed

In order to ensure that our results for concept regression generalise beyond a single training run, we applied our concept regression methodology to a second trainingrun of AlphaZero. Our initial analysis was conducted on seed 3, and the following regression plots are obtained from seed 1. They plots match those in Figures S2 to S13 in Supplementary Information 3.

A. Regression results for Stockfish concepts from Table S1 (second seed).

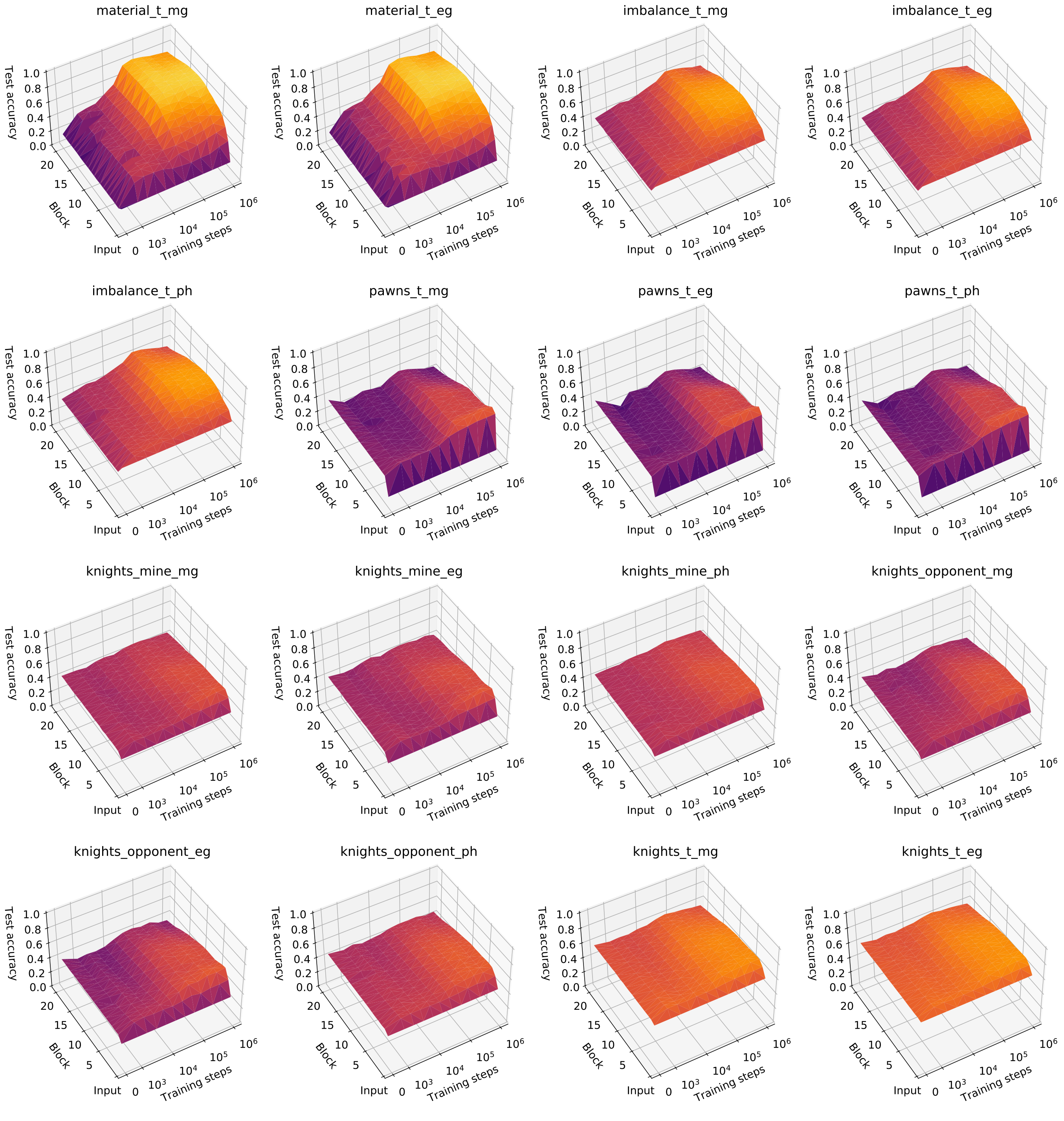

Fig. S22. Regression results for Stockfish concepts from Table S1.

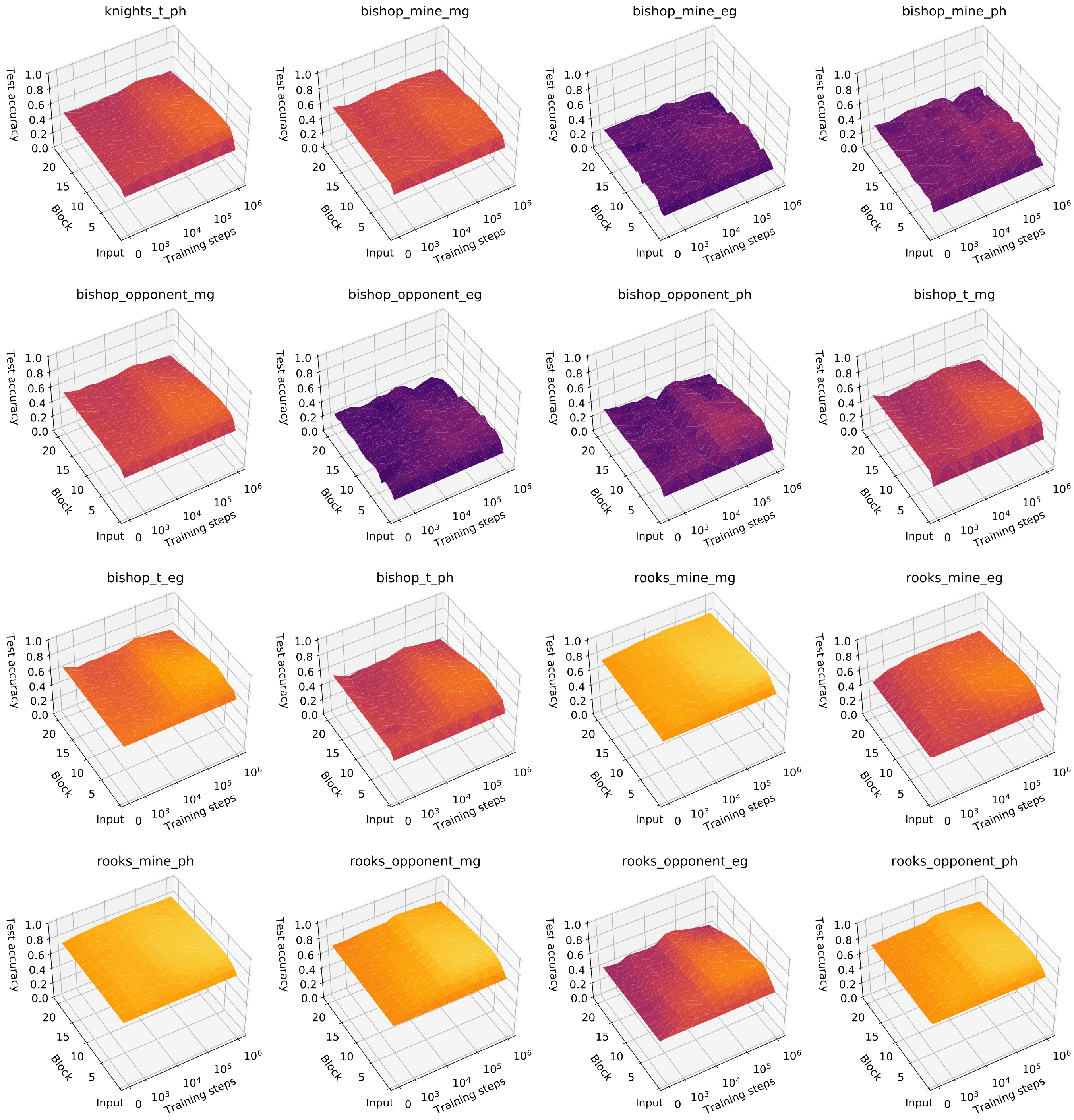

Fig. S23. Regression results for Stockfish concepts from Table S1, continued.

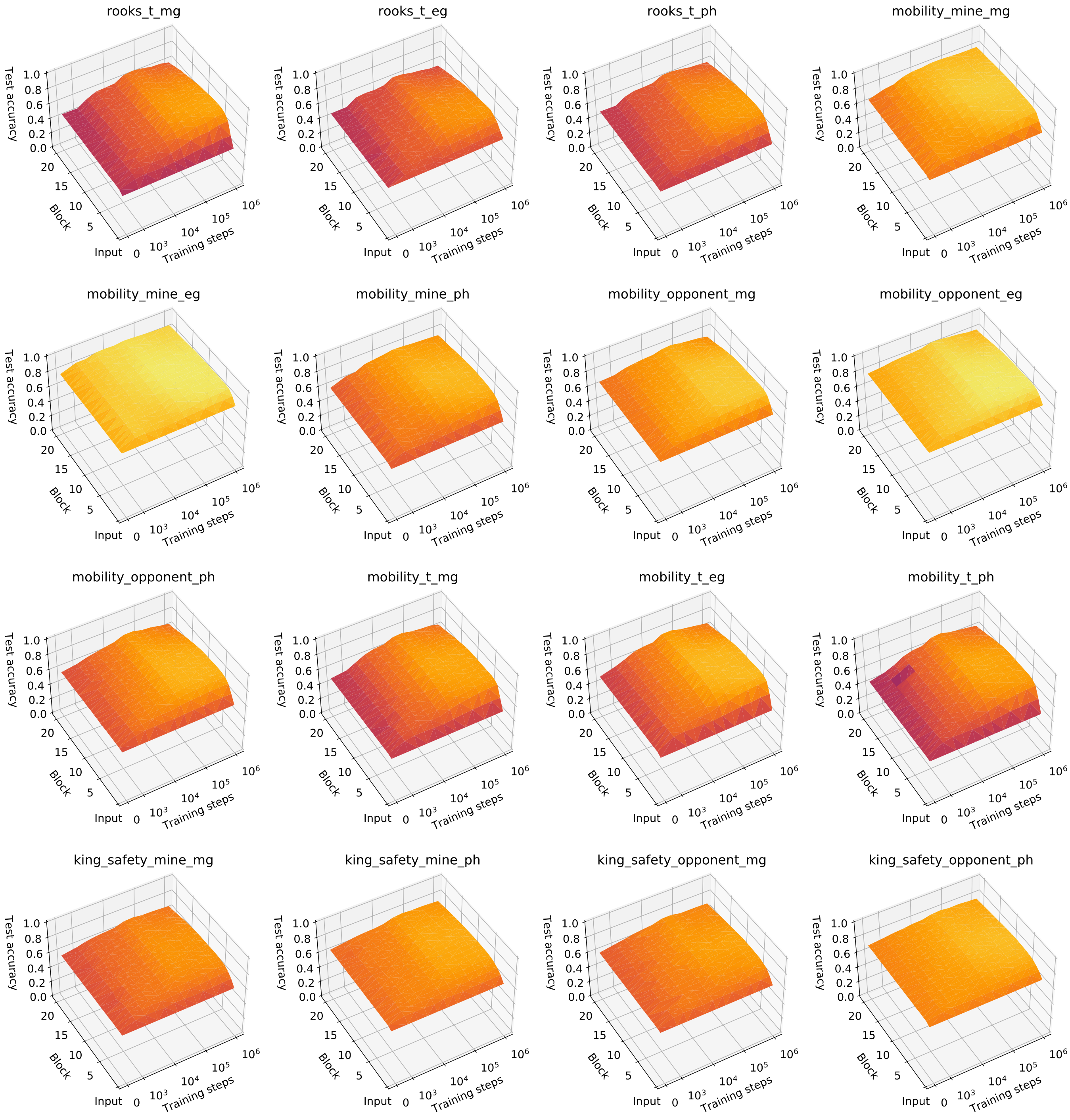

Fig. S24. Regression results for Stockfish concepts from Table S1, continued.

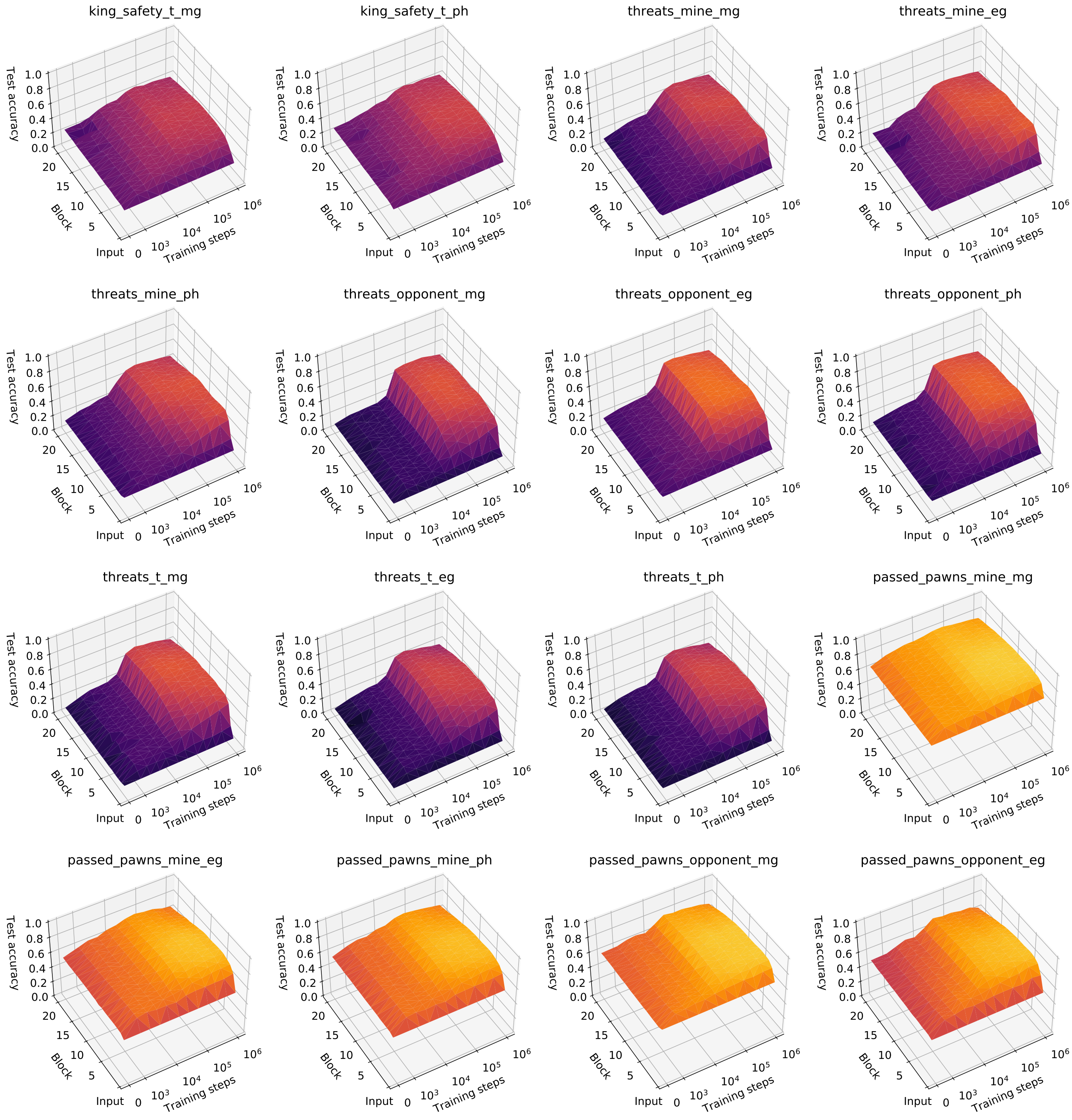

Fig. S25. Regression results for Stockfish concepts from Table S1, continued.

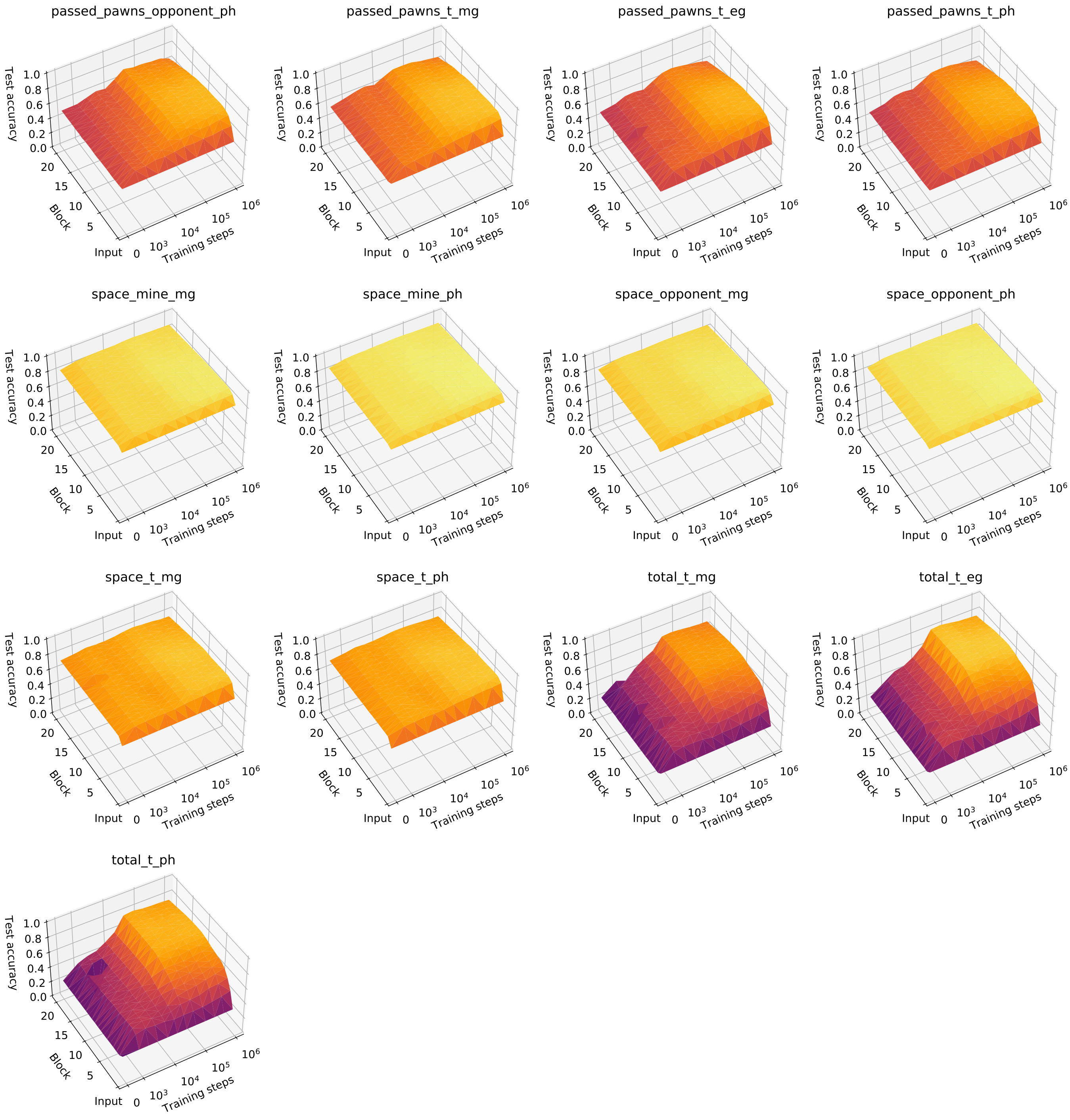

Fig. S26. Regression results for Stockfish concepts from Table S1, continued.

B. Regression results for custom concepts from Table S2, excluding capture-related concepts (second seed).

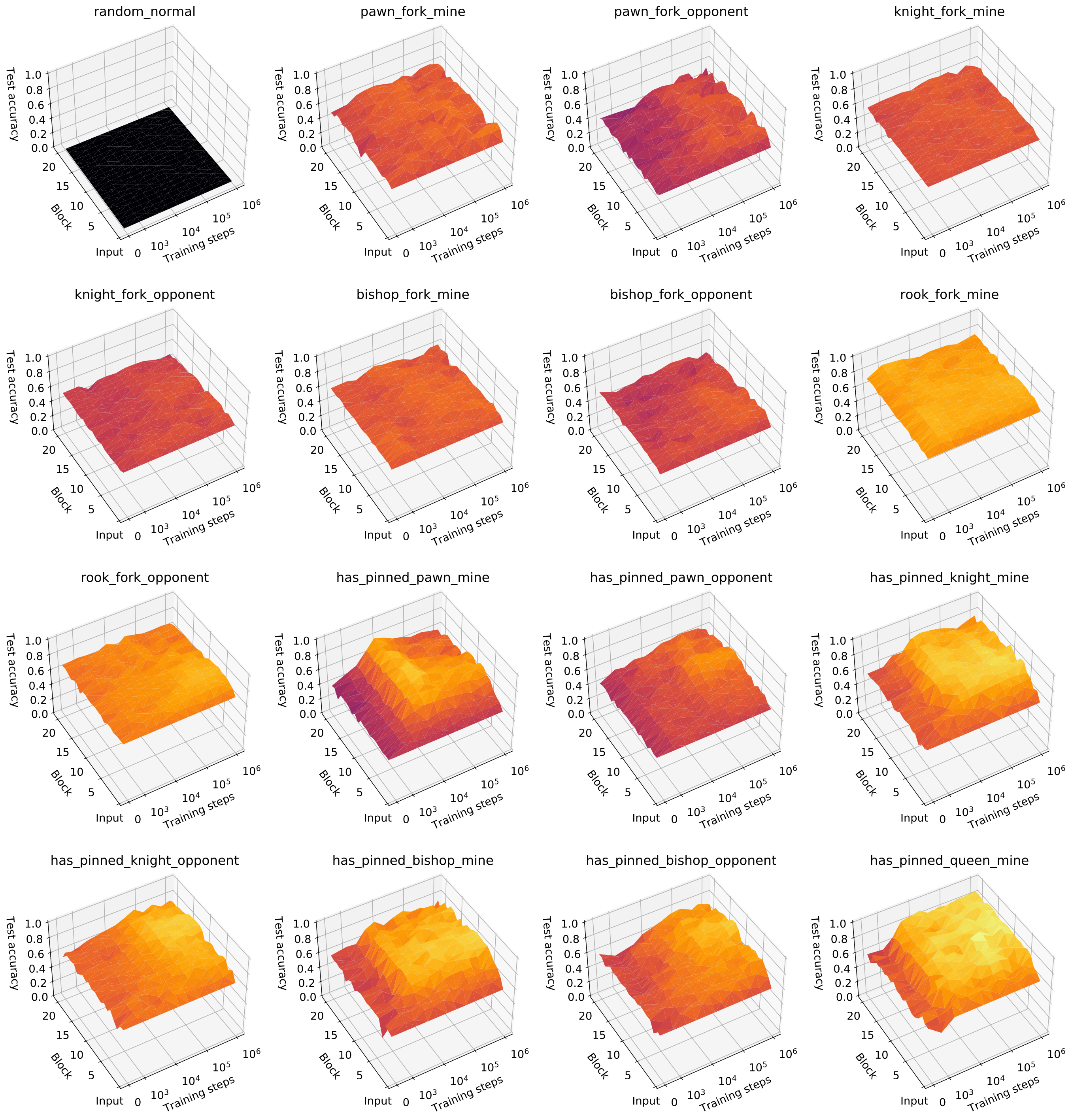

Fig. S27. Regression results for custom concepts from Table S2, excluding capture-related concepts. `random_normal` refers to random Gaussian labels with zero mean and variance of one.

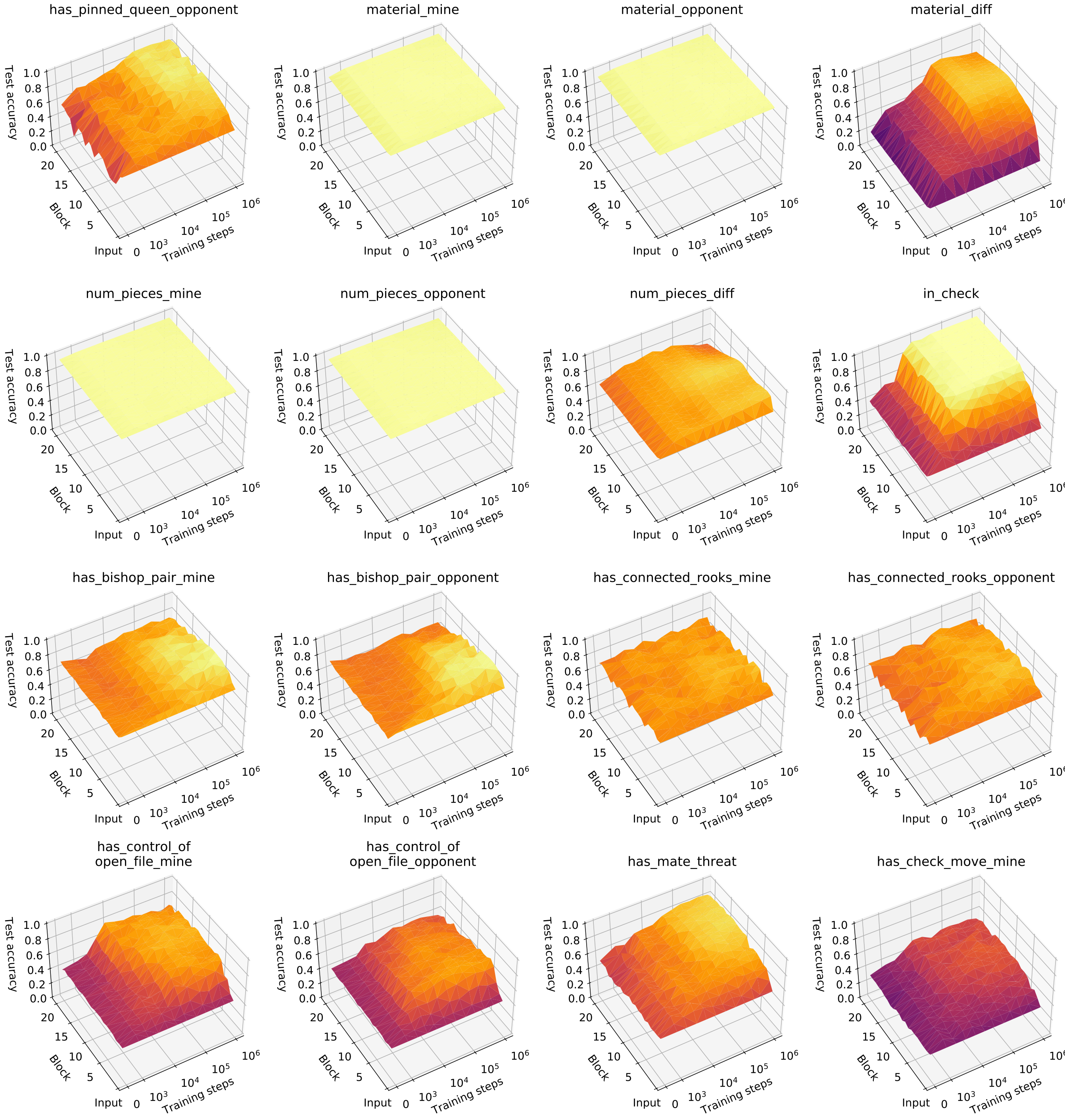

Fig. S28. Regression results for custom concepts from Table S2, excluding capture-related concepts, continued.

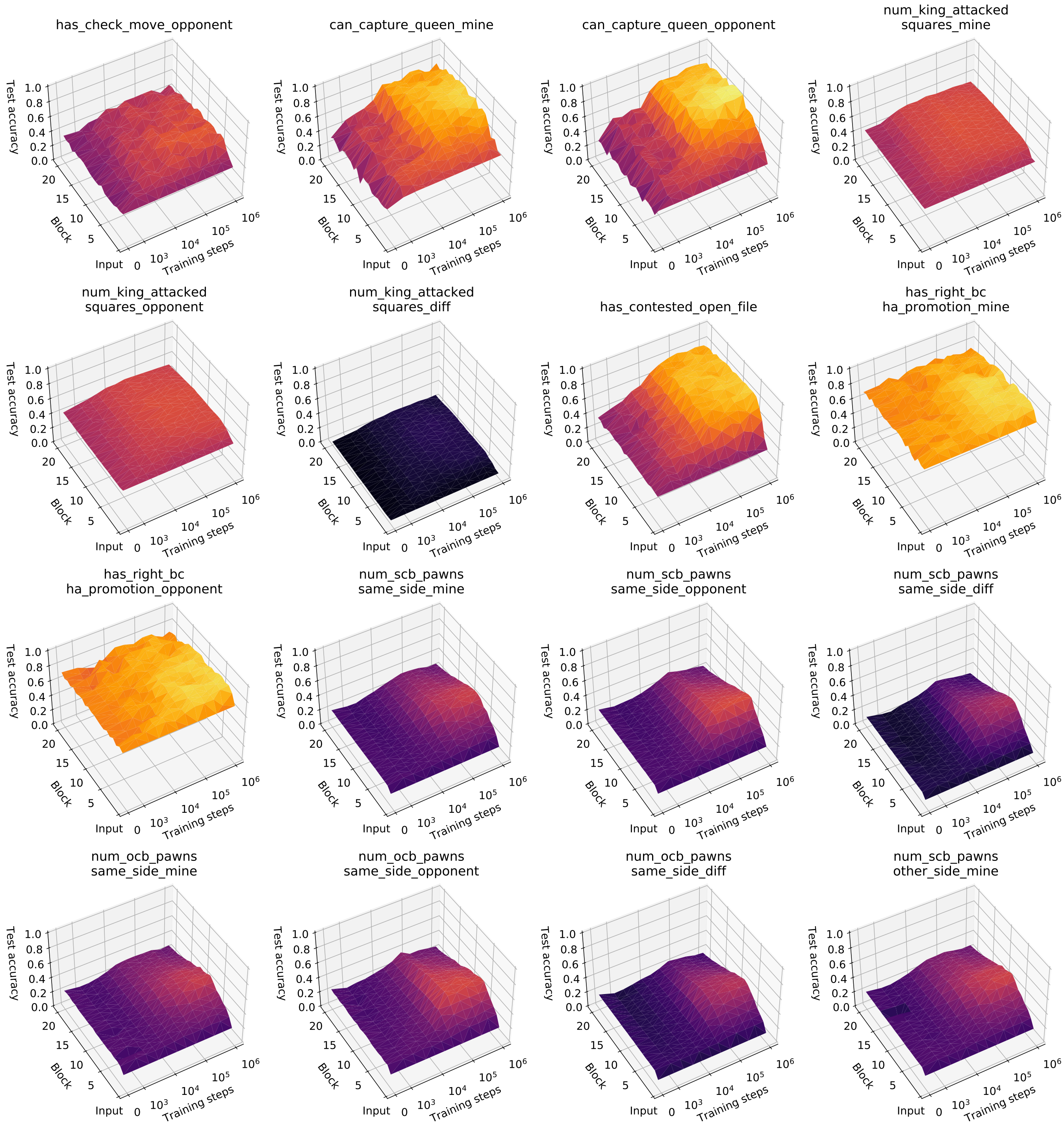

Fig. S29. Regression results for custom concepts from Table S2, excluding capture-related concepts, continued.

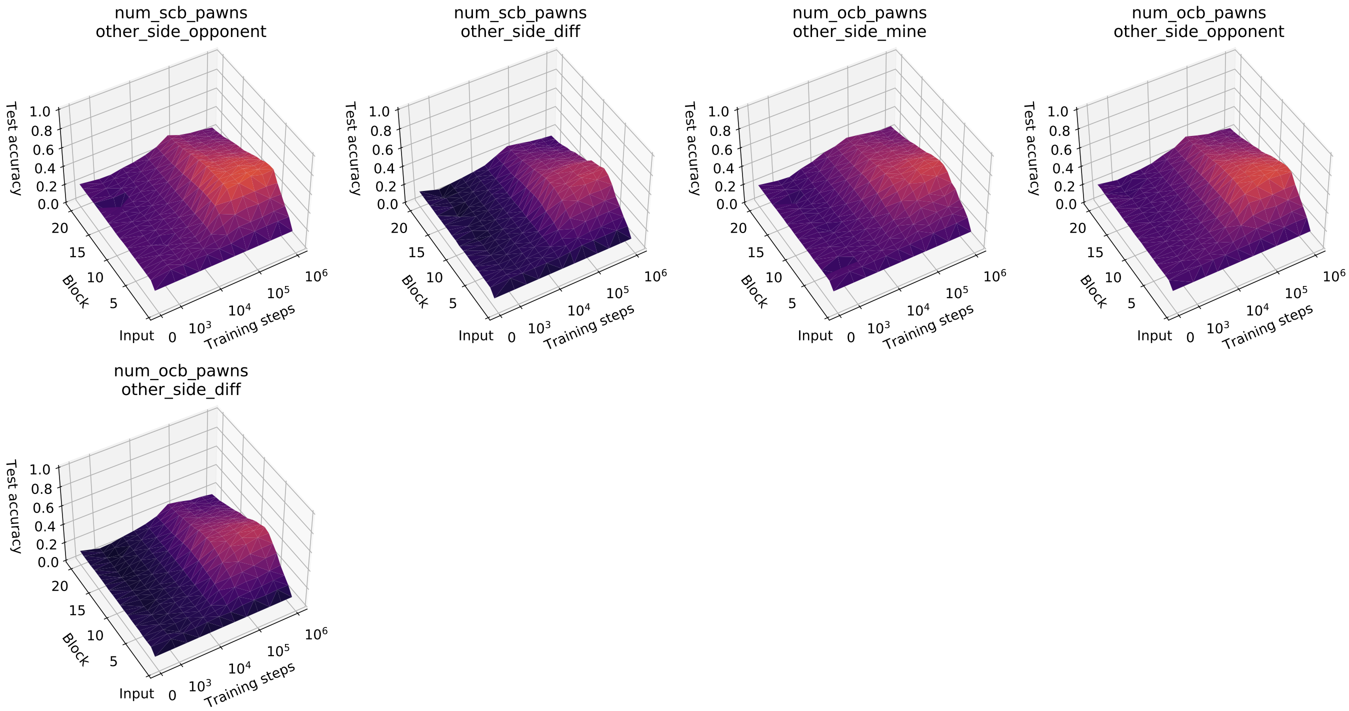

Fig. S30. Regression results for custom concepts from Table S2, excluding capture-related concepts, continued.

C. Regression results for custom concepts from Table S2 related to captures (second seed).

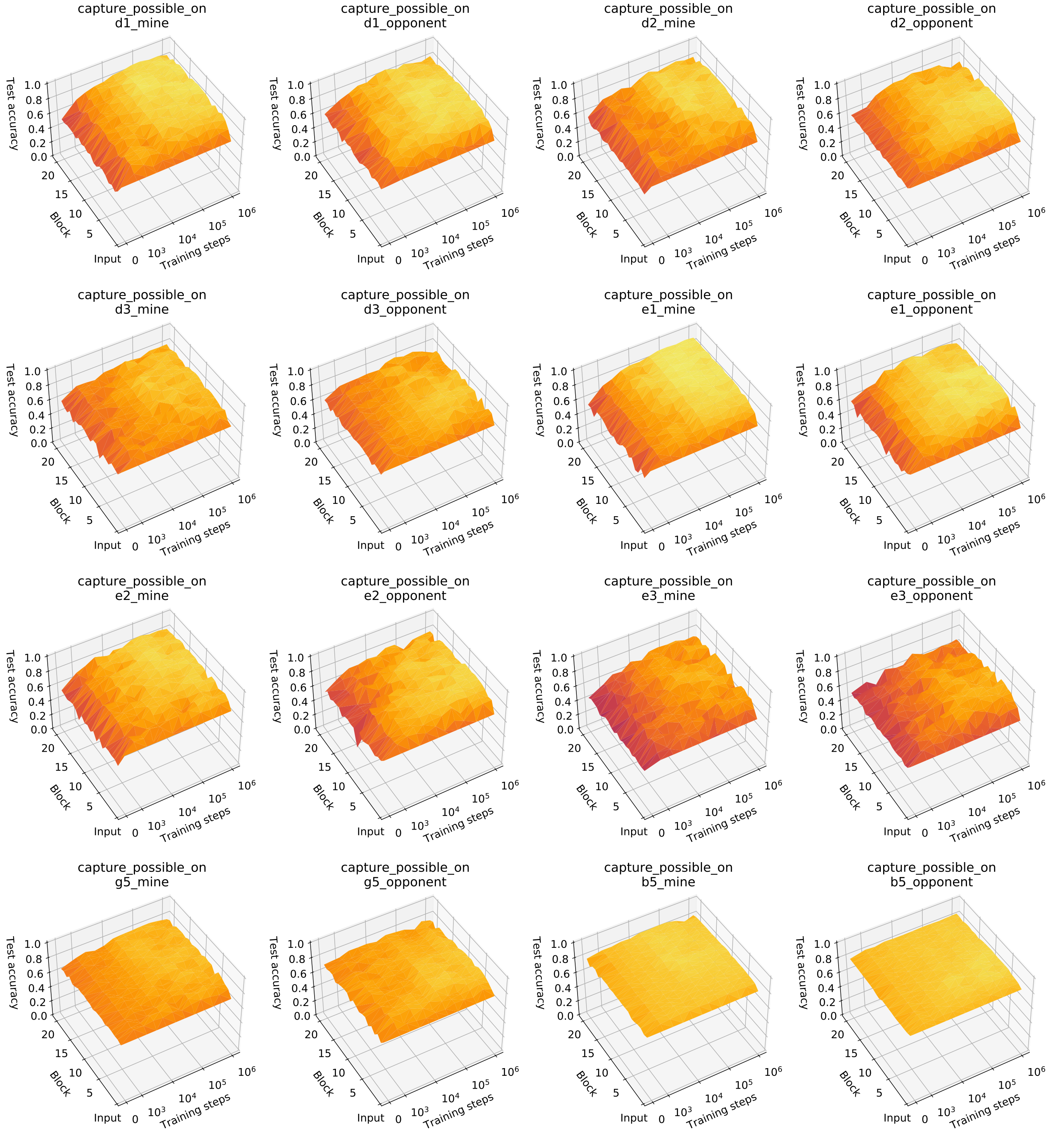

Fig. S31. Regression results for custom concepts from Table S2 related to captures.

D. Regression results for custom pawn-related concepts from Table S3 (second seed).

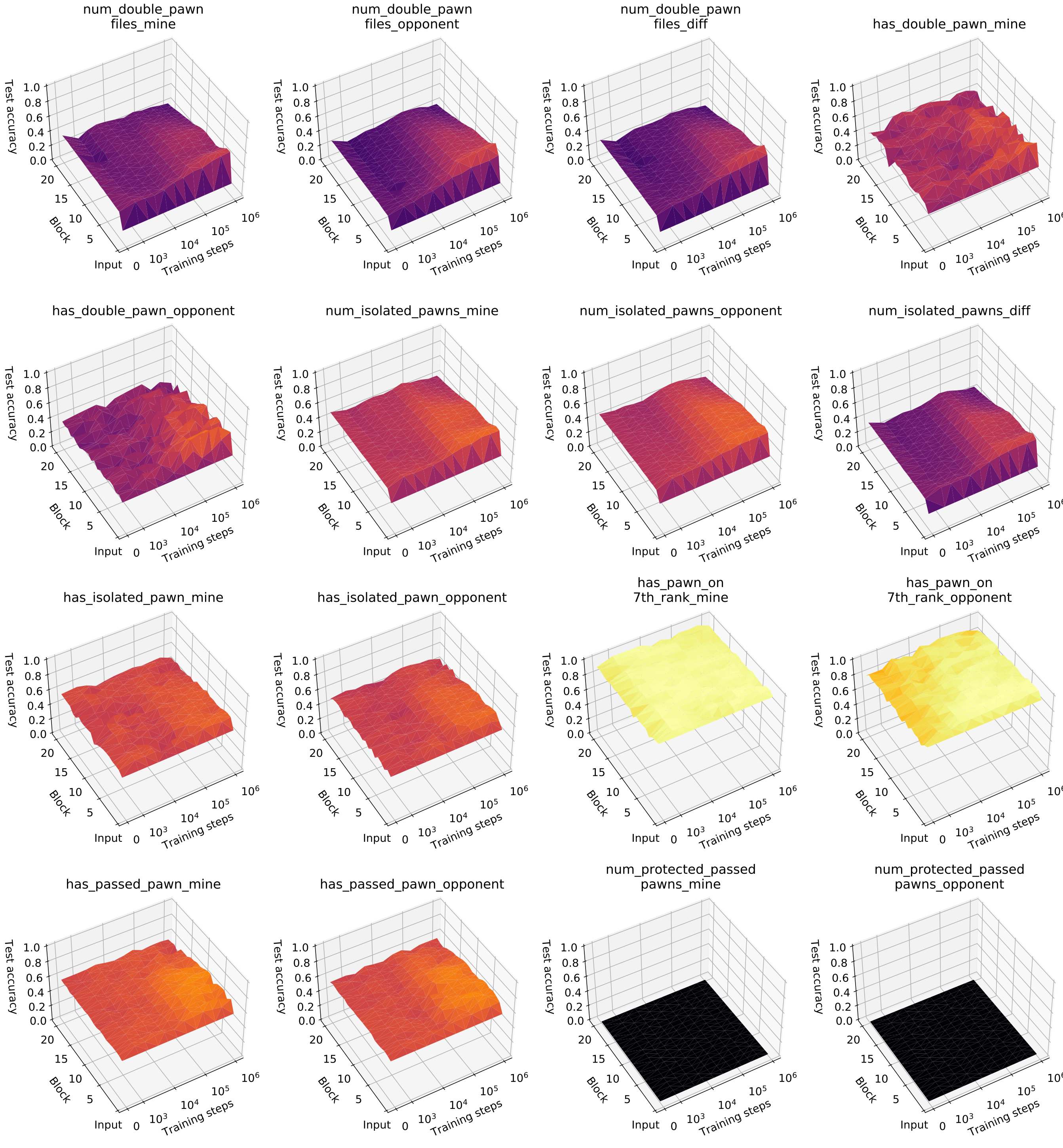

Fig. S32. Regression results for custom pawn-related concepts from Table S3.

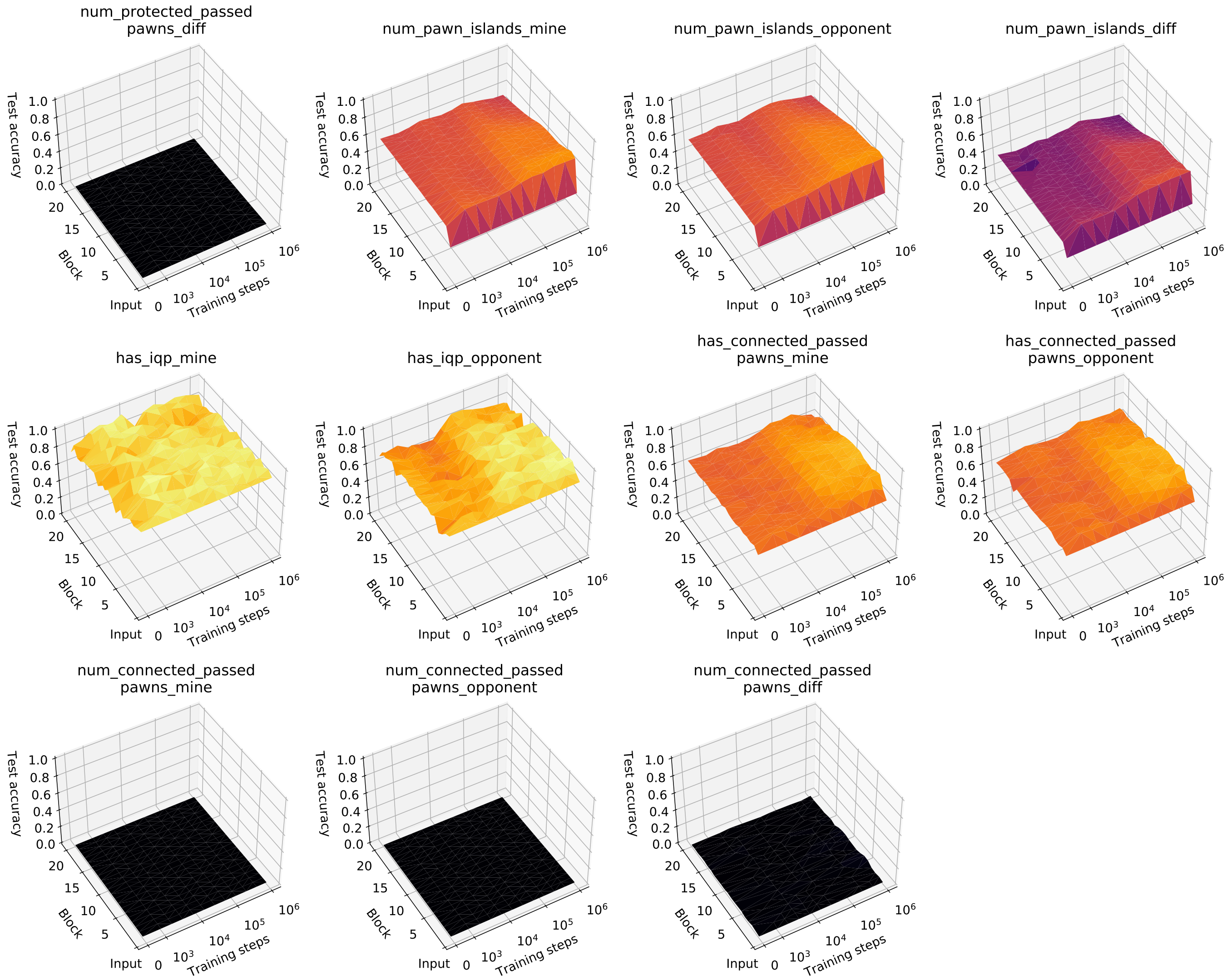

Fig. S33. Regression results for custom pawn-related concepts from Table S3, continued.

7. Exploring activations with unsupervised methods

While we investigated when and where the AlphaZero network encodes human conceptual knowledge, the approach was confined to labeled data of different human concepts, and was made possible through supervised methods. The public sub-functions of Stockfish's evaluation function and a large collection of custom chess concept functions allow the automation of large supervised data sets. Our approach so far has relied on supervised learning, either to regress predefined concepts from the network or to predict the value function from those concepts. This runs the risk of overlooking anything we have not included in our concept set, as well as biases our understanding of the AlphaZero network towards those concepts. In this section we take an alternative approach, using simple unsupervised approaches involving matrix decomposition (Section A). These methods generate a wealth of data to analyse at each layer of the network.

A. Non-negative matrix factorisation. Non-negative matrix factorisation (NMF) (6) is an approach to discover features in an unsupervised way. It has previously been used to interpret vision (7) and simple RL models (8). In this section we explore NMF as a complementary unsupervised approach to the supervised linear probes: rather than probe activations for specific concepts, we instead simplify the activations in a concept-agnostic way. This allows the structure of the activations to reveal itself, instead of imposing our assumptions on it.

A.1. Methodology. Layer d's activations $\mathbf{z}^d \in \mathbb{R}^{H \times W \times C} = \mathbb{R}^{8 \times 8 \times 256}$ are non-negative. Each of the 256 channels is an 8×8 plane. We treat each plane as a vector and reshape $\mathbf{z}^d$ into a matrix $\hat{\mathbf{z}}^d \in \mathbb{R}^{HW \times C}$. This is a square-by-channel matrix, where every row corresponds to a square and is a non-negative C-dimensional vector. We compress the activations $\mathbf{z}^d$ by representing each square as a non-negative K-dimensional weight vector, with $K < C$. It means the compression would be into a matrix $\mathbf{\Omega} \in \mathbb{R}^{HW \times K}$. In the reduced representation, the rows of $\mathbf{\Omega}$ correspond to squares, and its entries are K weights that sum together non-negative global factors $\mathbf{f}_k \in \mathbb{R}^C$ for $k = 1, \ldots, K$ so that the original row in $\hat{\mathbf{z}}^d$ is closely approximated. If the factor matrix $\mathbf{F} \in \mathbb{R}^{K \times C}$ contains the global factors as columns, the activations are approximated with $\hat{\mathbf{z}}^d \approx \mathbf{\Omega}\mathbf{F}$. For brevity we omit the dependence of the factor and weight matrices on the layer index d.

To determine the global factors $\mathbf{F}$, we stack the network activations of N randomly selected inputs $\hat{\mathbf{z}}^d_1, \ldots, \hat{\mathbf{z}}^d_N$ into a matrix $\hat{\mathbf{Z}}^d \in \mathbb{R}^{NHW \times C}$ (we use $N = 10^4$, and randomly select 50 positions to visualise). The factors and their weights $\mathbf{\Omega}_{\text{all}} \in \mathbb{R}^{NHW \times K}$ are found by minimizing

$$\begin{aligned} \mathbf{F}^*, \mathbf{\Omega}^*_{\text{all}} &= \min_{\mathbf{F}, \mathbf{\Omega}_{\text{all}}} \left\| \hat{\mathbf{Z}}^l - \mathbf{\Omega}_{\text{all}} \mathbf{F} \right\|_2^2 \\ \mathbf{F}, \mathbf{\Omega}_{\text{all}} &\geq \mathbf{0} \ . \end{aligned} \quad [6]$$

The stacked submatrices of $\mathbf{\Omega}^*_{\text{all}}$ correspond to the NMF weights for each input's activations. Alternatively, given $\mathbf{F}^*$, the NMF weights for any activations $\mathbf{z}^d$ could be retrieved by

$$\begin{aligned} \mathbf{\Omega}^* &= \min_{\mathbf{\Omega}} \left\| \hat{\mathbf{z}}^l - \mathbf{\Omega} \mathbf{F}^* \right\|_2^2 \\ \mathbf{\Omega} &\geq \mathbf{0} \ . \end{aligned} \quad [7]$$

To visualize the NMF factors for activations $\mathbf{z}^d$, we overlay the K columns of $\mathbf{\Omega}^*$ onto the input $\mathbf{z}^0$. The visualization of factor k's contributions to $\mathbf{z}^d$ is done by reshaping the column k of $\mathbf{\Omega}^*$ into a $H \times W$ or 8×8 matrix. The visualization shows how much NMF factor k contributes to each neuron's representation. This visualisation makes a strong assumption that representations in the residual block are spatially-aligned, i.e. an activation at a given spatial position can be interpreted in light of the pieces around that position. Although the network architecture biases towards this correspondence (all hidden layers are identical in shape to a chess board, and the residual network structure biases activations towards spatial correspondence) this is not strictly enforced by the architecture.

Using 36 factors per block, the full NMF dataset consists of 720 block/factor pairs. We report selected factors below, and the full dataset is available online. Most factors in later layers remain unexplained. We view explaining these factors (and developing the methods necessary to do so) as an important avenue for future work.

A.2. Results. This section highlights some illustrative examples of interpretable factors in AlphaZero's activations. The factors shown in Figure S34a and Figure S34b show the development of potential move computations for the player's and opponent's diagonal moves respectively. In the first layer moves of only three squares or fewer are shown (and only those towards the upper right of the board), demonstrating that move calculations take multiple blocks to complete. The convolutional structure of the AlphaZero network means that all computations from one layer to the next involve only spatially adjacent neurons. Because of this, move computations must occur over the course of multiple layers.

Figure S34c shows a more complex factor in layer 3: a count of the number of the opponent's pieces that can move to a given square (darker weights indicates more pieces can move to that square). This factor is likely to be useful in computing potential exchanges, and indicates that AlphaZero is also considering potential opponent moves even early in the network. Figure S34d appears to show potential moves for the current player - darker squares indicate better moves (for instance the opponent's hanging queen on d2).

The NMF factor data contains many factors we have not yet interpreted, especially in later layers of the network. Where factors are uninterpretable, this could be because the number of factors used is incorrect, spatial correspondence is broken, or

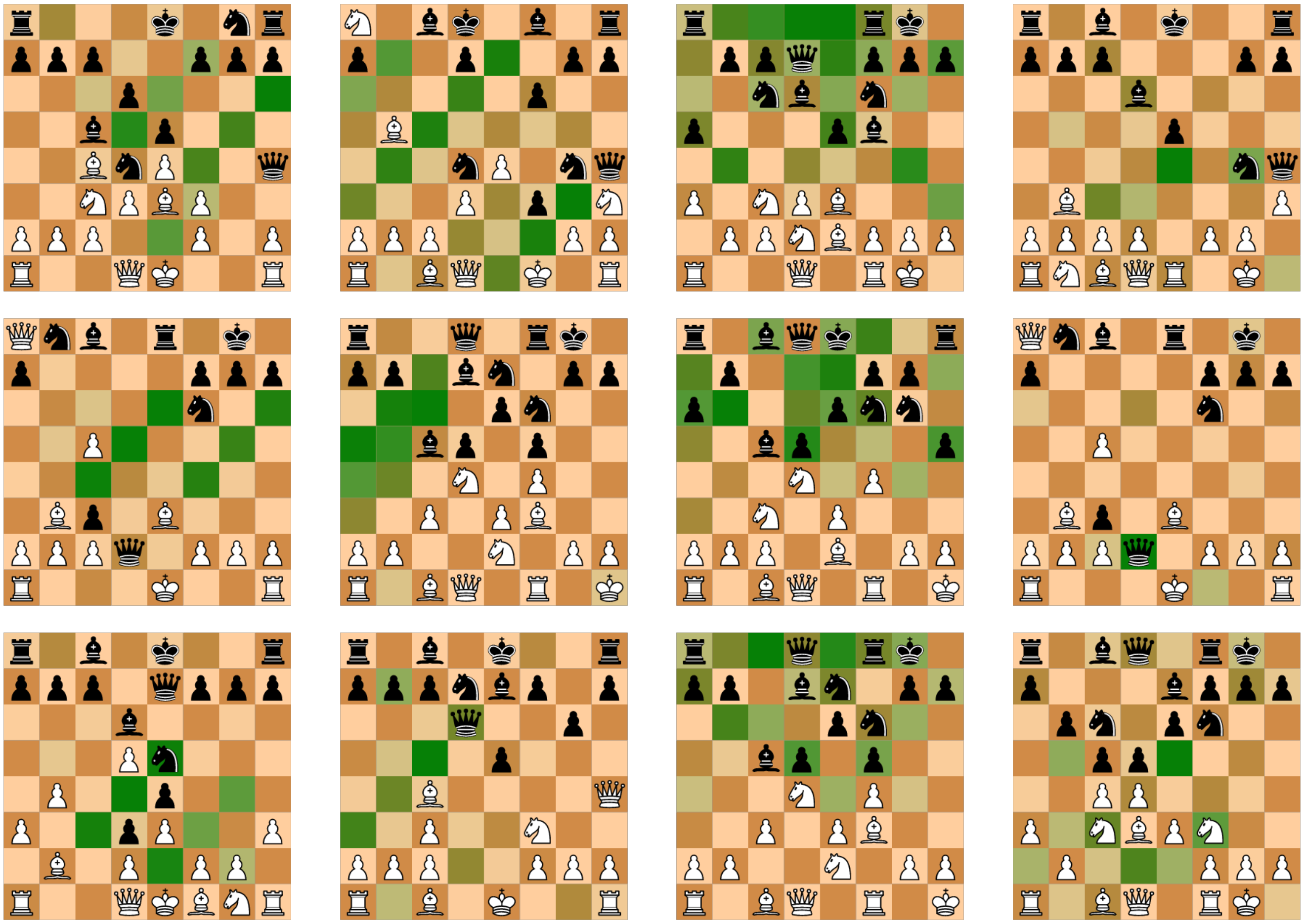

(a) Development of diagonal moves for player (block 1, factor 26 of 36).

(b) Fully developed diagonal moves for opponent (block 3, factor 22 of 36).

(c) Count of opponent's potential piece moves (block 3, factor 11 of 36).

(d) Potential good squares to move to? (block 18, factor 22 of 36).

Fig. S34. Visualisation of NMF factors in a fully-trained AlphaZero network, showing development of threats and anticipation of possible moves by the opponent, as well as a factor that may be involved in move selection. Following Figure S1, we count a ResNet 'block' as a layer.

the factor represents something too complex to understand without reference to earlier layers. Development of methods for relating complex later-layer factors to well-understood early layer factors is an important priority for further interpretability work in complex domains. Finally, we note that although these interpretations of the above factors bear out in the majority of the randomly selected positions shown in the online database of factors, an interpretation can only be considered definitive once it has been quantitatively validated (9), ideally by intervening on the input.

8. Comparison of results across regularisers

In order to test robustness to our choice of regularisers we computed test score accuracy metrics under three choices of regulariser:

1. an unregularised linear probe, trained solely on the classification loss $\mathcal{L}$,
2. a linear probe trained with L_1 regularisation, giving loss

$$\mathcal{L} + \lambda|\mathbf{w}| \tag{8}$$

   where λ is a hyperparameter chosen by cross-validation (see main text), and finally
3. a linear probe with group sparsity

$$\mathcal{L} + \beta \sum_{c \in \mathcal{C}} \|\mathbf{w}_c\|_2, \tag{9}$$

   where $\mathbf{w}_c$ is the set of weights associated with regression from a given channel.

We use $\beta = 0.01$. Group sparsity regularises the probe towards either learning from every element of a channel, or ignoring that channel entirely. This inductive bias intuitively matches the inductive bias associated with convolutional networks. In the subsequent figures we present what-when-where plots for each concept side-by-side in order to allow for easy comparison. The leftmost plot shows results from the unregularised probe, the centre plot shows the l_1-regularised probe, and the rightmost plot shows results from the group-sparse probe. Qualitatively the results are similar for almost all concepts, showing that our results are generally robust to our choice of regulariser (although regression scores are higher for both the unregularised and group-sparse probe, suggesting that our initial choice of L_1 regularisation may be suboptimal and group sparsity may be a superior regulariser for probing convolutional networks).

Interestingly, the threats concepts are not substantially changed by removing regularisation. This allows us to rule out the hypothesis that our comparatively low maximum regression accuracy for threats is due to requiring a higher-dimensional pattern than the L_1 probe allowed. Concepts relating to pawns (which are more numerous and thus likely to require more parameters to predict), as well as more complex relational concepts such as forks, pins, and contested/open files all benefitted disproportionately from group-sparse regularisation.

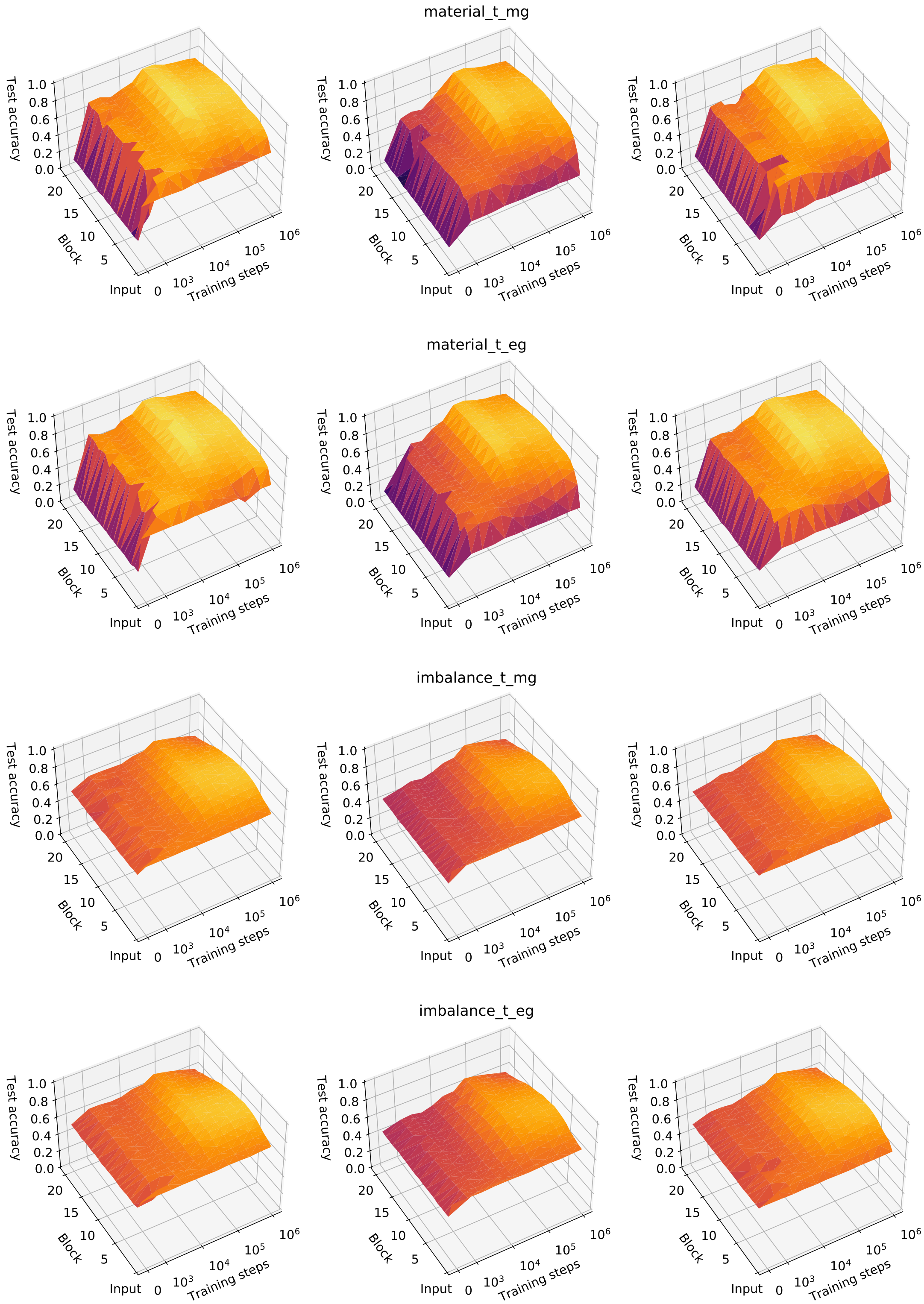

Fig. S35. Comparison of concept regression results between regularisers. Left: unregularised, centre: l_1 regularised, right: group-sparse.

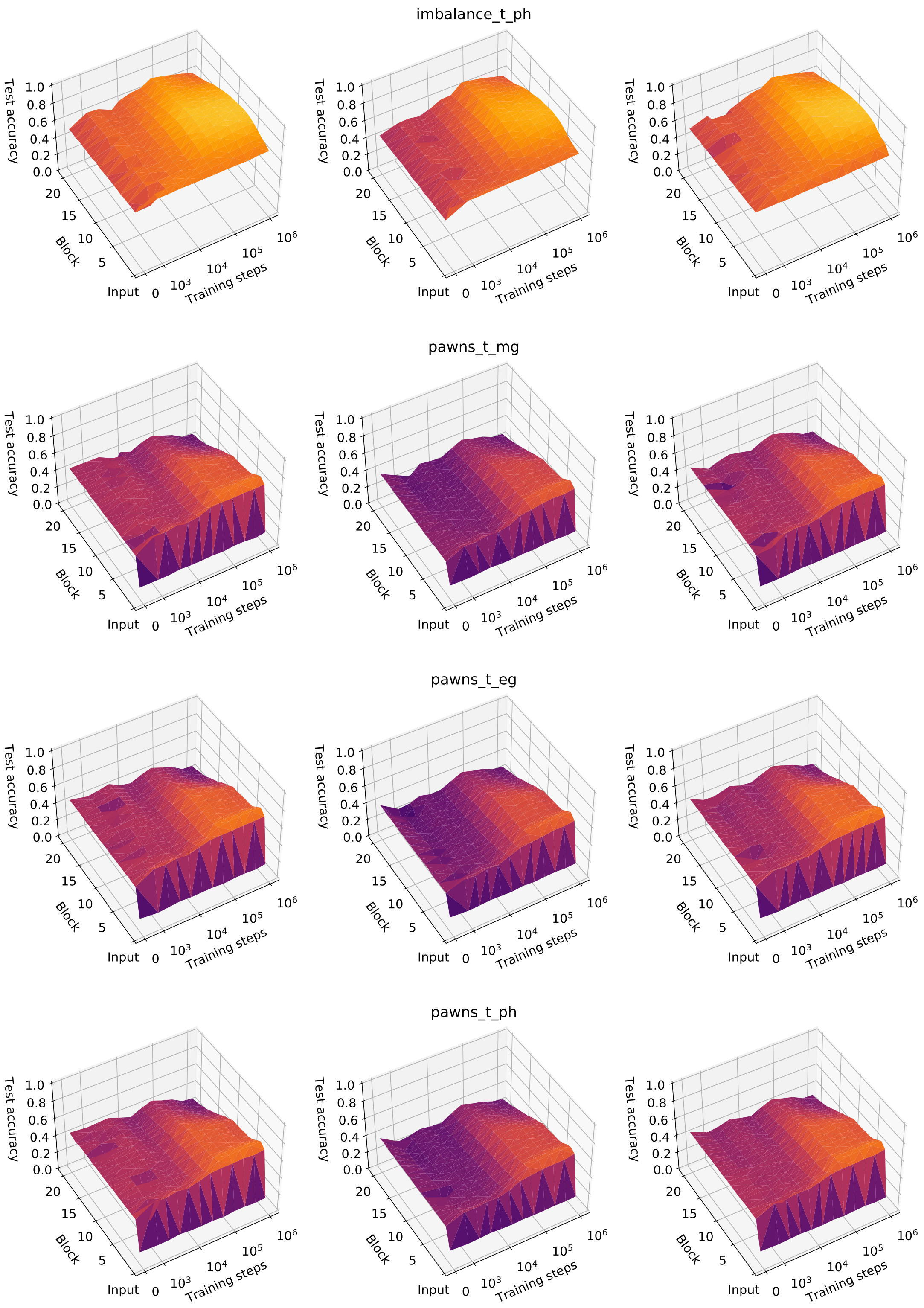

Fig. S36. Comparison of concept regression results between regularisers. Left: unregularised, centre: l_1 regularised, right: group-sparse.

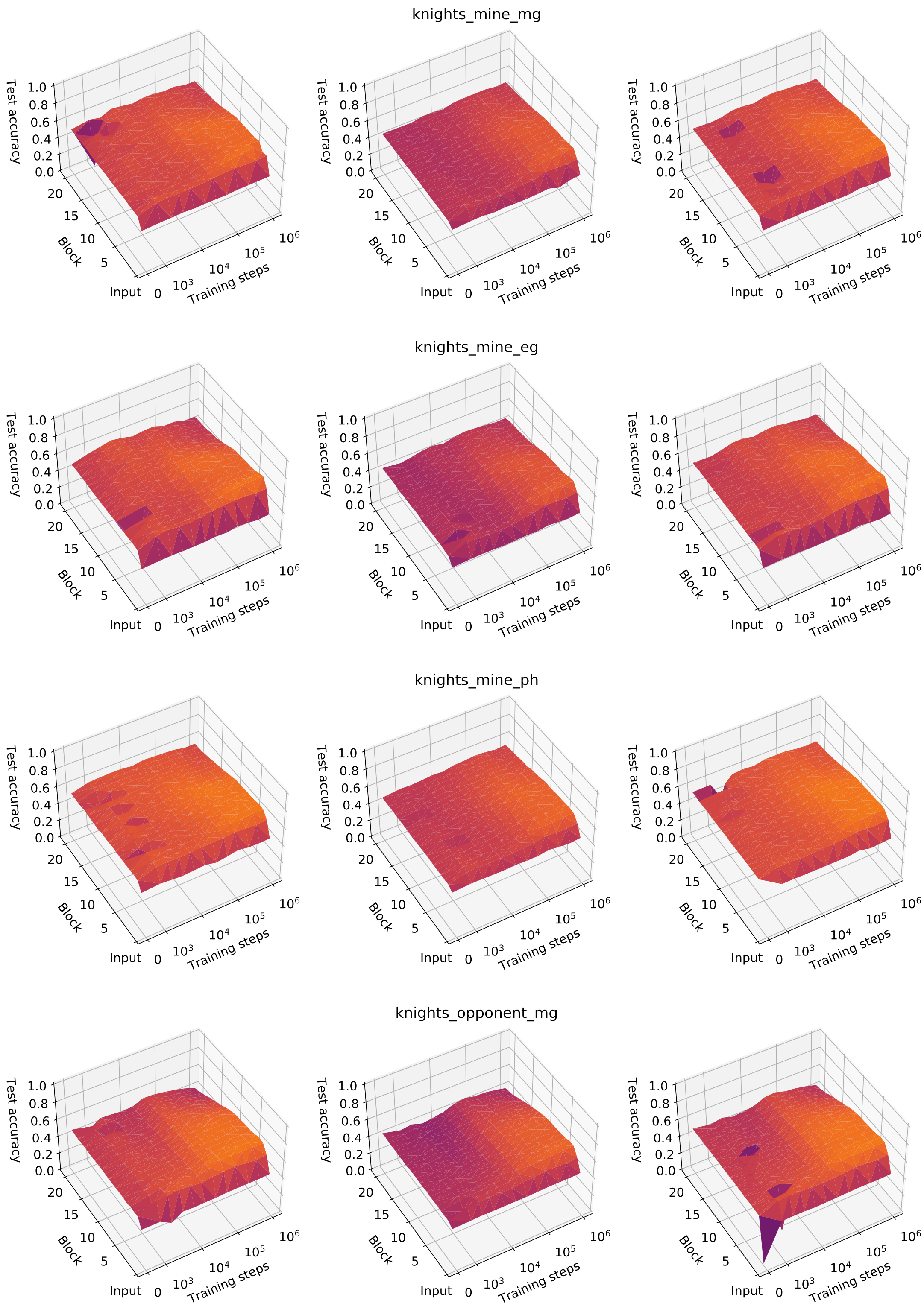

Fig. S37. Comparison of concept regression results between regularisers. Left: unregularised, centre: l_1 regularised, right: group-sparse.

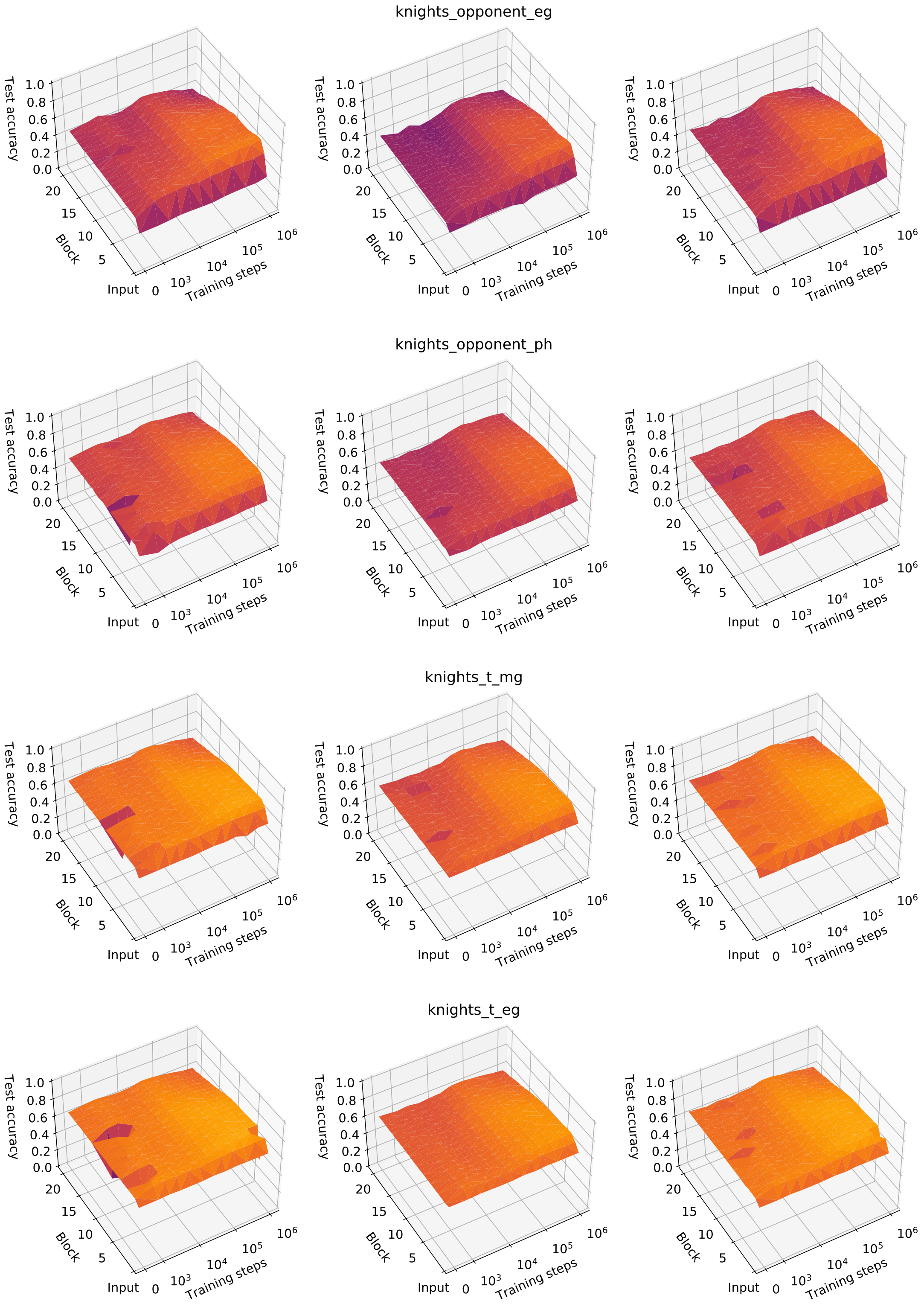

Fig. S38. Comparison of concept regression results between regularisers. Left: unregularised, centre: l_1 regularised, right: group-sparse.

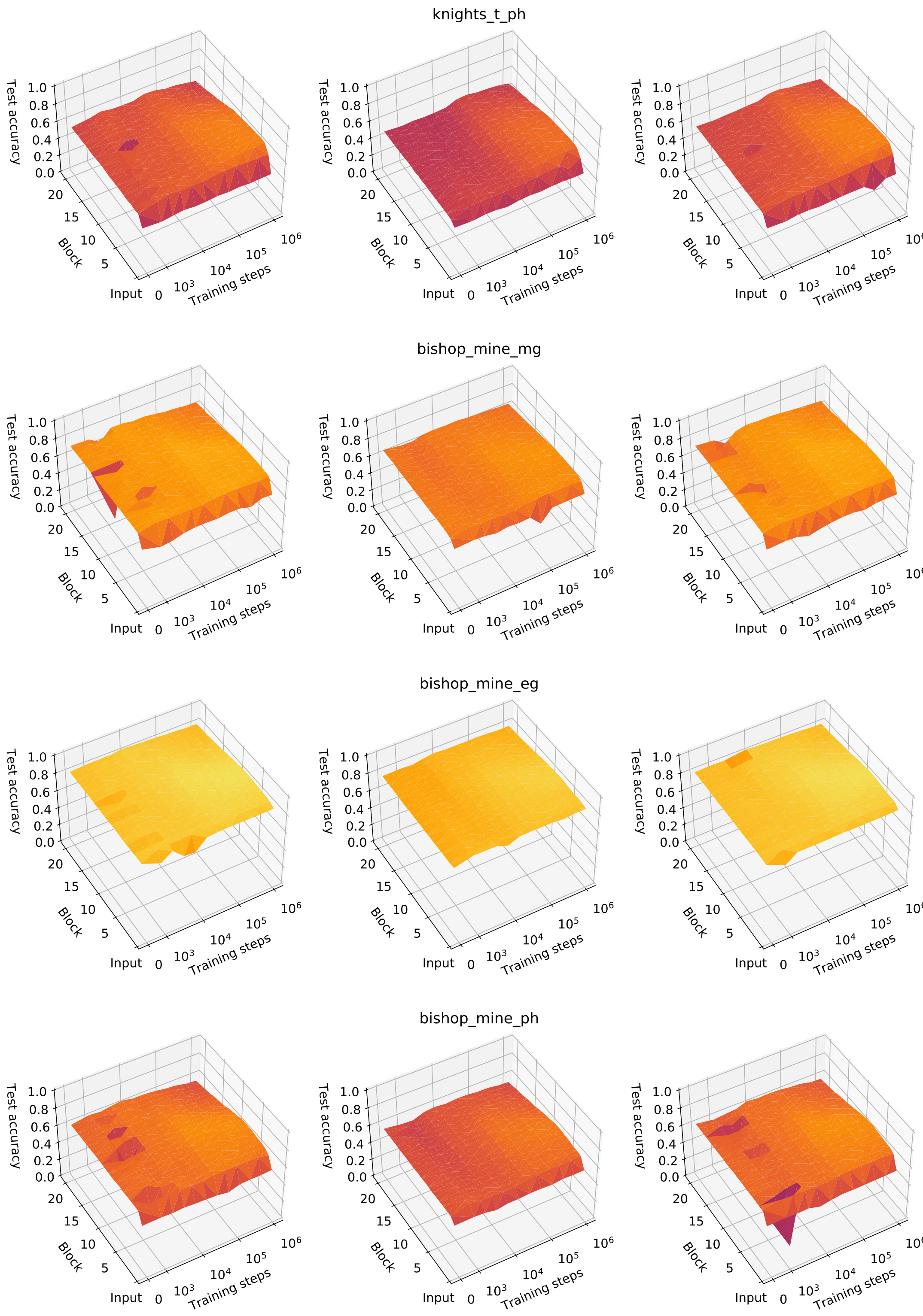

Fig. S39. Comparison of concept regression results between regularisers. Left: unregularised, centre: l_1 regularised, right: group-sparse.

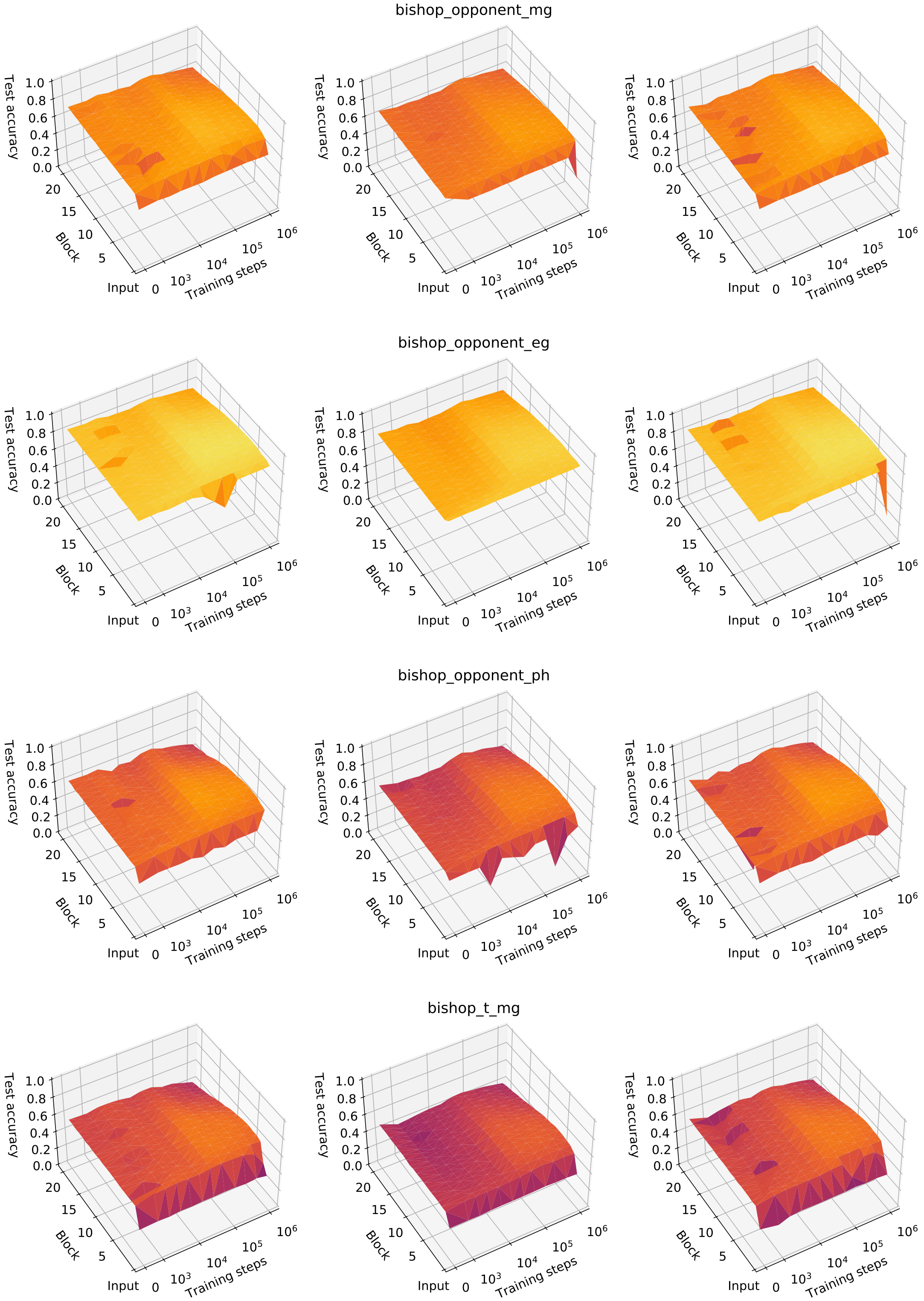

Fig. S40. Comparison of concept regression results between regularisers. Left: unregularised, centre: l_1 regularised, right: group-sparse.

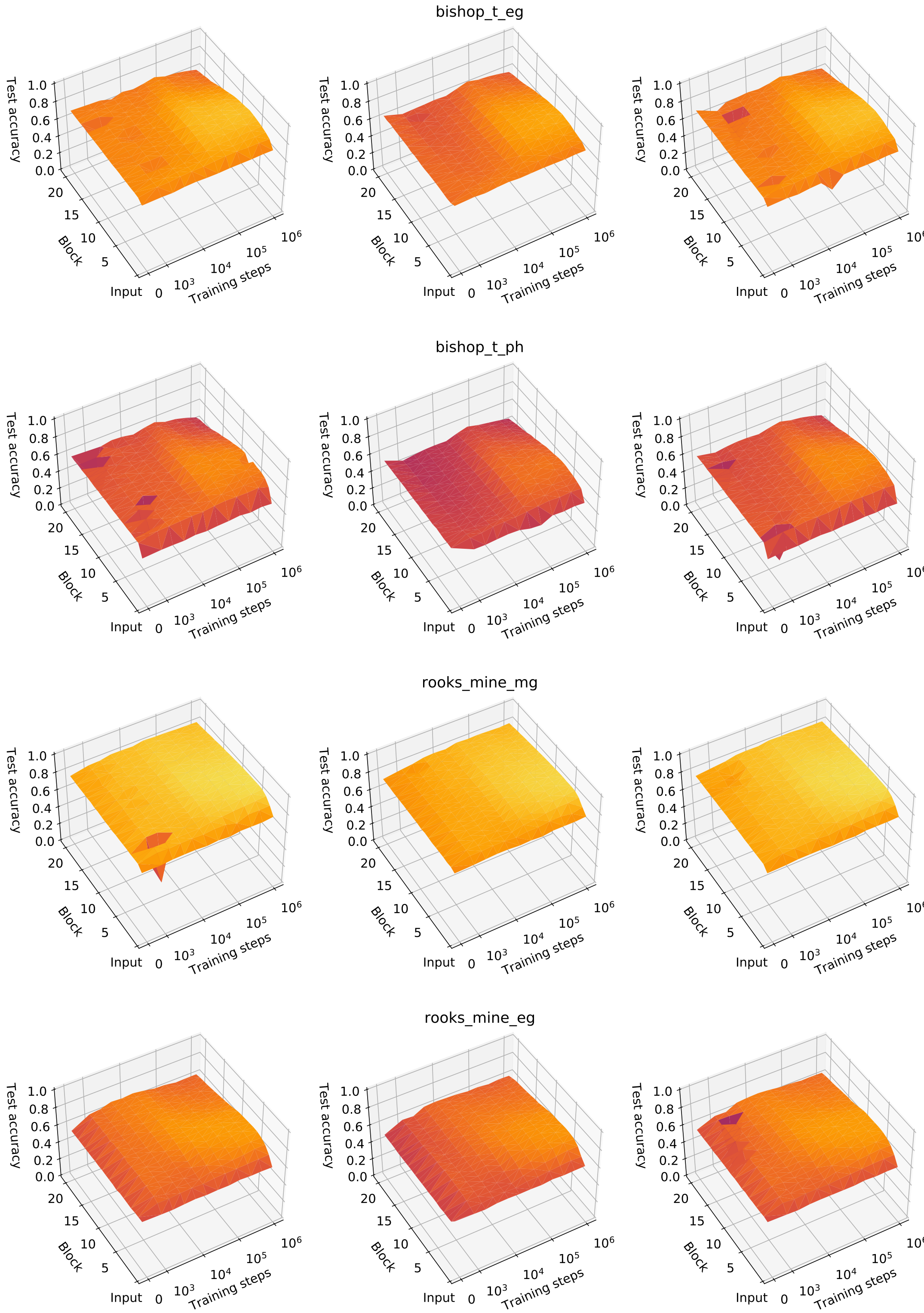

Fig. S41. Comparison of concept regression results between regularisers. Left: unregularised, centre: l_1 regularised, right: group-sparse.

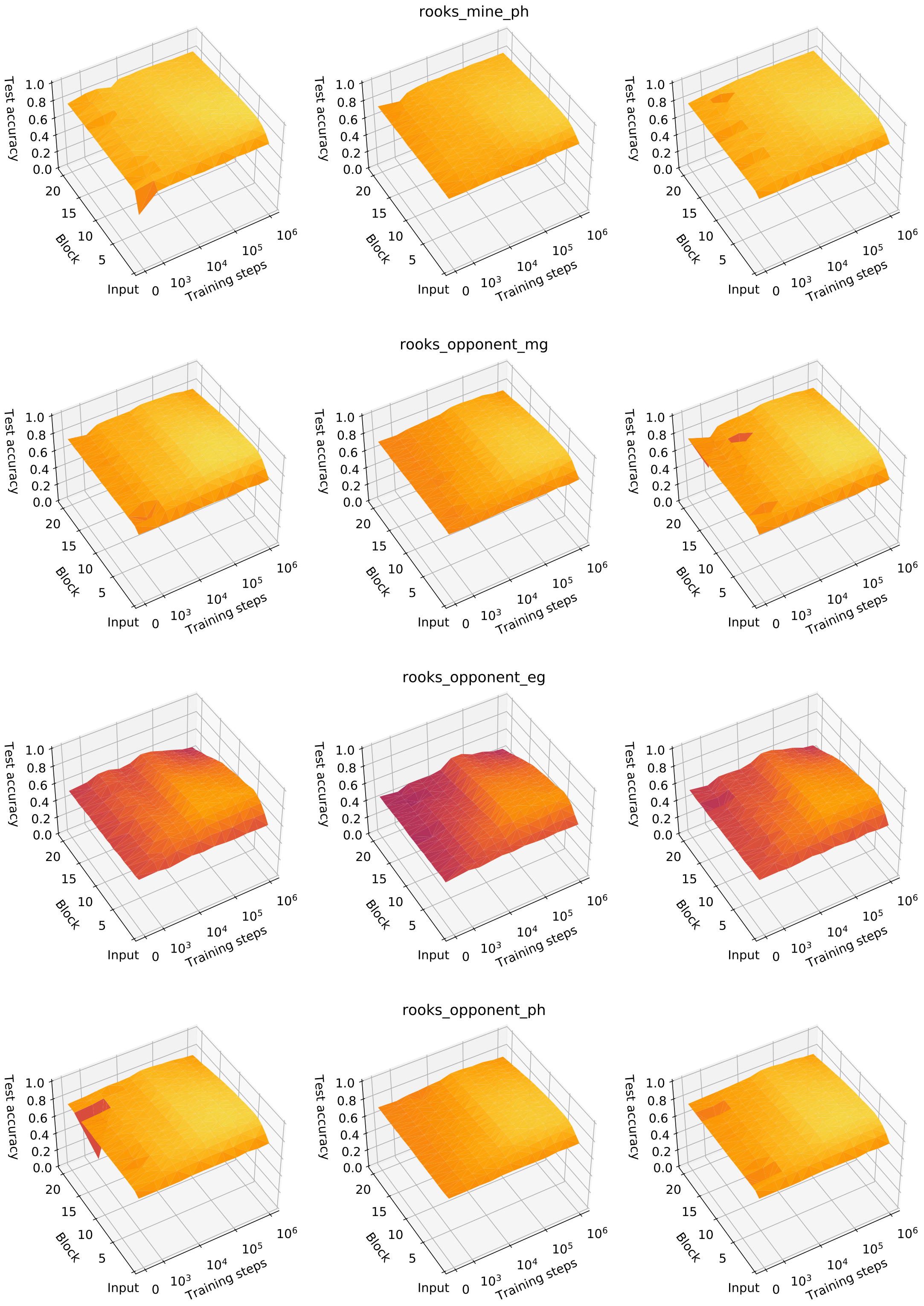

Fig. S42. Comparison of concept regression results between regularisers. Left: unregularised, centre: l_1 regularised, right: group-sparse.

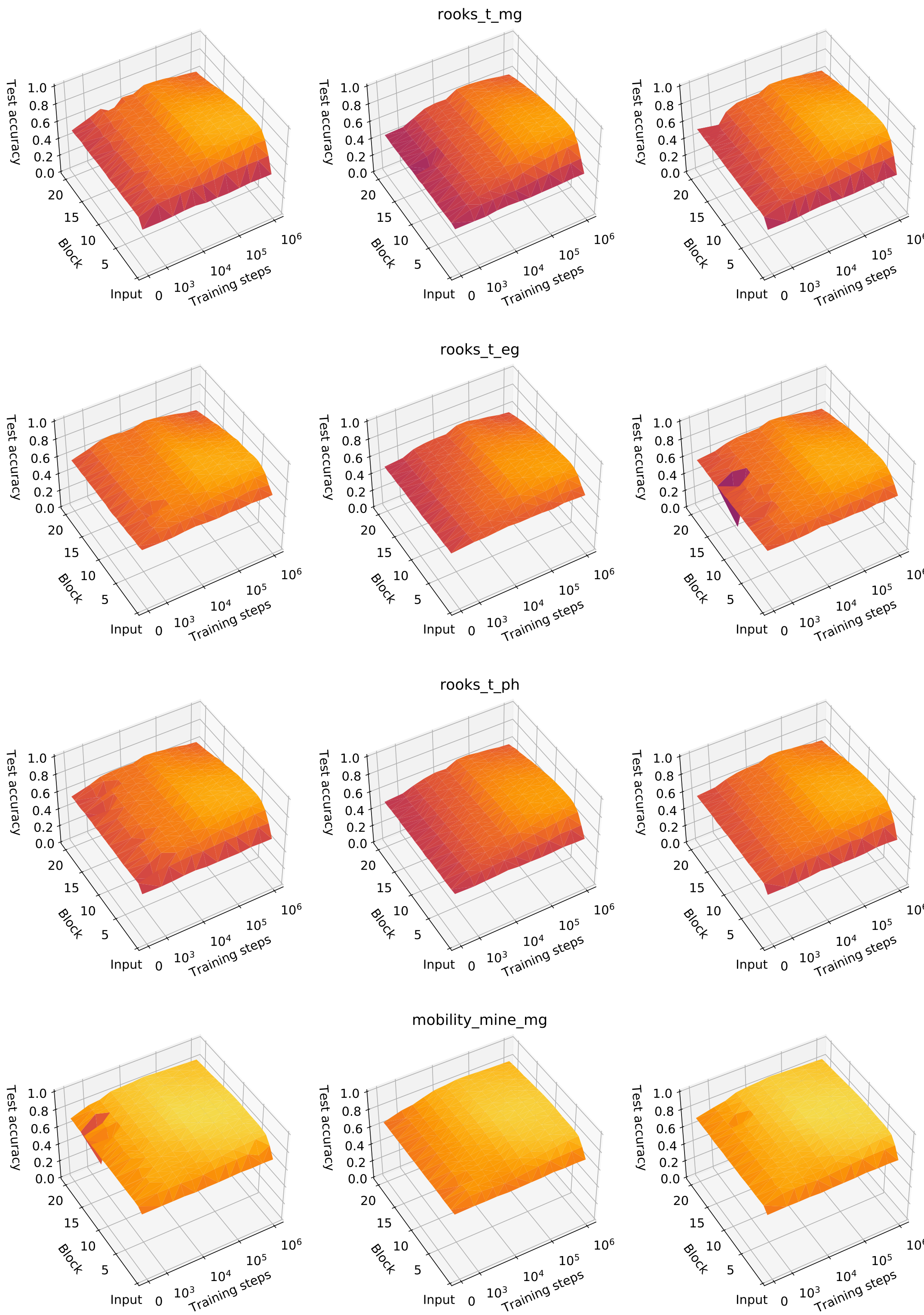

Fig. S43. Comparison of concept regression results between regularisers. Left: unregularised, centre: l_1 regularised, right: group-sparse.

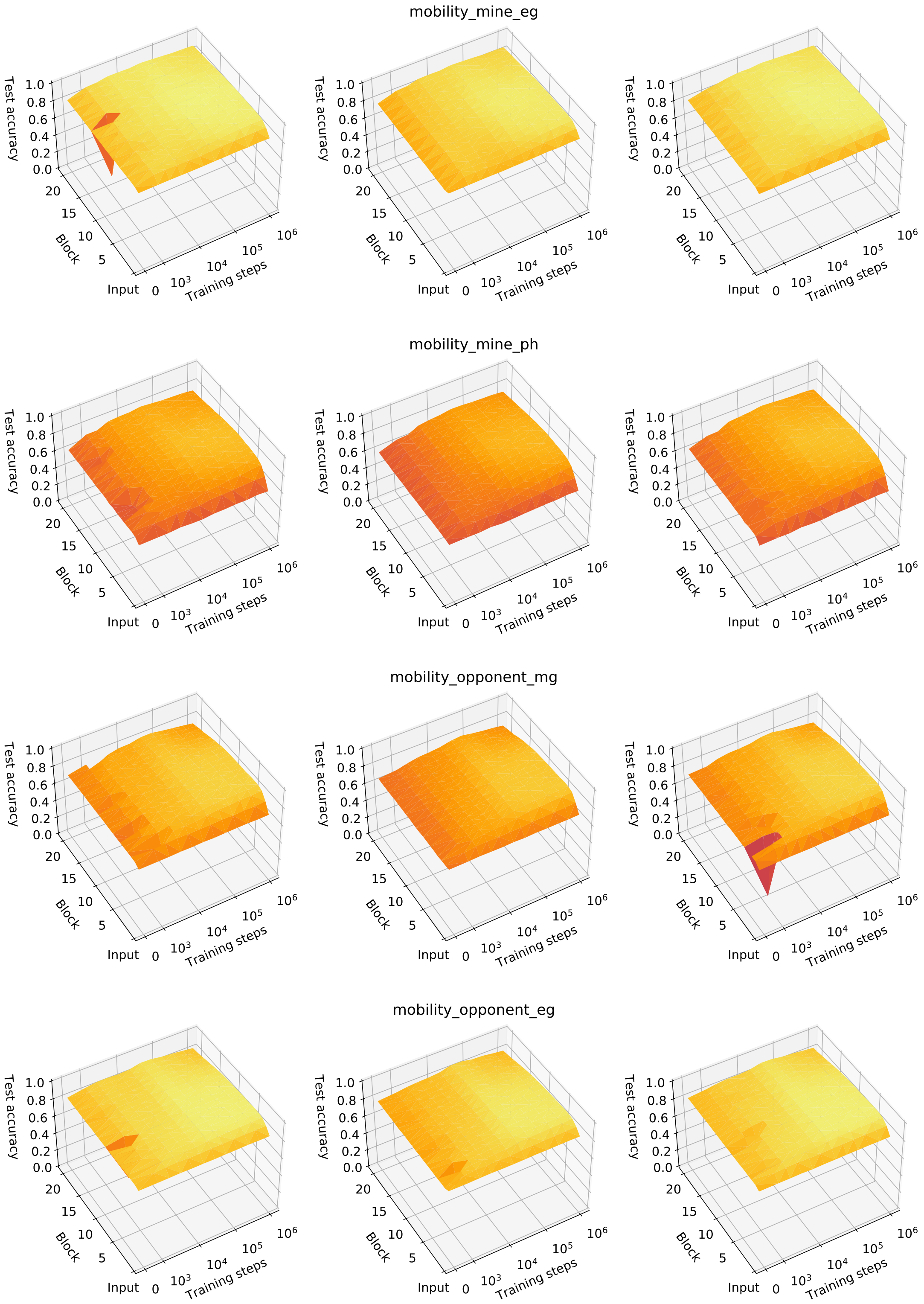

Fig. S44. Comparison of concept regression results between regularisers. Left: unregularised, centre: l_1 regularised, right: group-sparse.

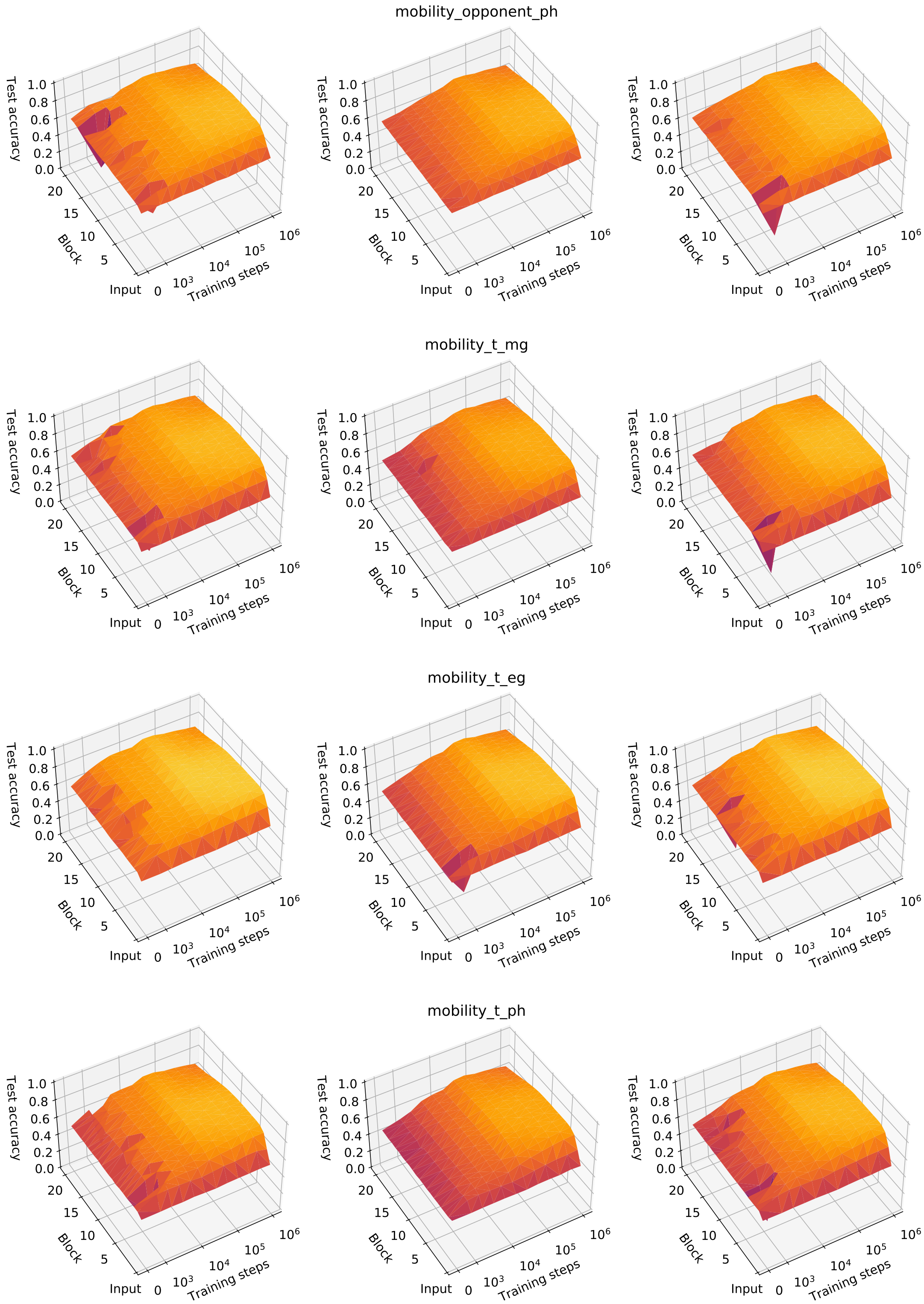

Fig. S45. Comparison of concept regression results between regularisers. Left: unregularised, centre: l_1 regularised, right: group-sparse.

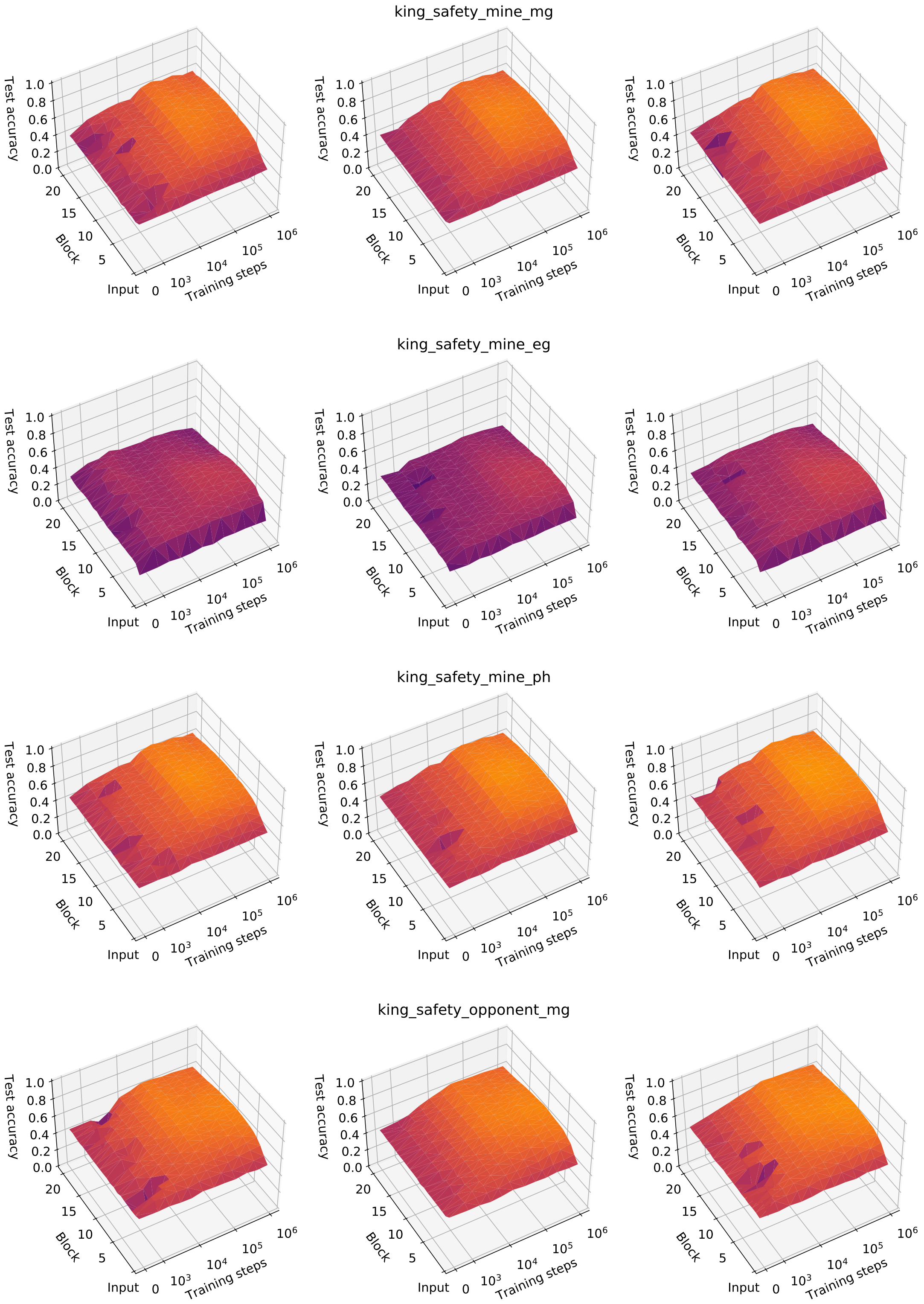

Fig. S46. Comparison of concept regression results between regularisers. Left: unregularised, centre: l_1 regularised, right: group-sparse.

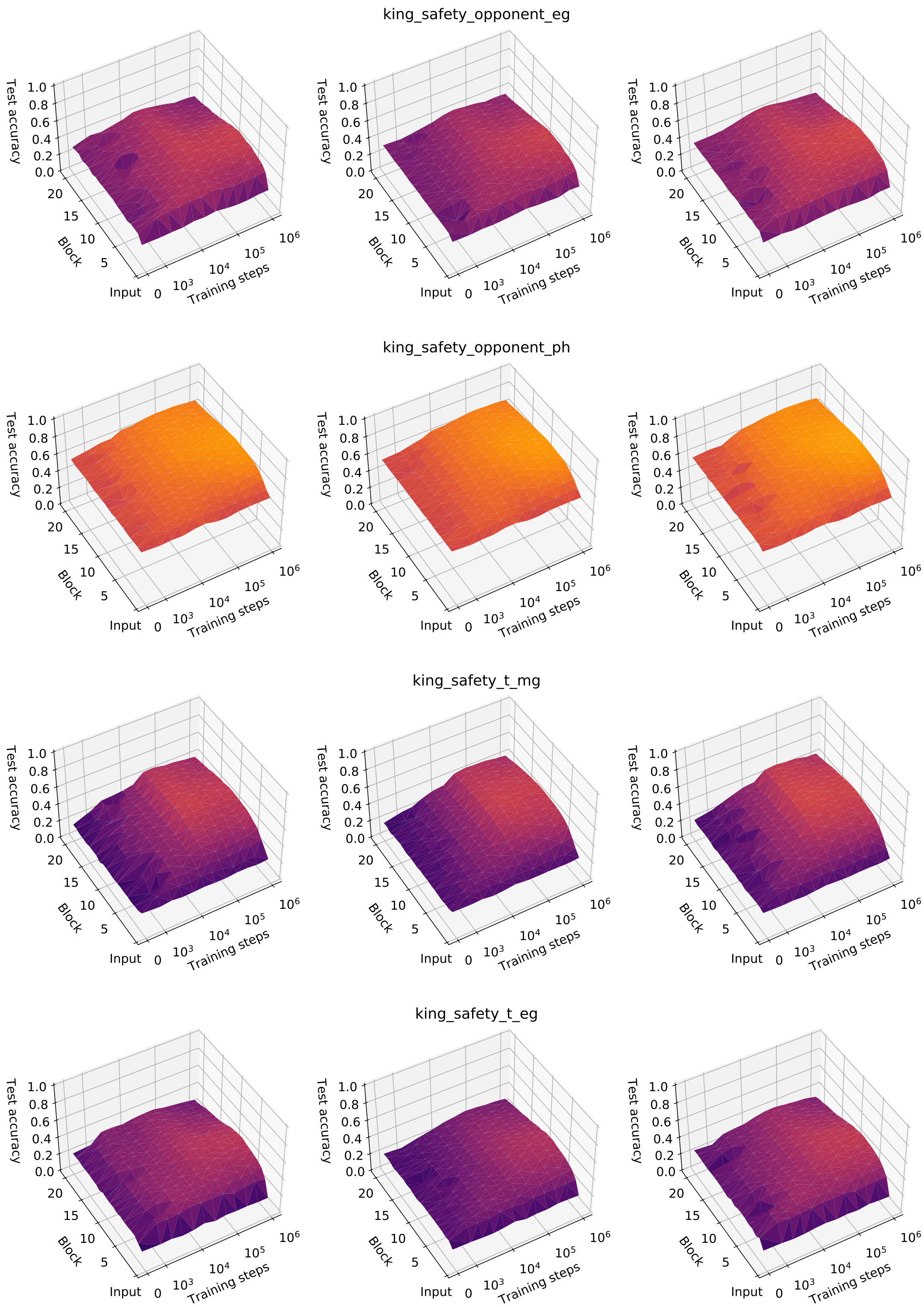

Fig. S47. Comparison of concept regression results between regularisers. Left: unregularised, centre: l_1 regularised, right: group-sparse.

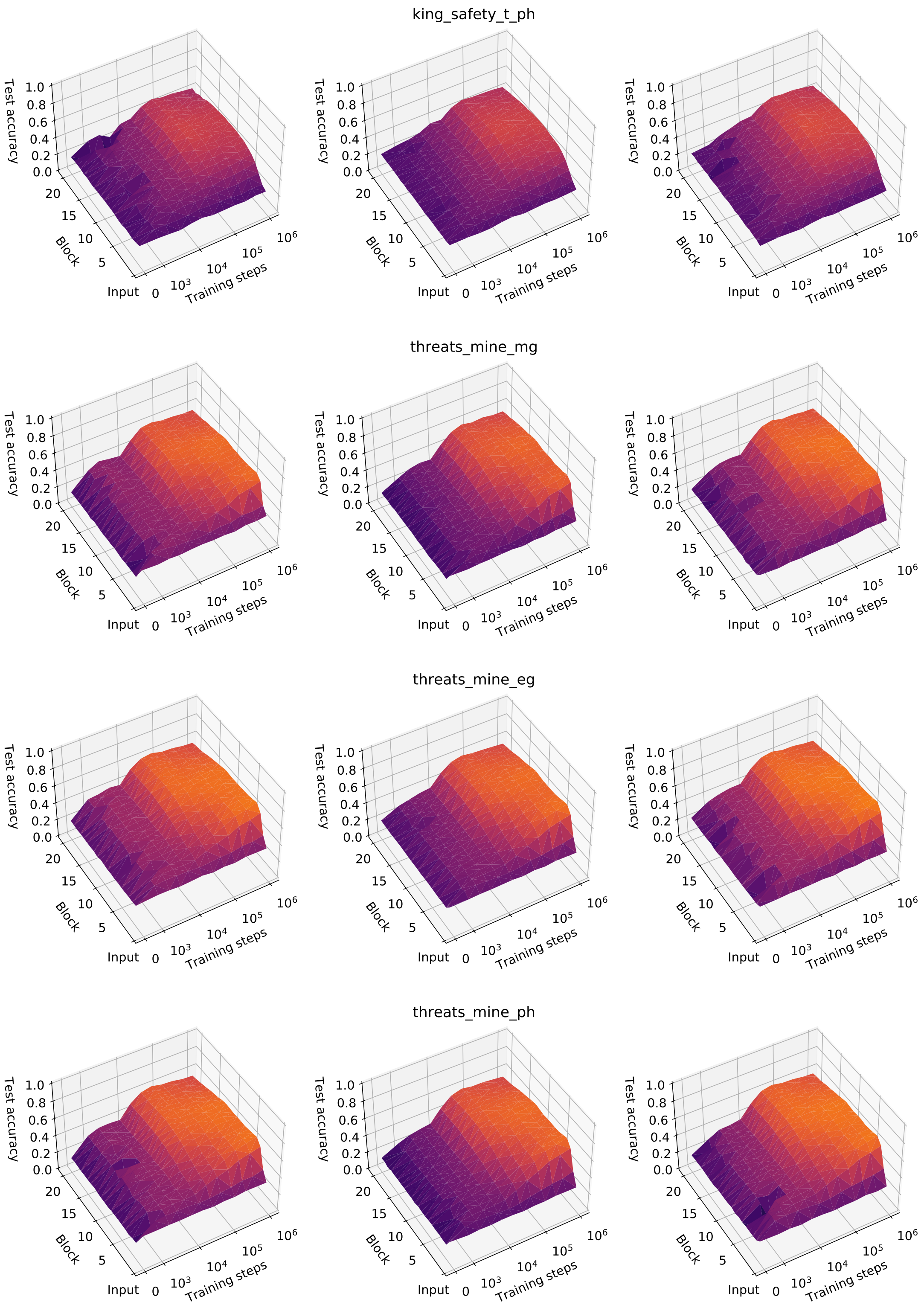

Fig. S48. Comparison of concept regression results between regularisers. Left: unregularised, centre: l_1 regularised, right: group-sparse.

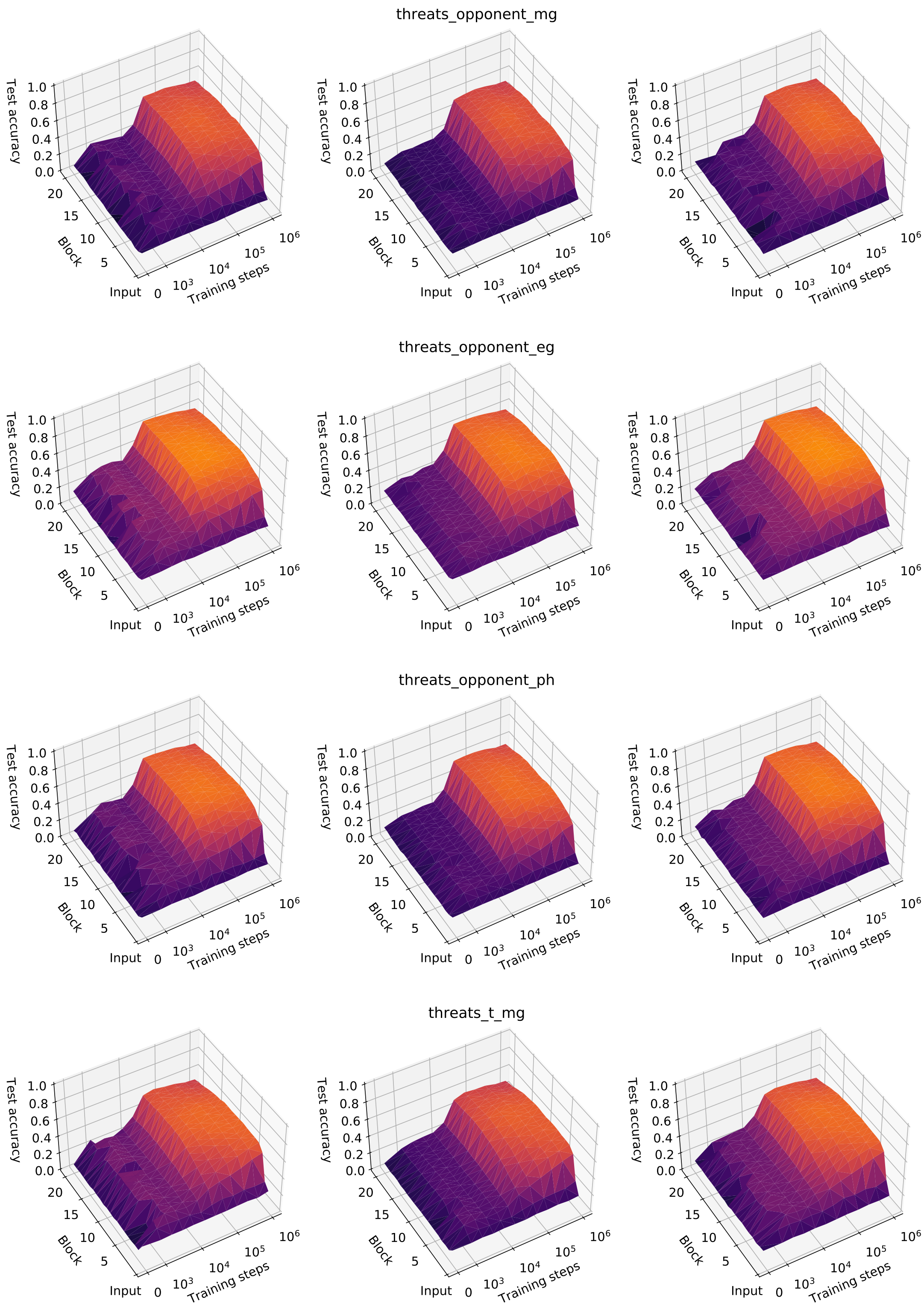

Fig. S49. Comparison of concept regression results between regularisers. Left: unregularised, centre: l_1 regularised, right: group-sparse.

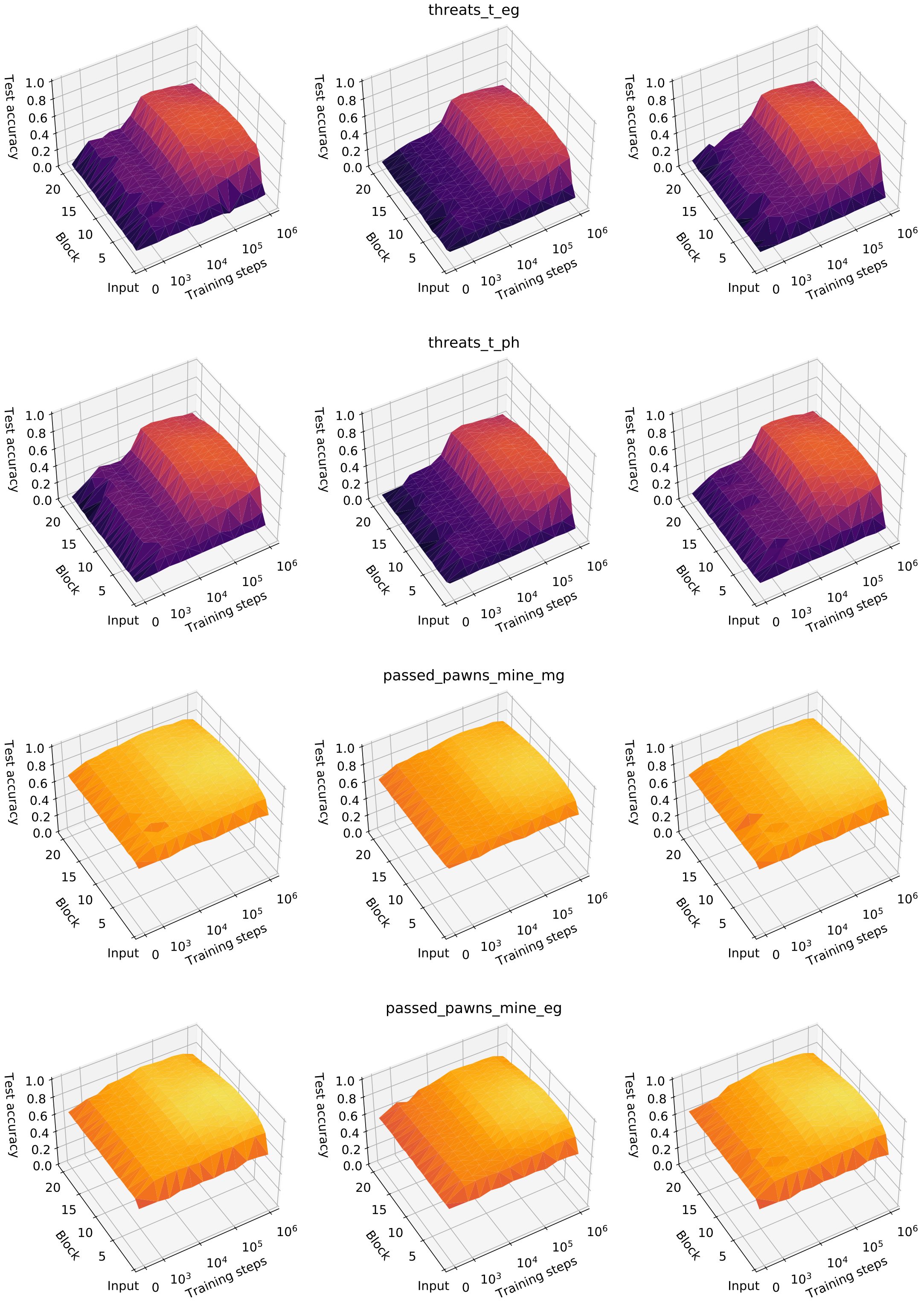

Fig. S50. Comparison of concept regression results between regularisers. Left: unregularised, centre: l_1 regularised, right: group-sparse.

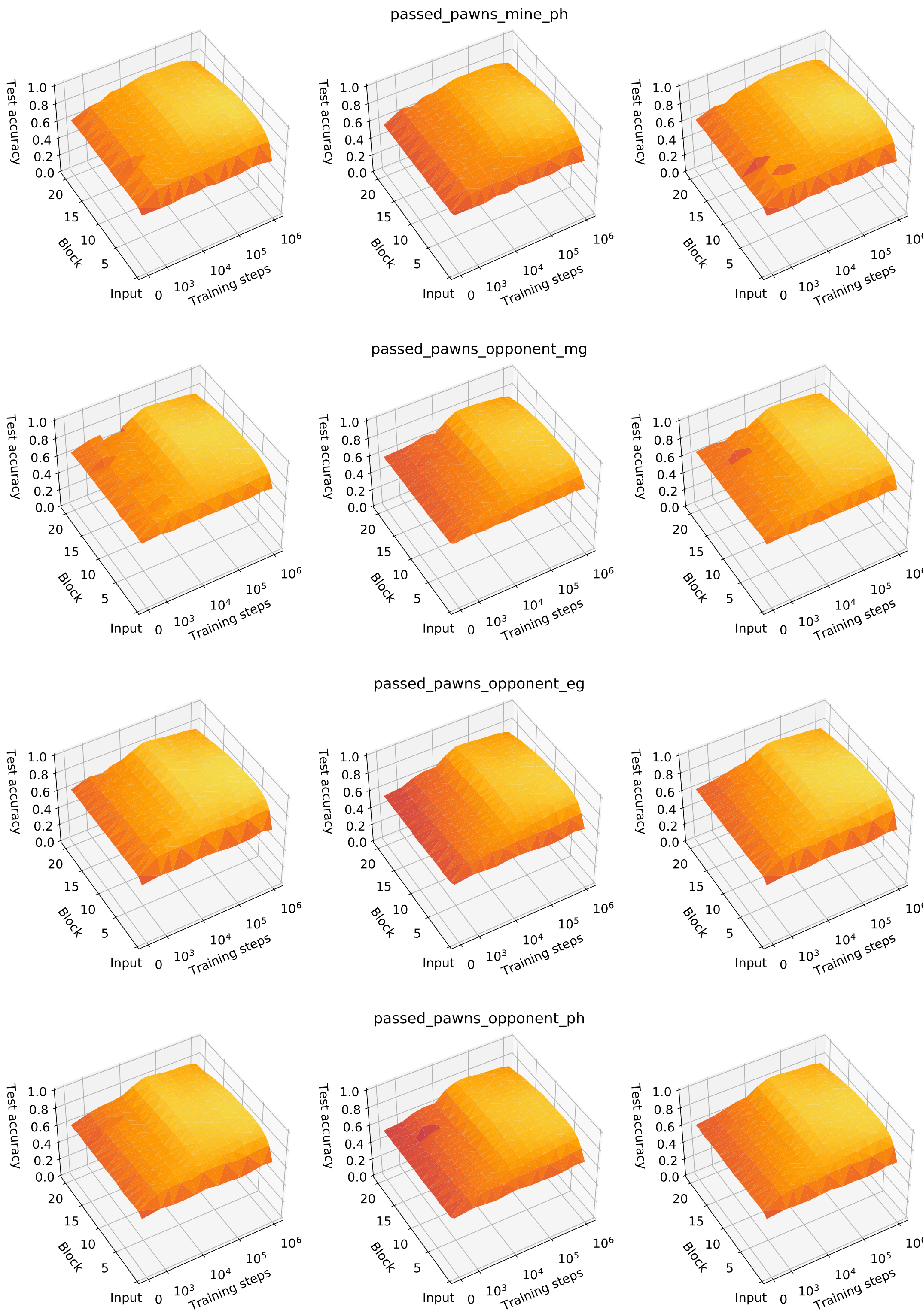

Fig. S51. Comparison of concept regression results between regularisers. Left: unregularised, centre: l_1 regularised, right: group-sparse.

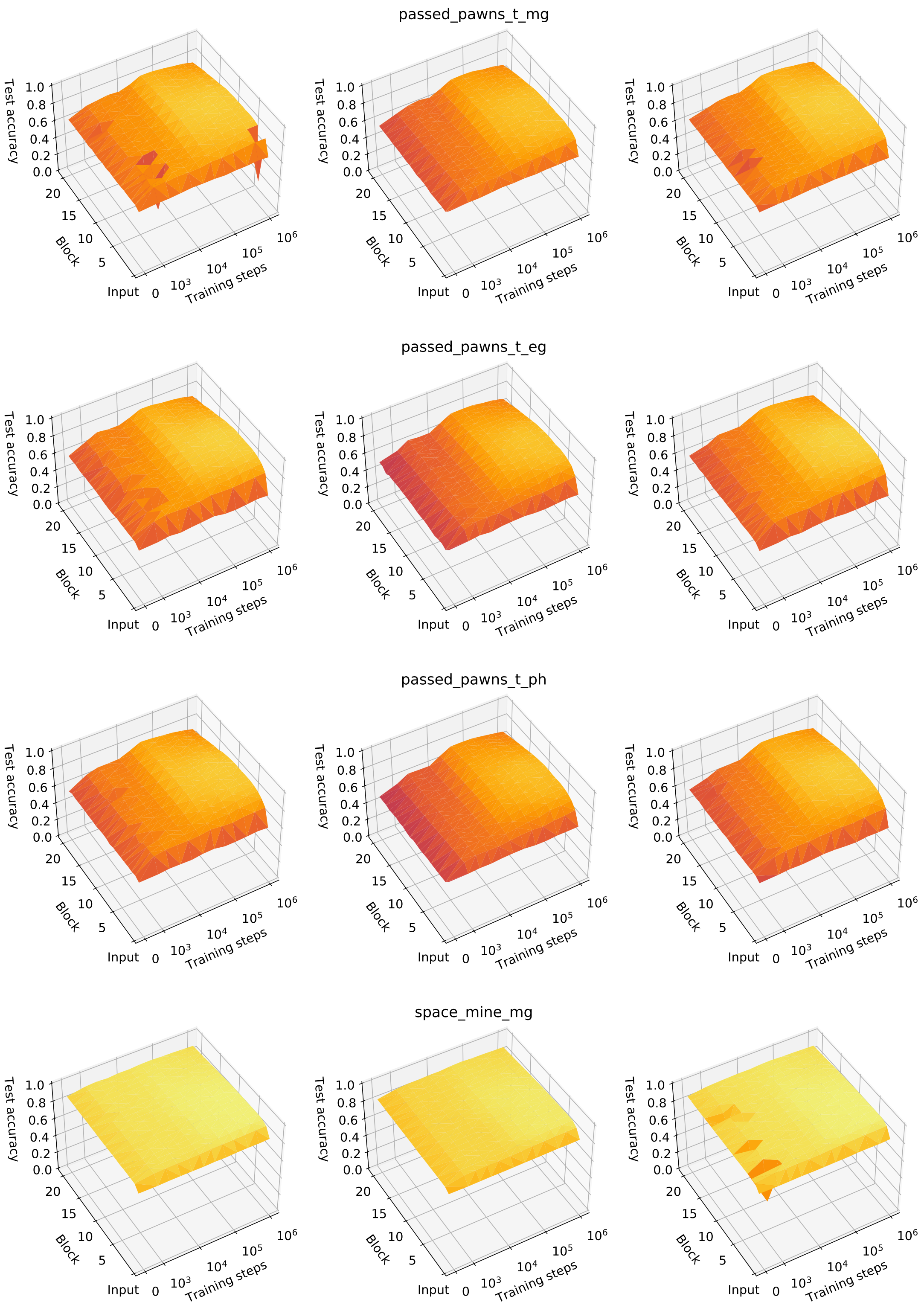

Fig. S52. Comparison of concept regression results between regularisers. Left: unregularised, centre: l_1 regularised, right: group-sparse.

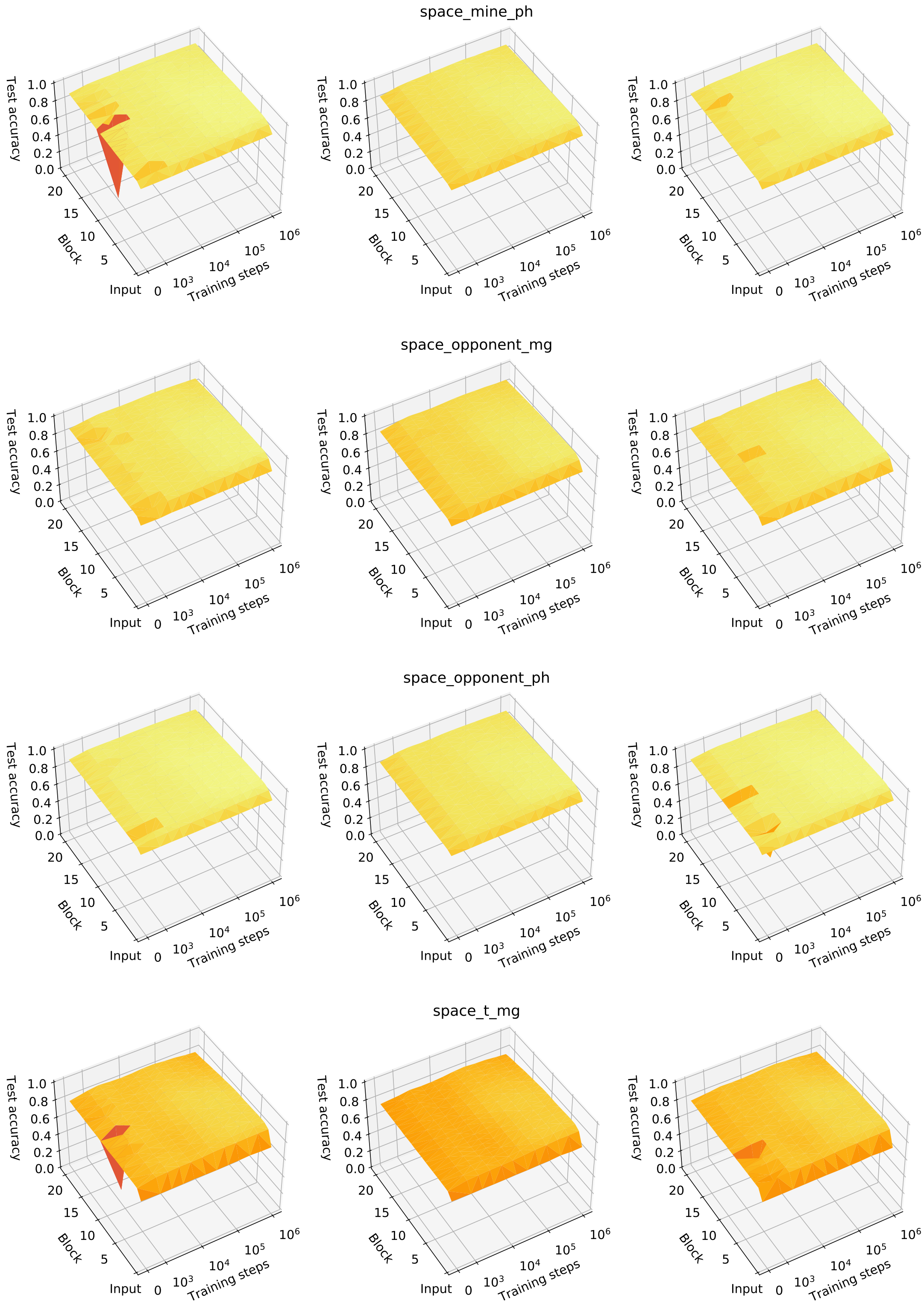

Fig. S53. Comparison of concept regression results between regularisers. Left: unregularised, centre: l_1 regularised, right: group-sparse.

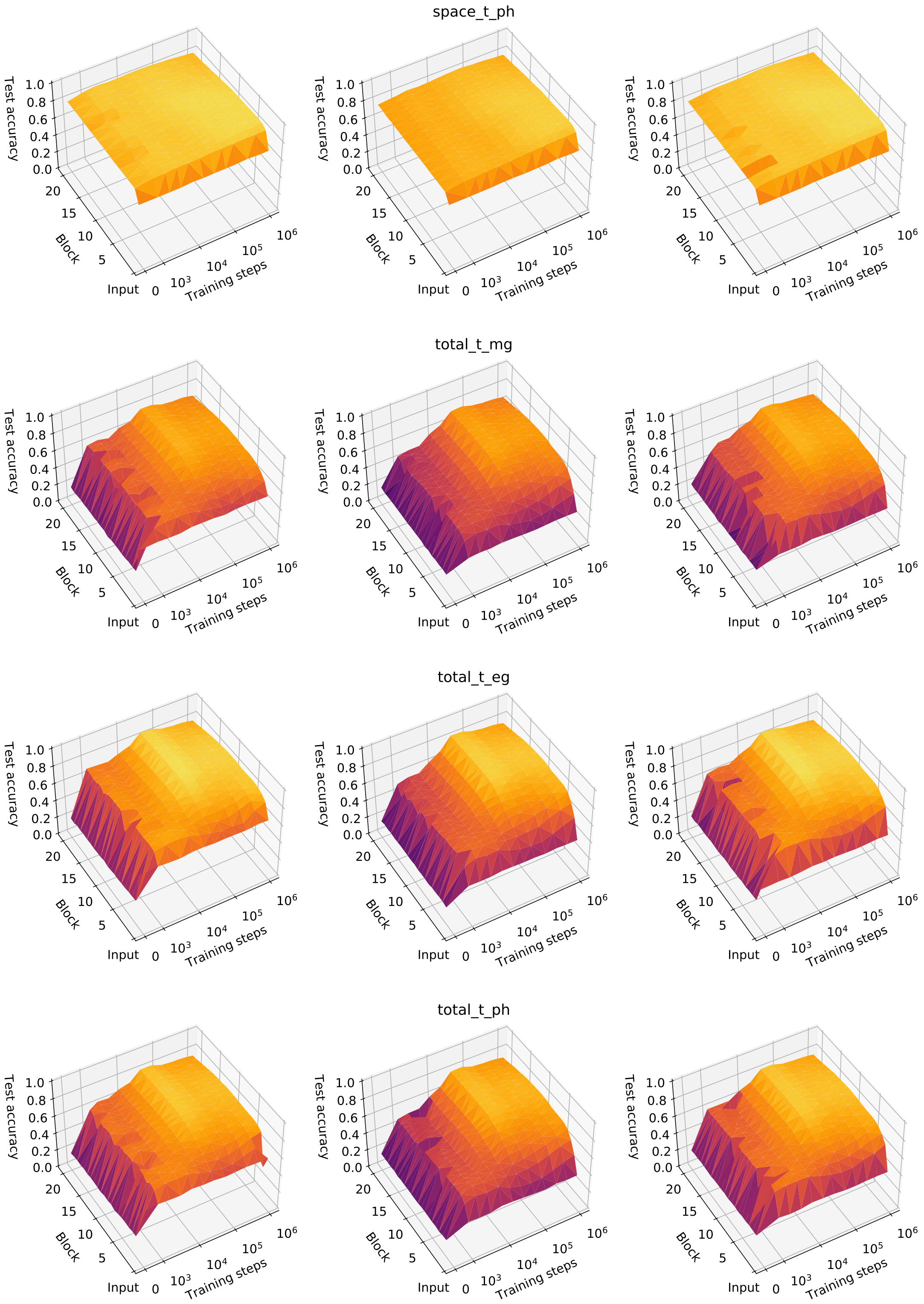

Fig. S54. Comparison of concept regression results between regularisers. Left: unregularised, centre: l_1 regularised, right: group-sparse.

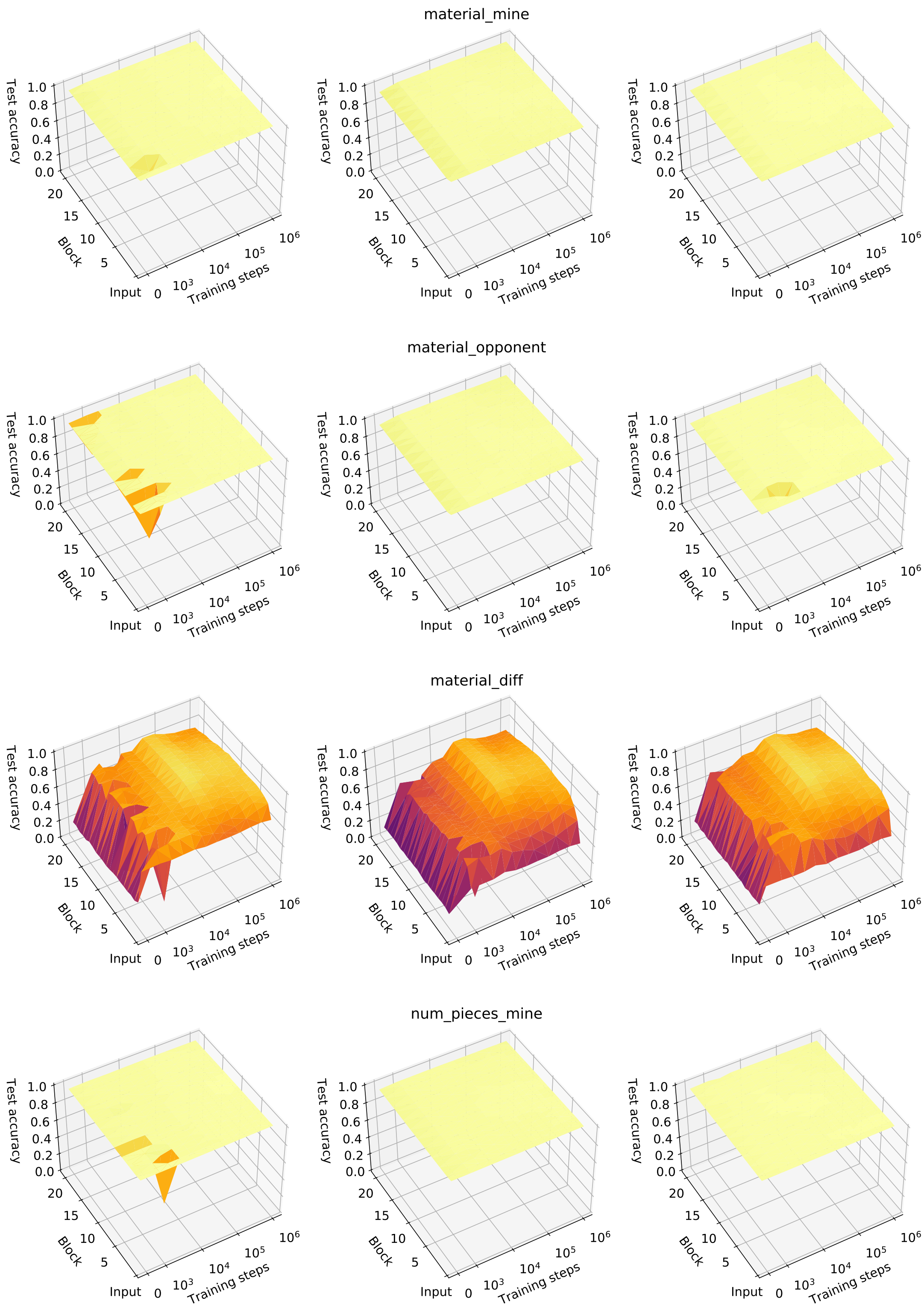

Fig. S55. Comparison of concept regression results between regularisers. Left: unregularised, centre: l_1 regularised, right: group-sparse.

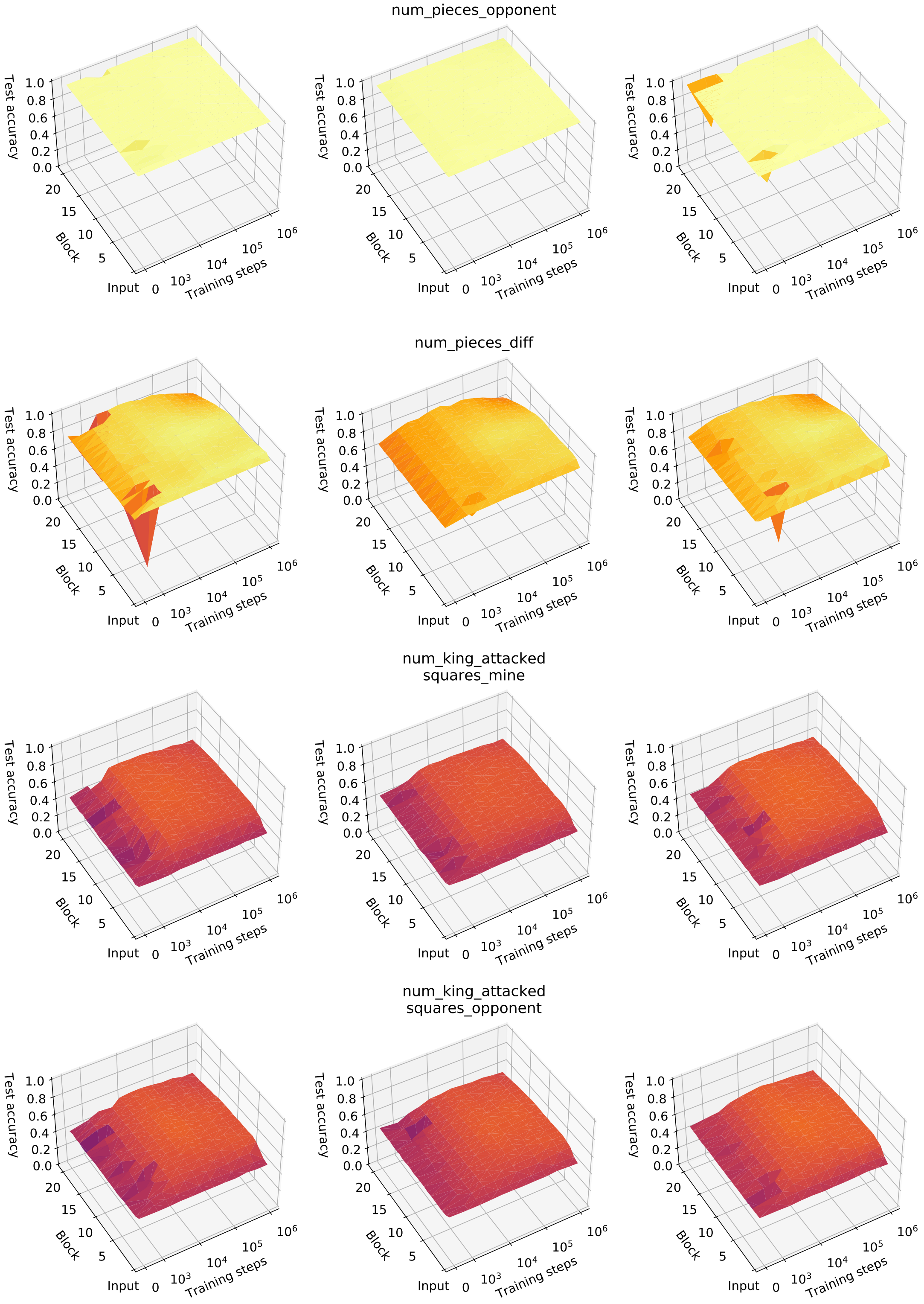

Fig. S56. Comparison of concept regression results between regularisers. Left: unregularised, centre: l_1 regularised, right: group-sparse.

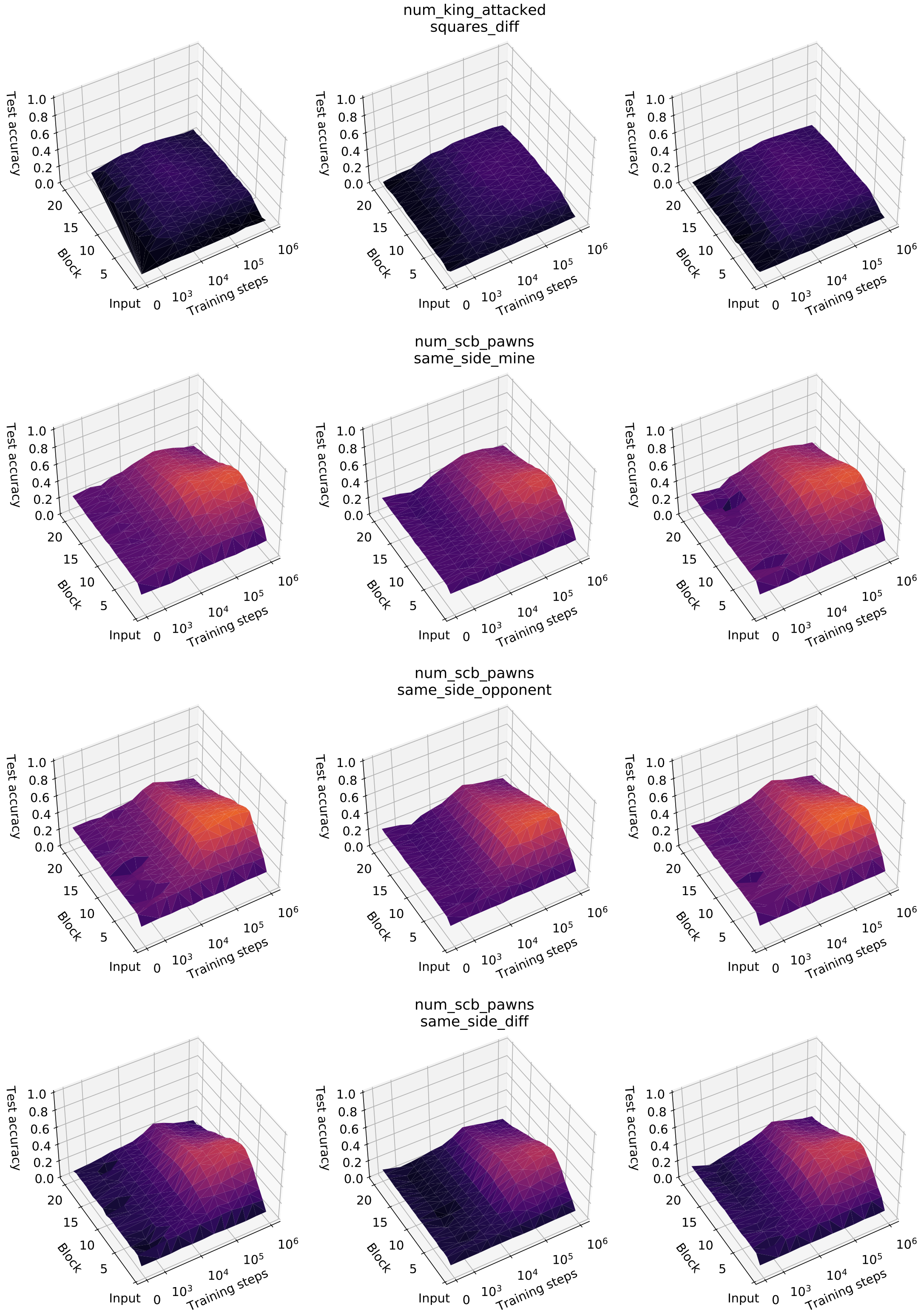

Fig. S57. Comparison of concept regression results between regularisers. Left: unregularised, centre: l_1 regularised, right: group-sparse.

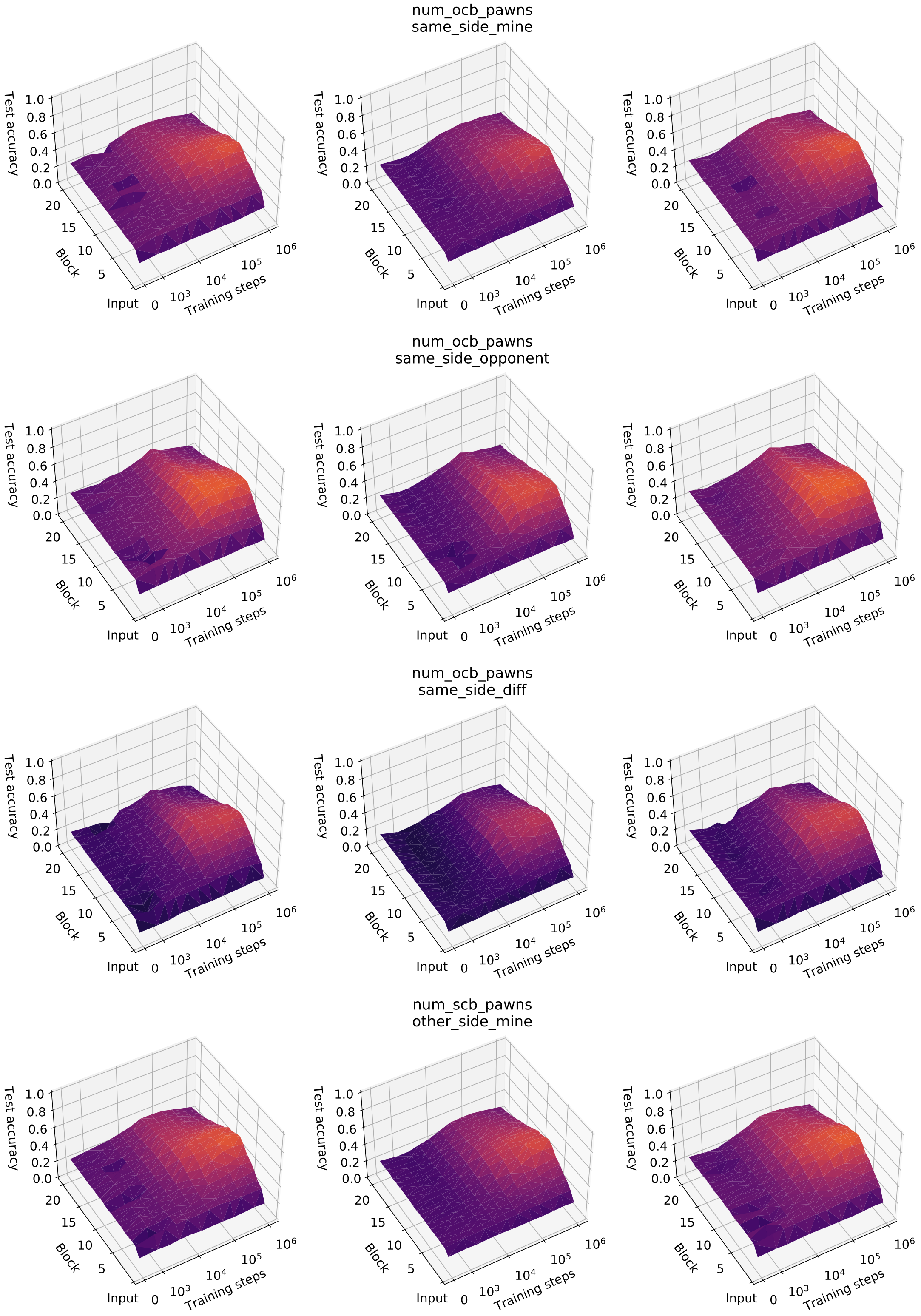

Fig. S58. Comparison of concept regression results between regularisers. Left: unregularised, centre: l_1 regularised, right: group-sparse.

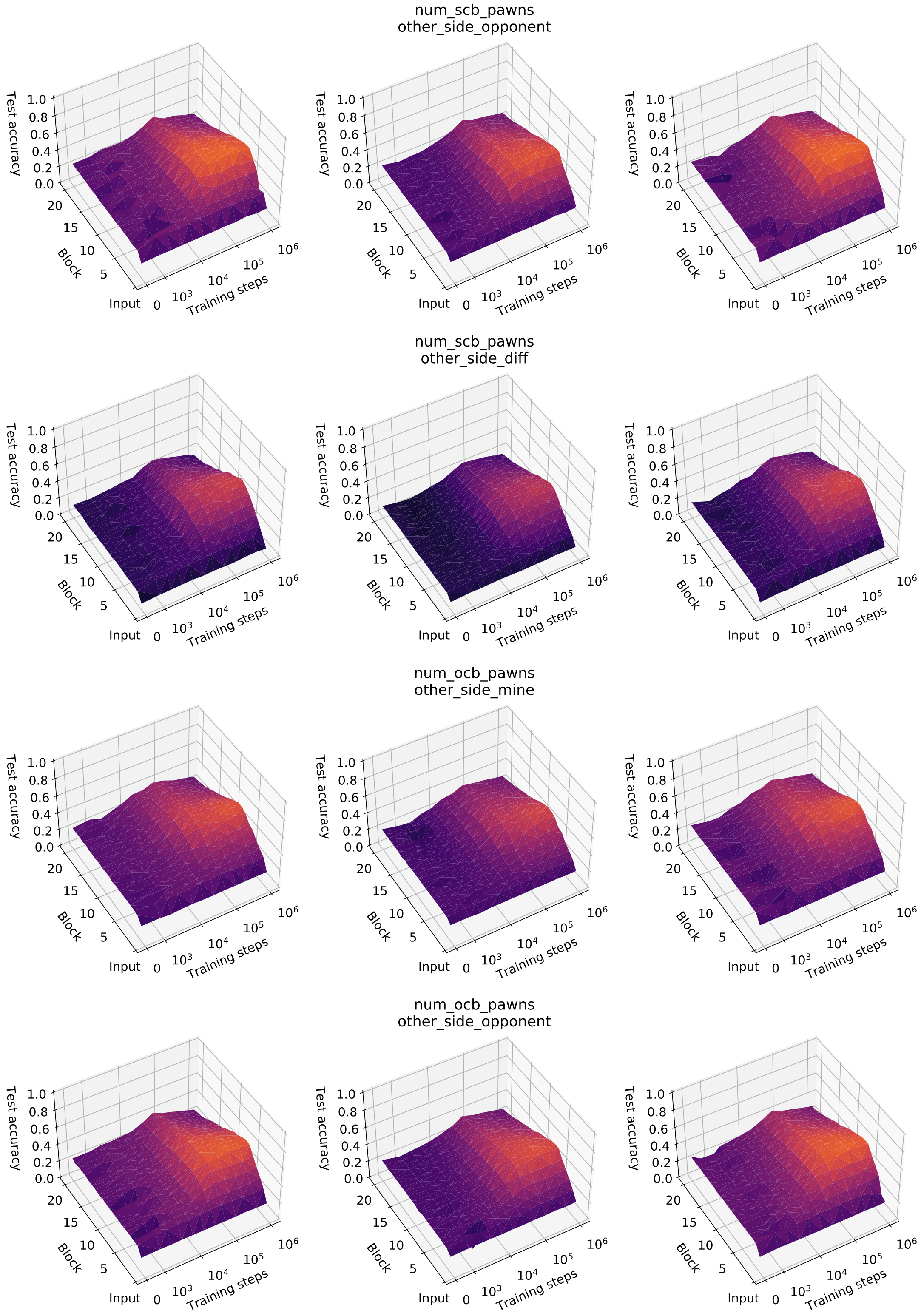

Fig. S59. Comparison of concept regression results between regularisers. Left: unregularised, centre: l_1 regularised, right: group-sparse.

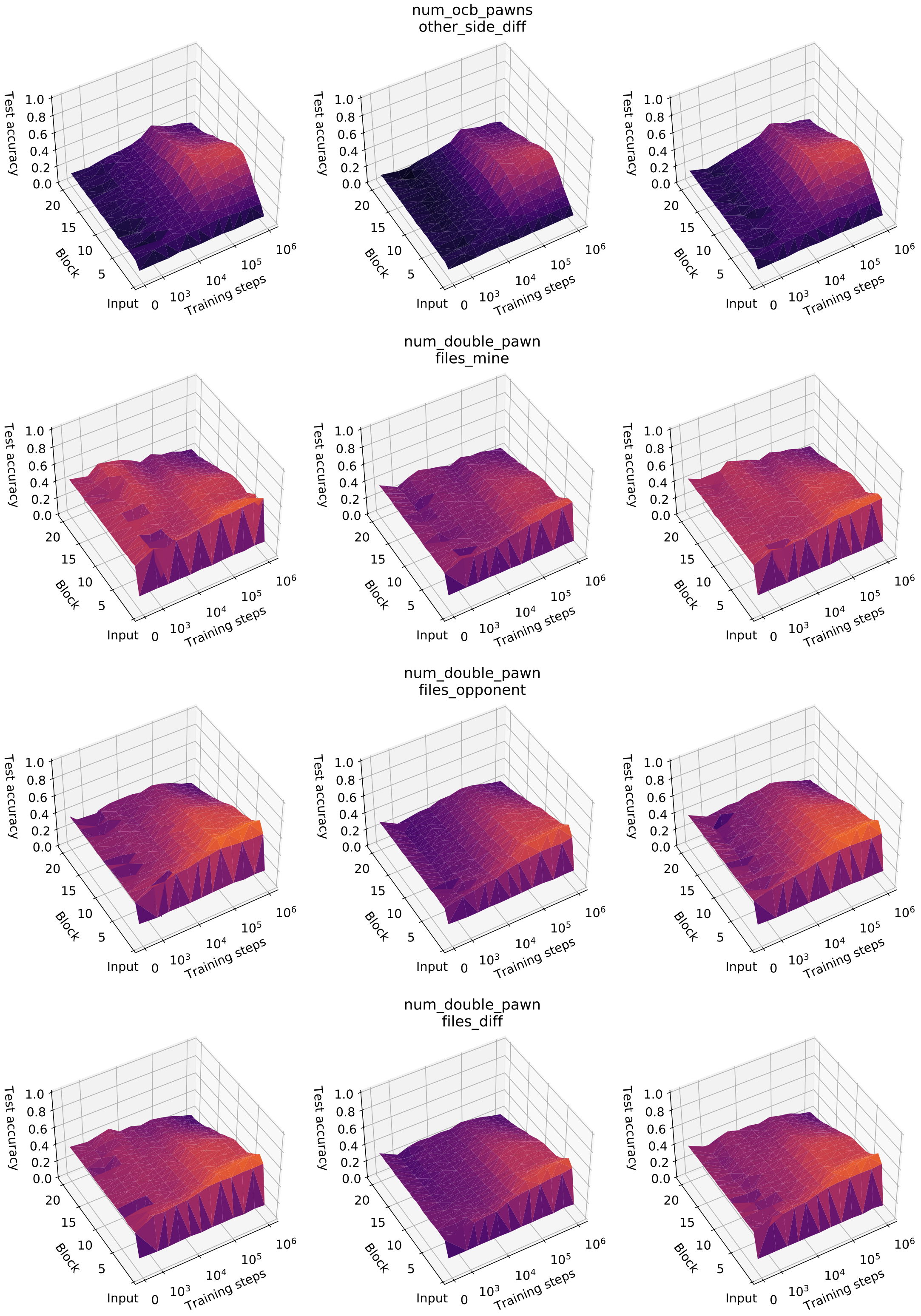

Fig. S60. Comparison of concept regression results between regularisers. Left: unregularised, centre: l_1 regularised, right: group-sparse.

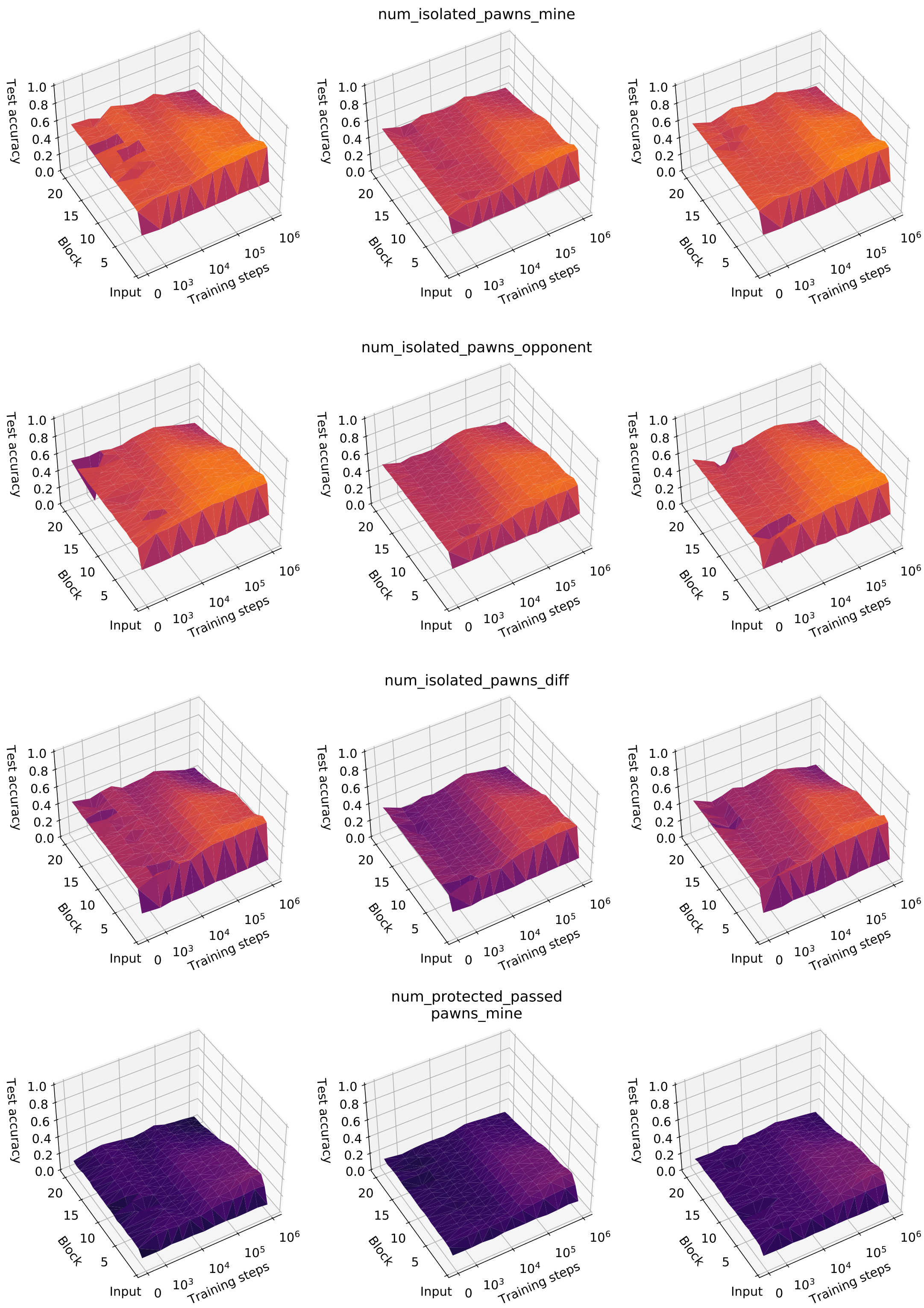

Fig. S61. Comparison of concept regression results between regularisers. Left: unregularised, centre: l_1 regularised, right: group-sparse.

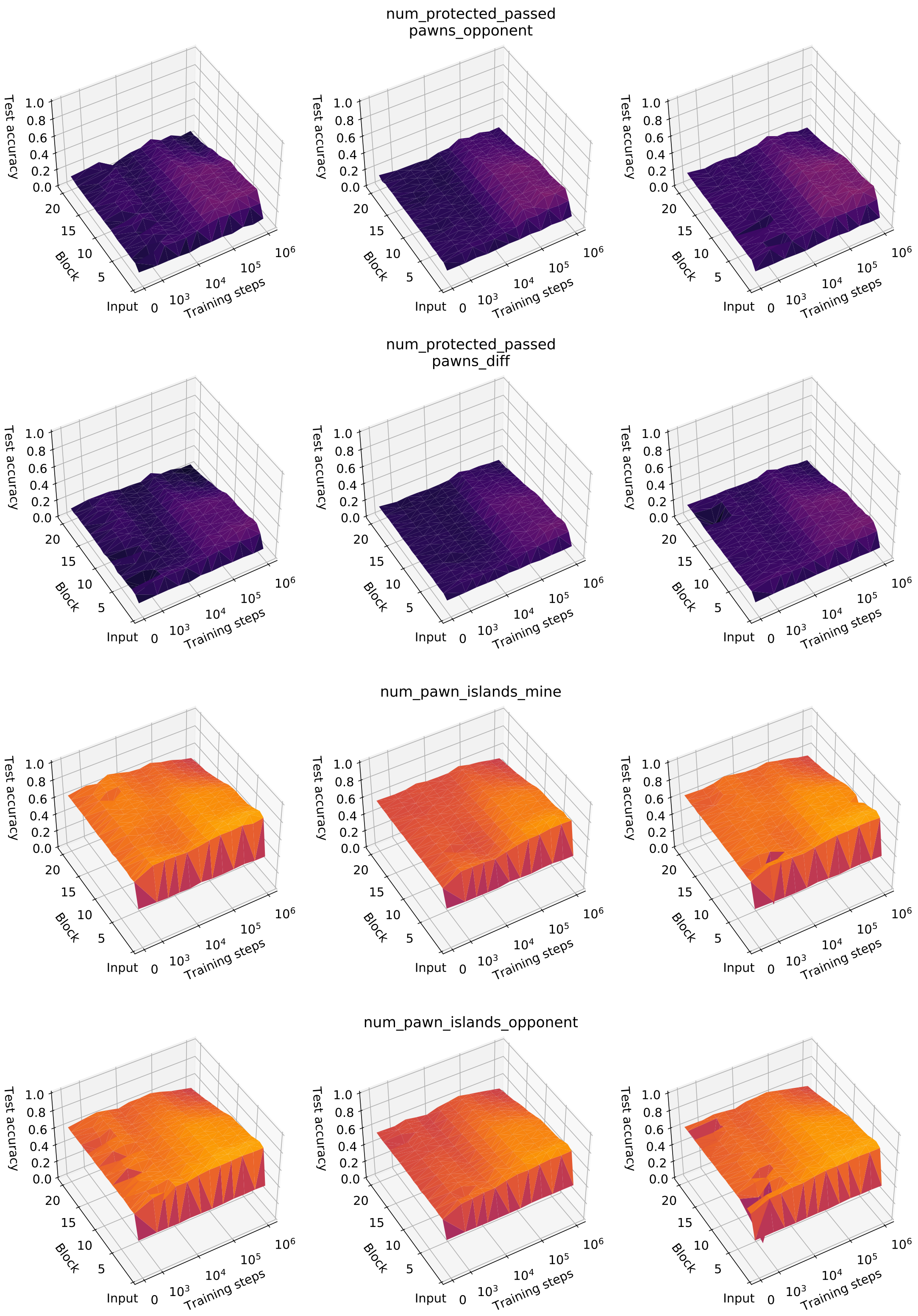

Fig. S62. Comparison of concept regression results between regularisers. Left: unregularised, centre: l_1 regularised, right: group-sparse.

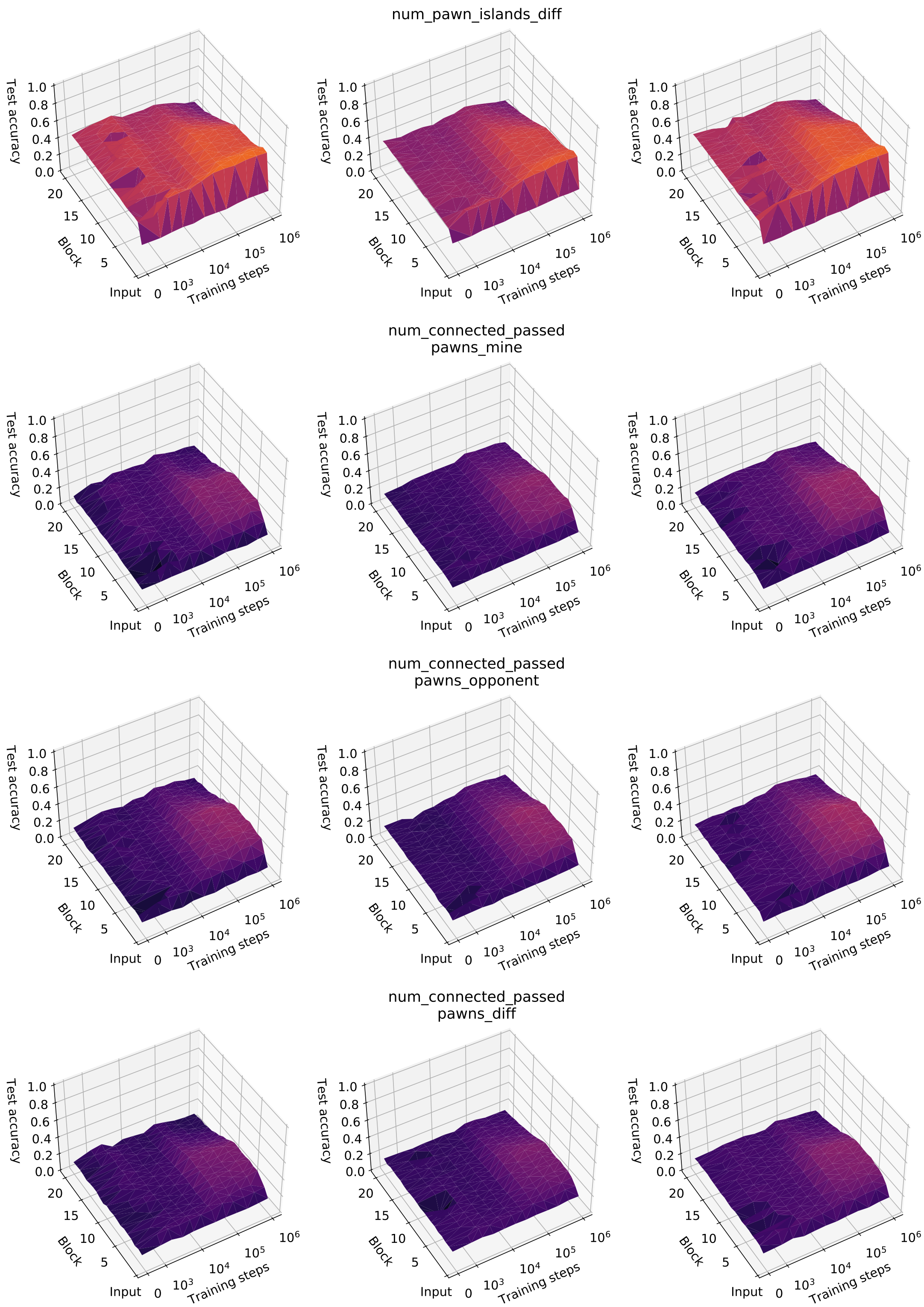

Fig. S63. Comparison of concept regression results between regularisers. Left: unregularised, centre: l_1 regularised, right: group-sparse.

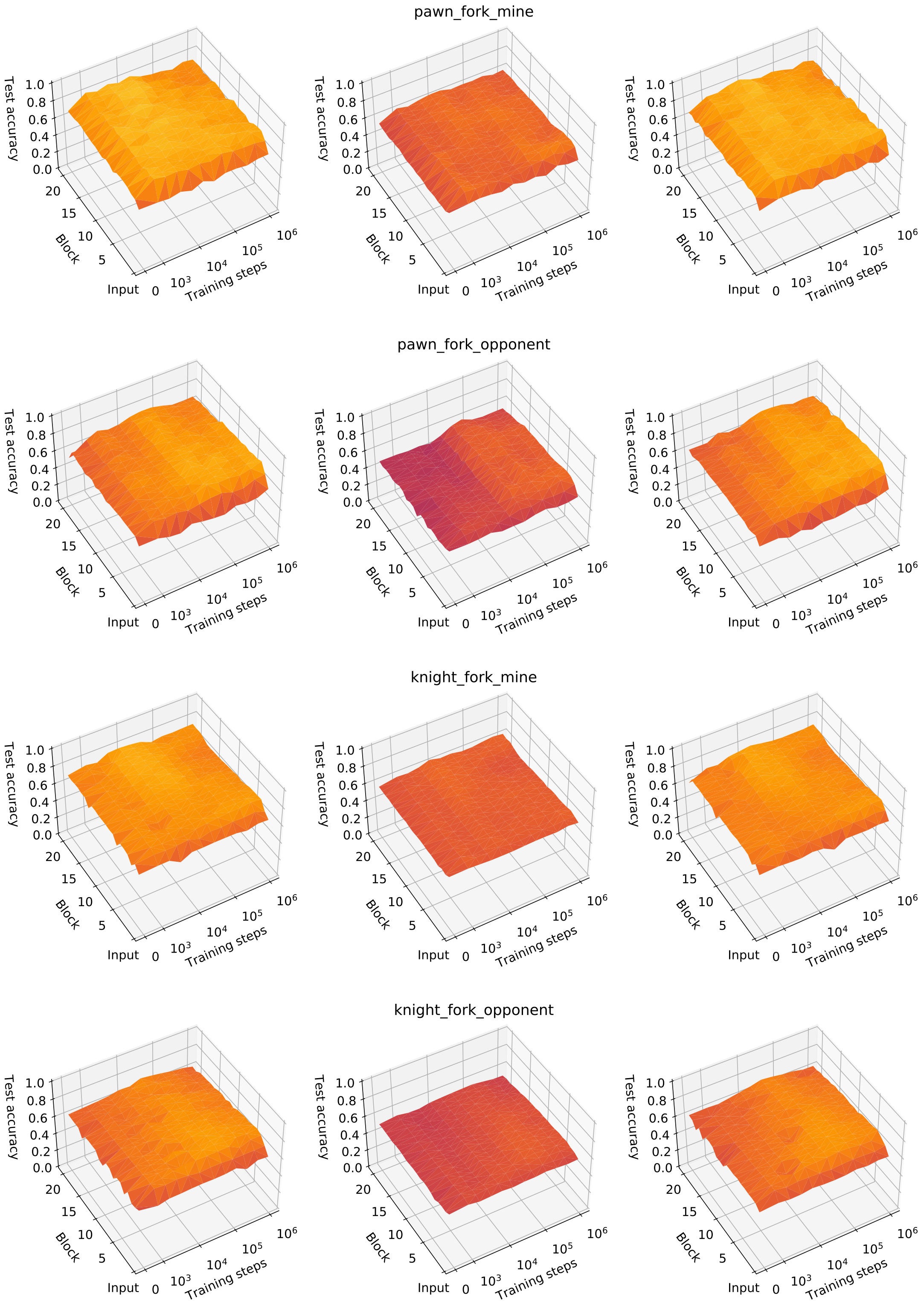

Fig. S64. Comparison of concept regression results between regularisers. Left: unregularised, centre: l_1 regularised, right: group-sparse.

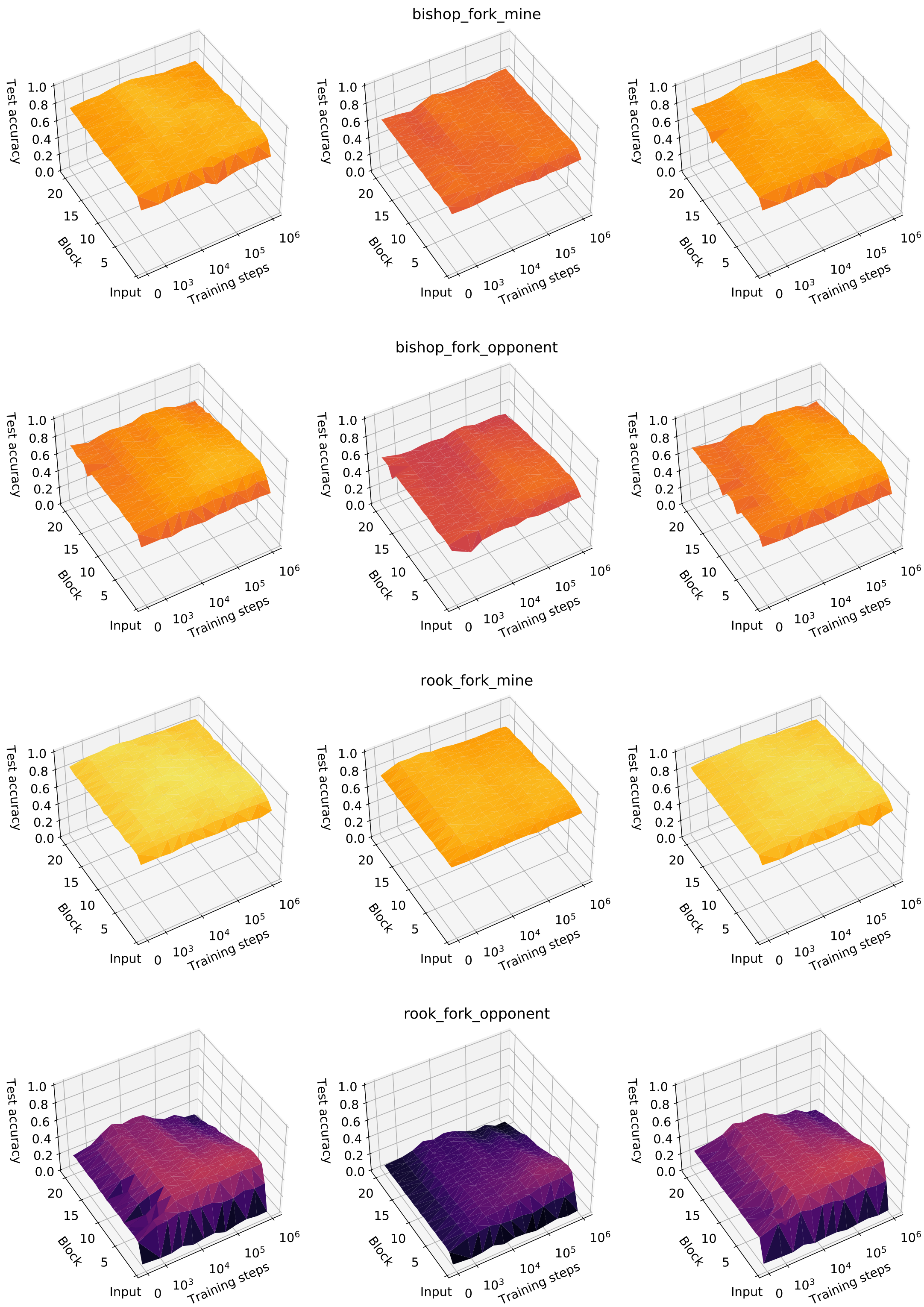

Fig. S65. Comparison of concept regression results between regularisers. Left: unregularised, centre: l_1 regularised, right: group-sparse.

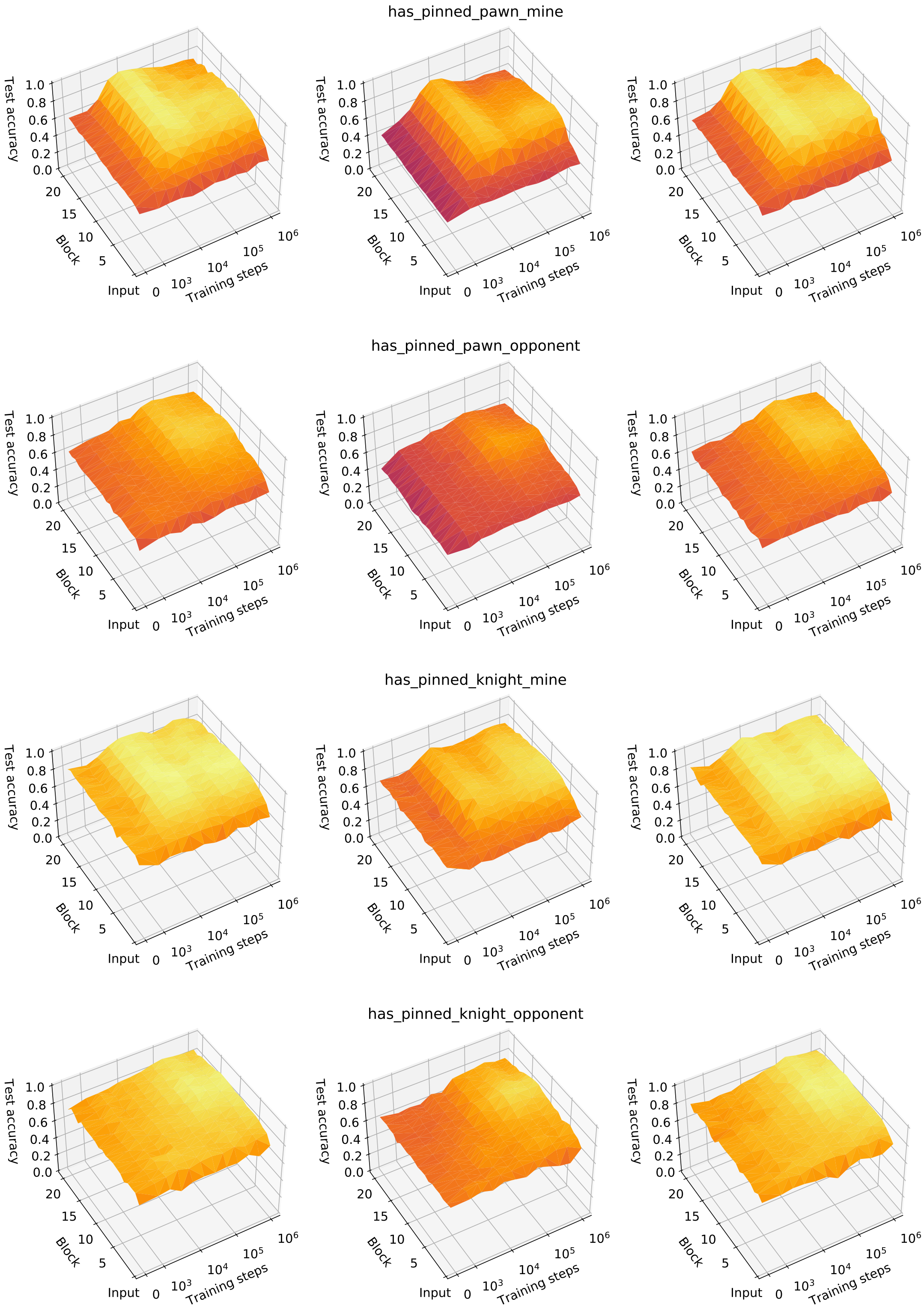

Fig. S66. Comparison of concept regression results between regularisers. Left: unregularised, centre: l_1 regularised, right: group-sparse.

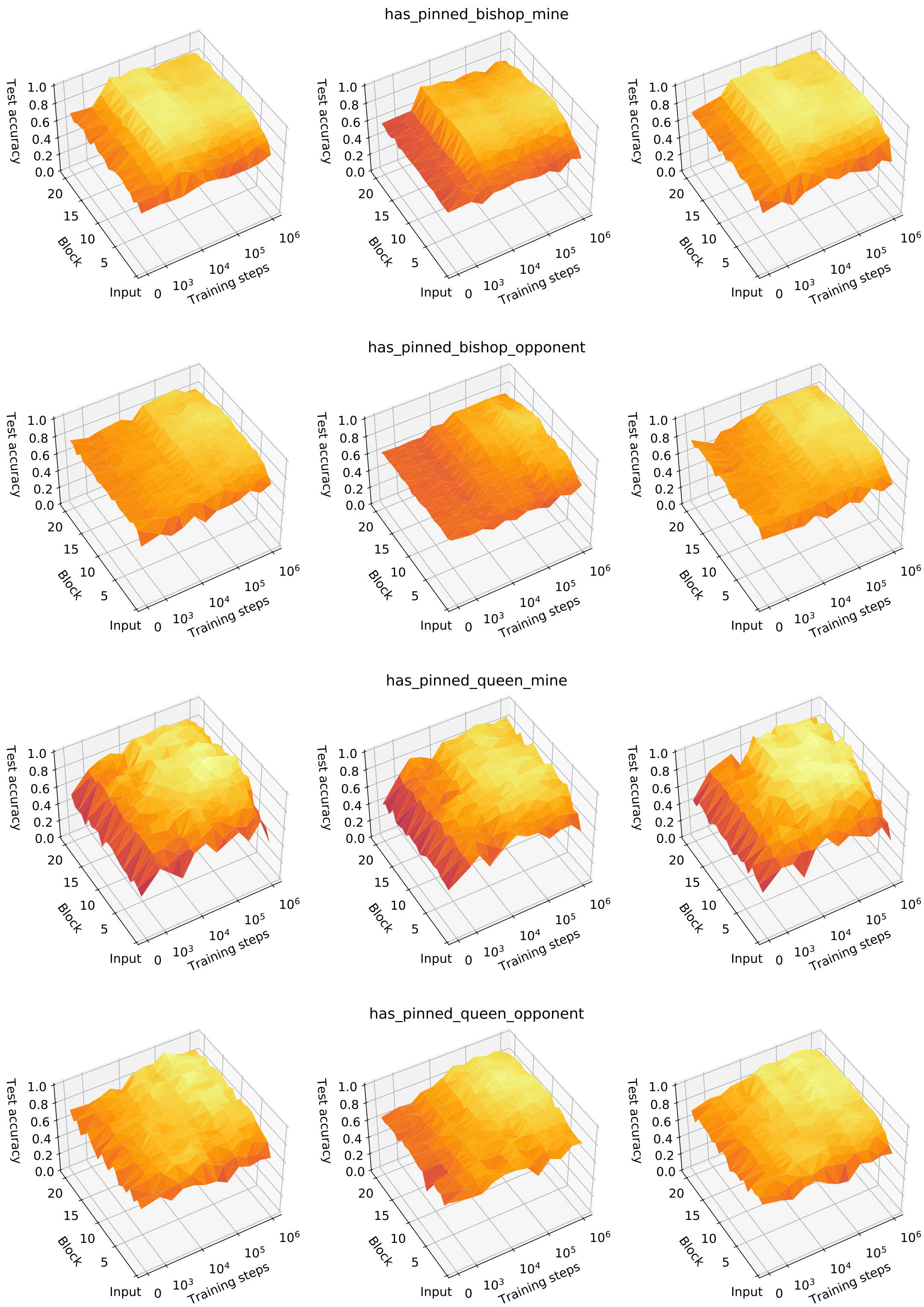

Fig. S67. Comparison of concept regression results between regularisers. Left: unregularised, centre: l_1 regularised, right: group-sparse.

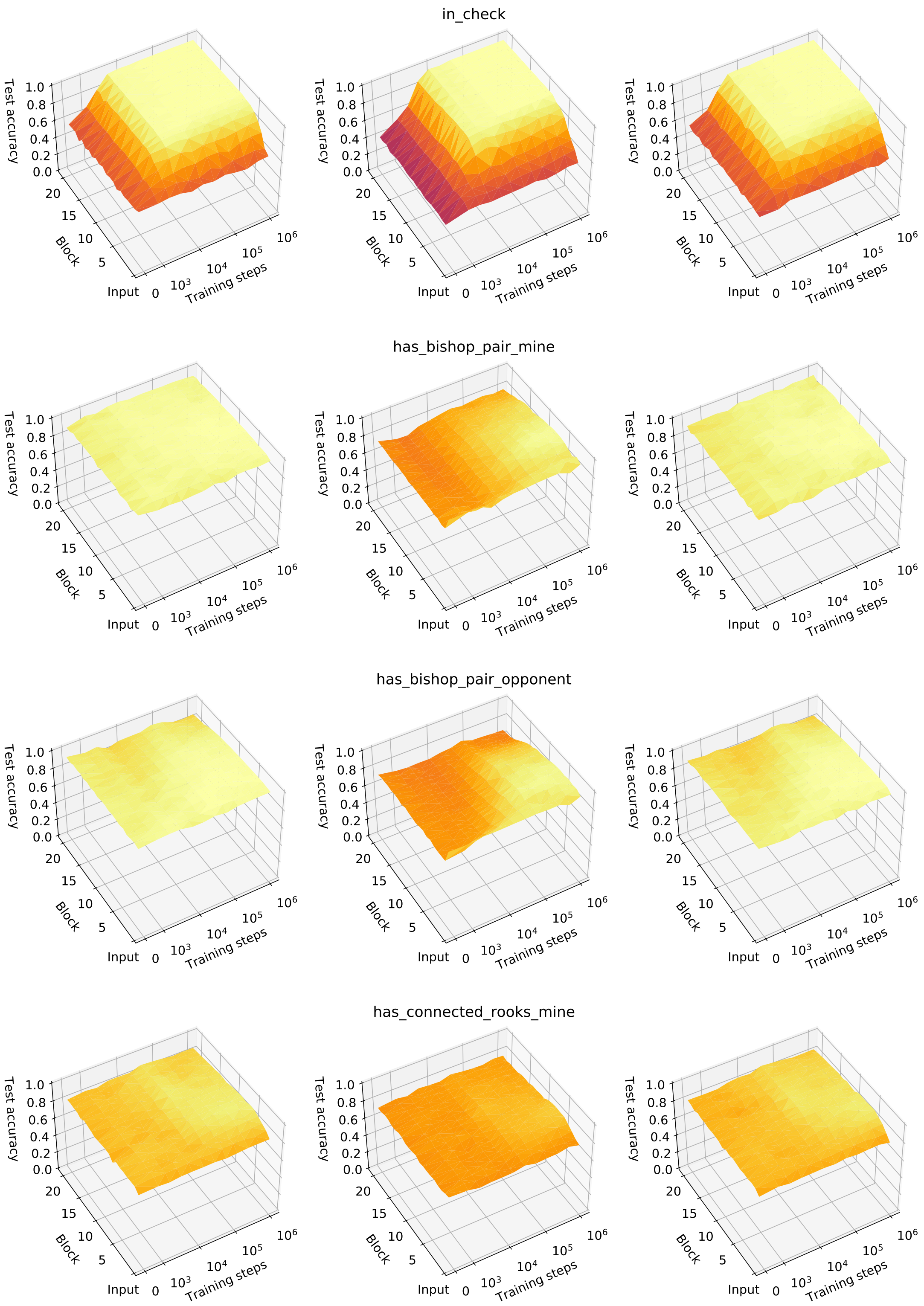

Fig. S68. Comparison of concept regression results between regularisers. Left: unregularised, centre: l_1 regularised, right: group-sparse.

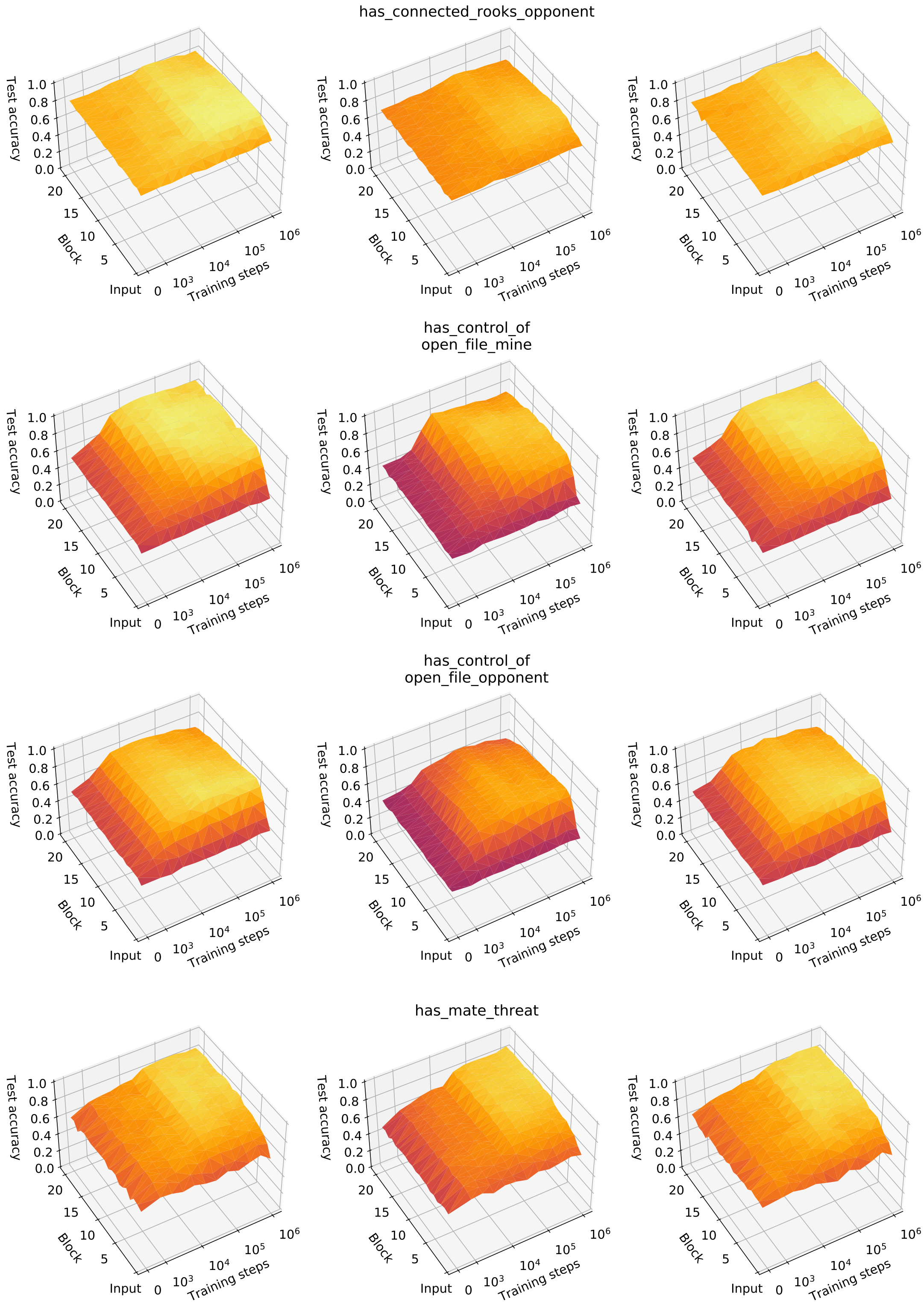

Fig. S69. Comparison of concept regression results between regularisers. Left: unregularised, centre: l_1 regularised, right: group-sparse.

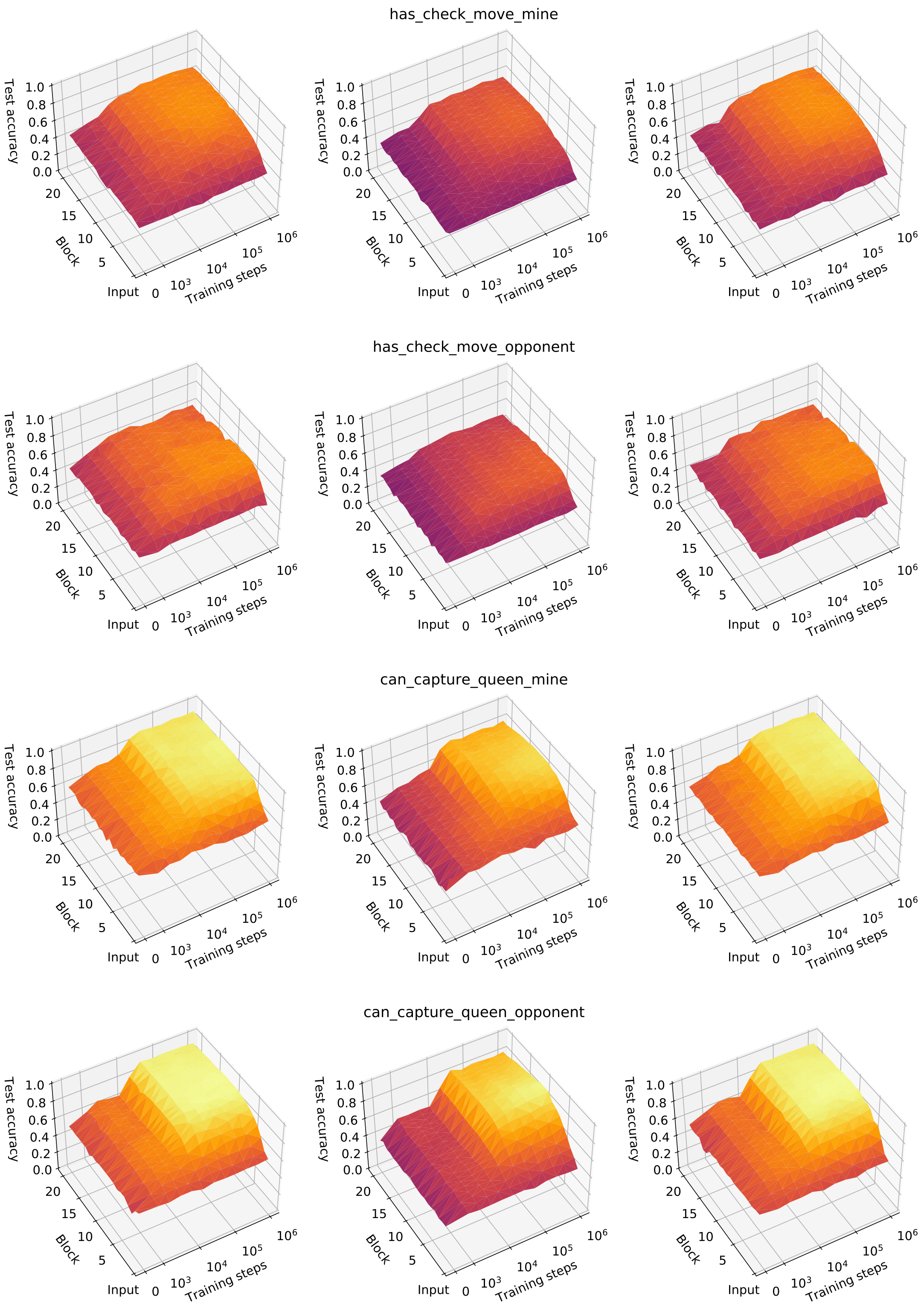

Fig. S70. Comparison of concept regression results between regularisers. Left: unregularised, centre: l_1 regularised, right: group-sparse.

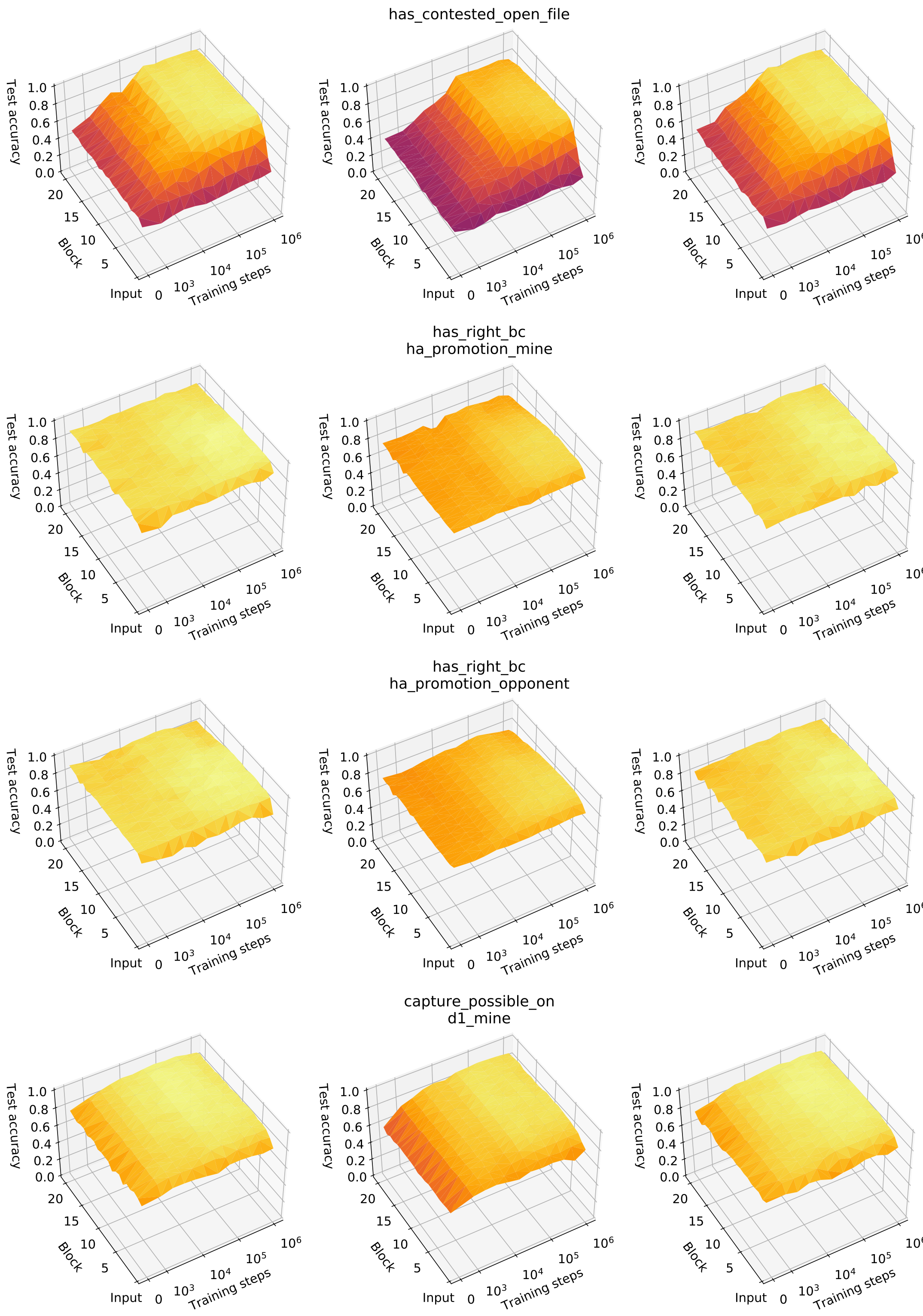

Fig. S71. Comparison of concept regression results between regularisers. Left: unregularised, centre: l_1 regularised, right: group-sparse.

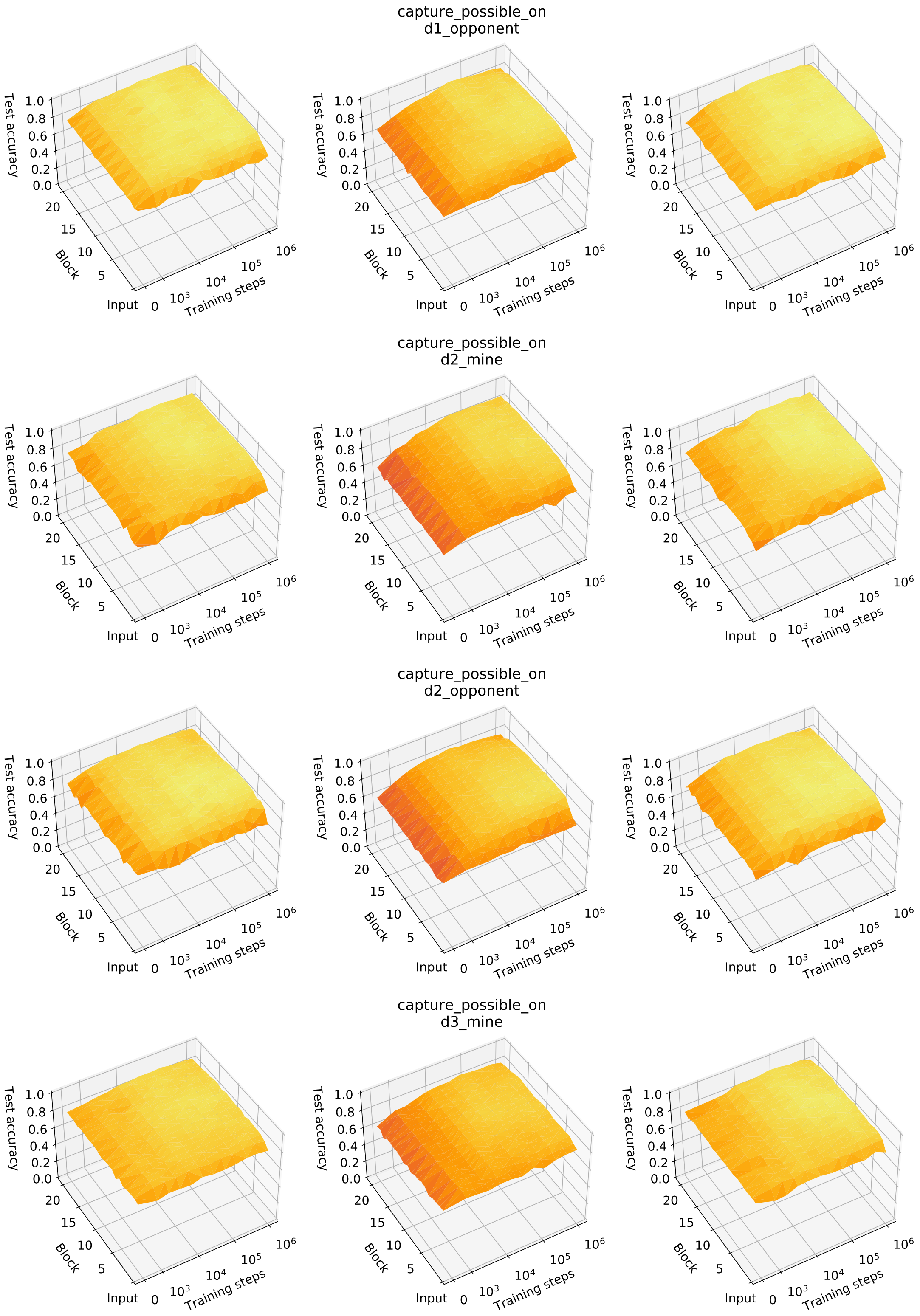

Fig. S72. Comparison of concept regression results between regularisers. Left: unregularised, centre: l_1 regularised, right: group-sparse.

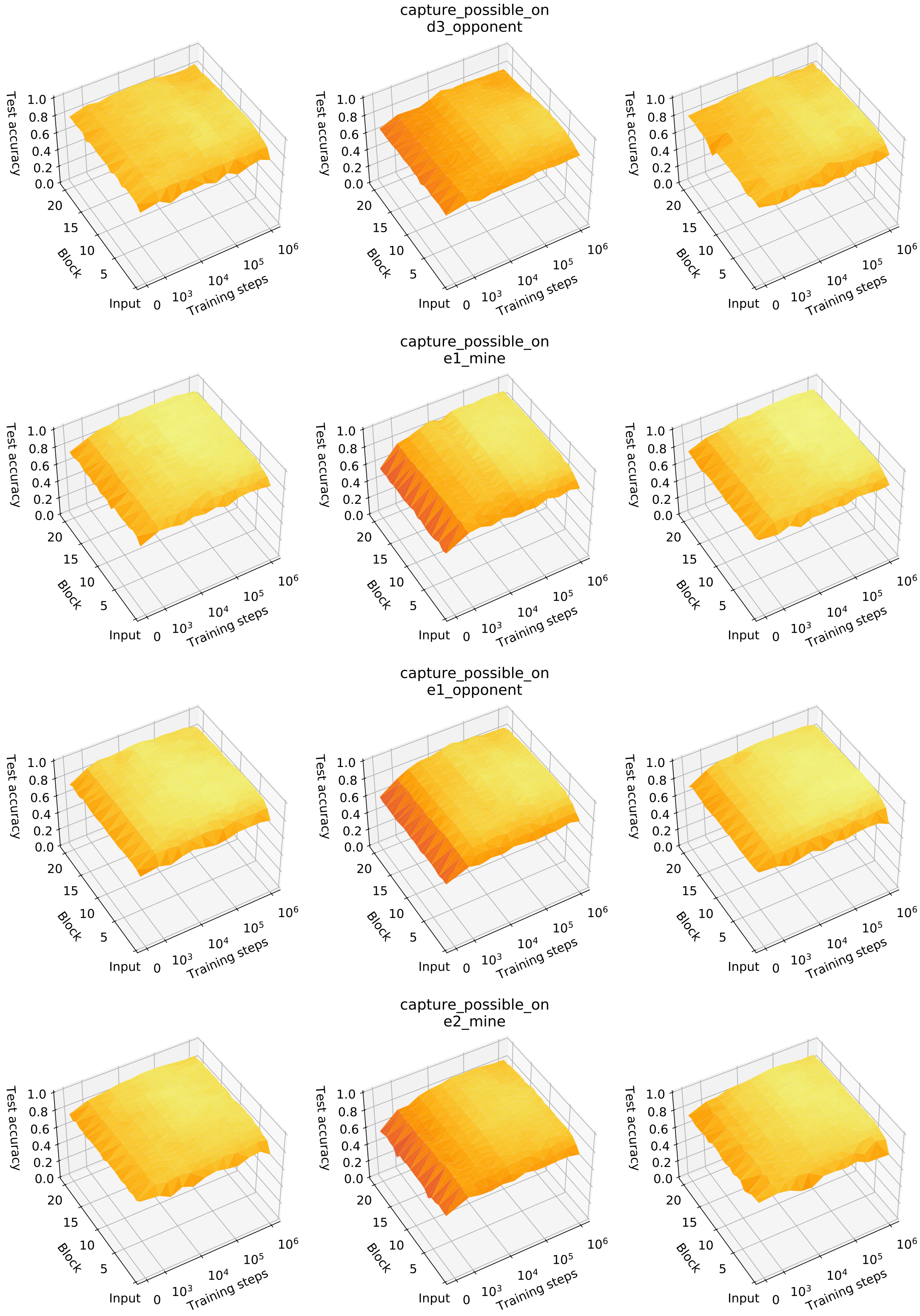

Fig. S73. Comparison of concept regression results between regularisers. Left: unregularised, centre: l_1 regularised, right: group-sparse.

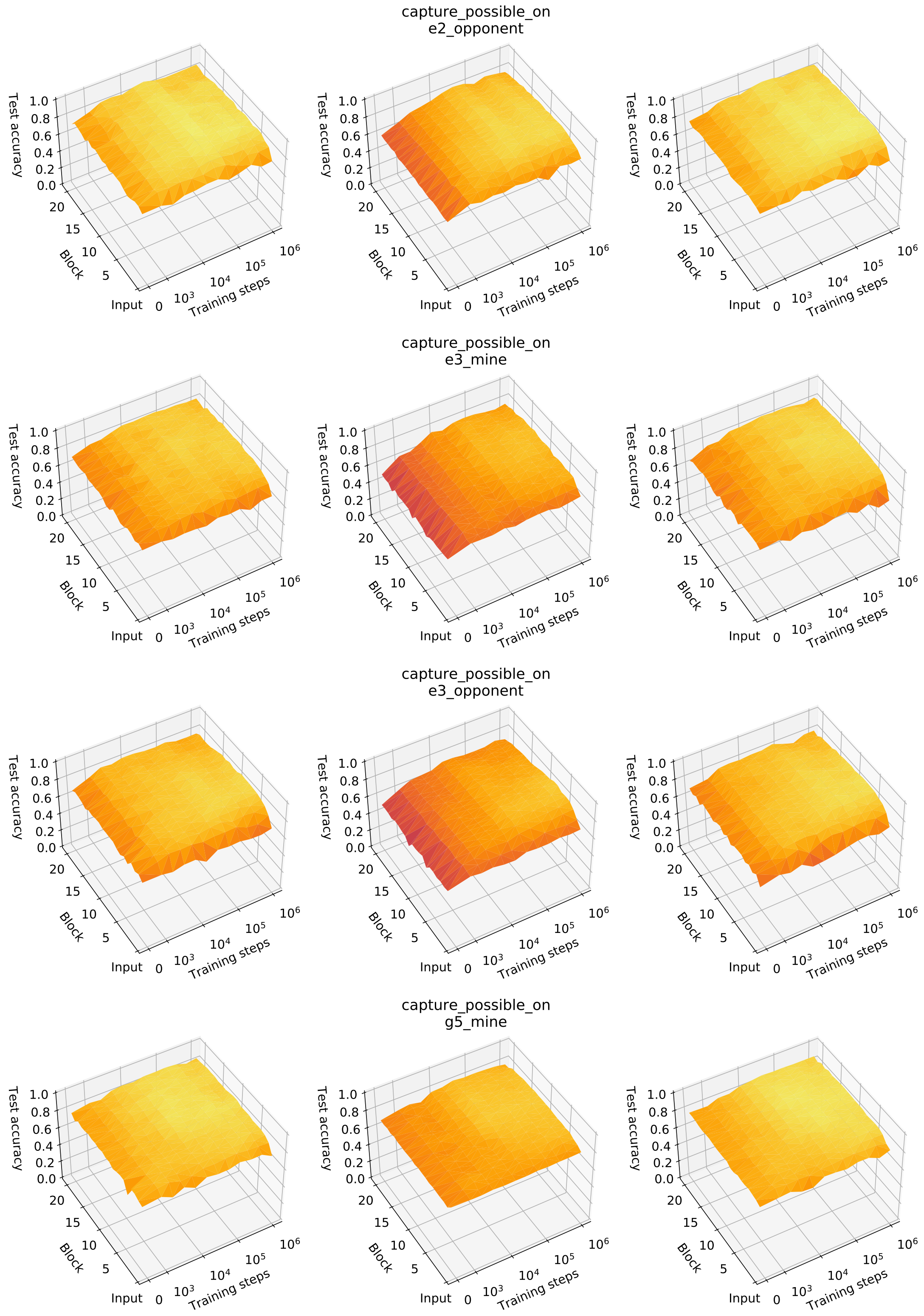

Fig. S74. Comparison of concept regression results between regularisers. Left: unregularised, centre: l_1 regularised, right: group-sparse.

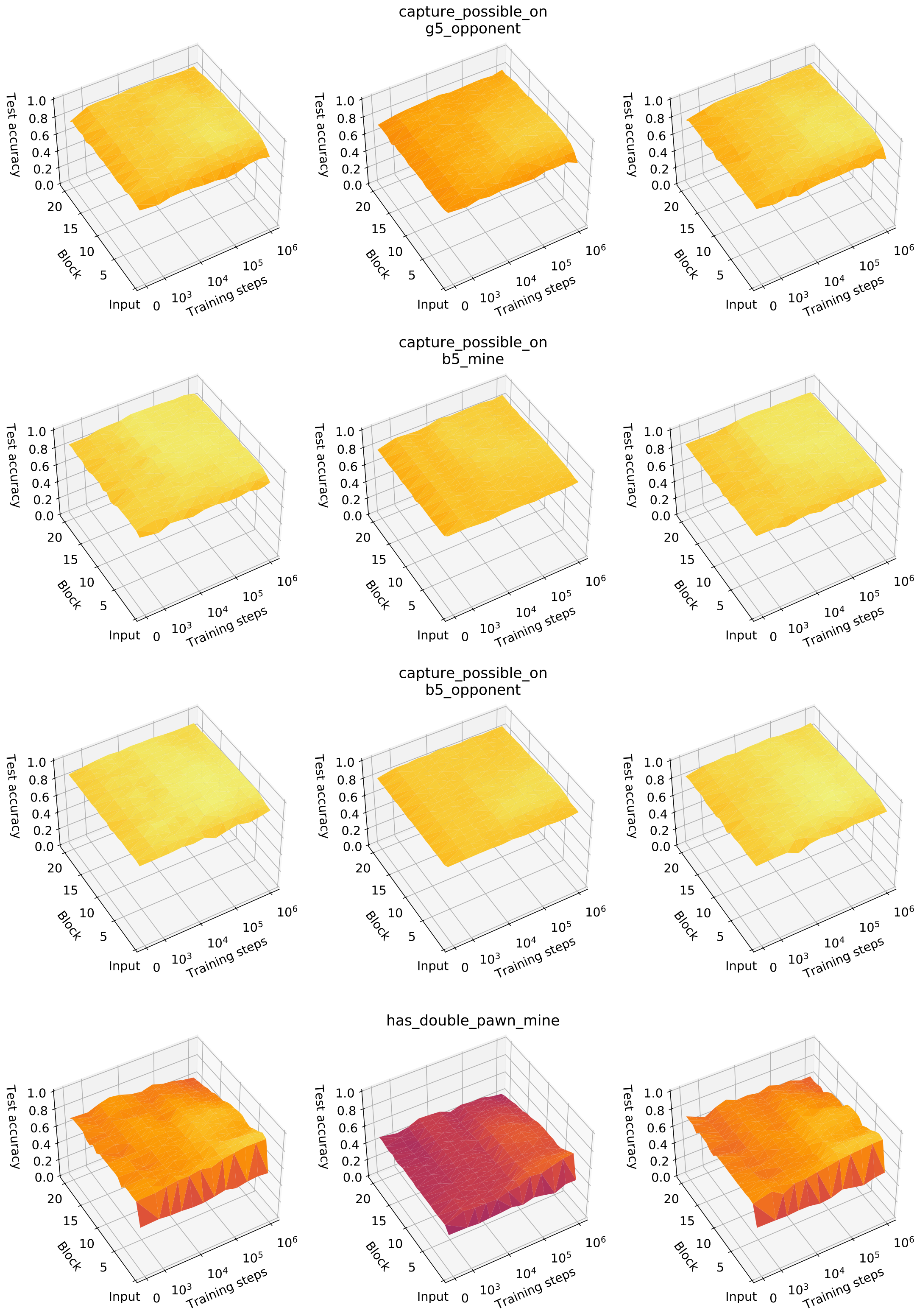

Fig. S75. Comparison of concept regression results between regularisers. Left: unregularised, centre: l_1 regularised, right: group-sparse.

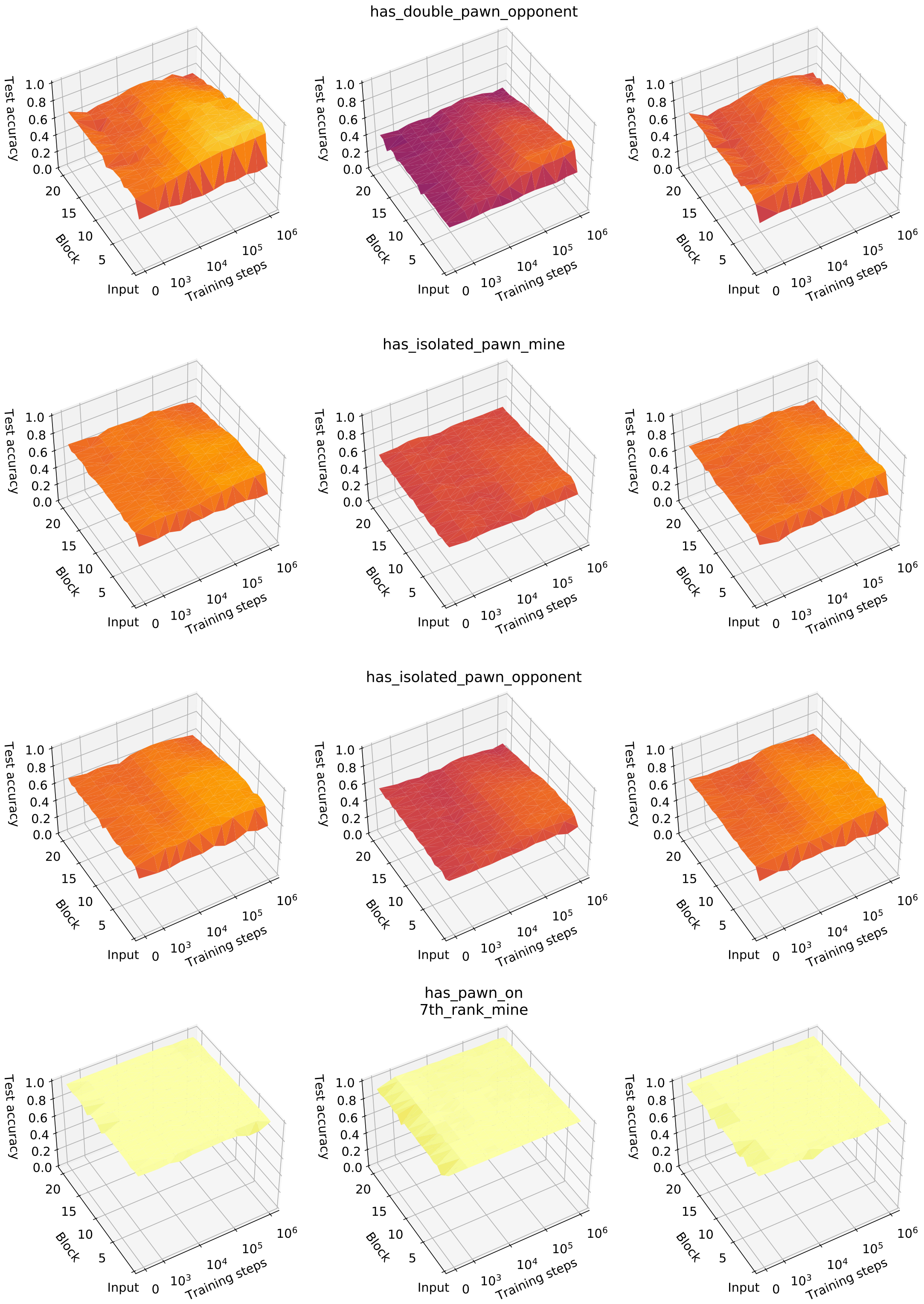

Fig. S76. Comparison of concept regression results between regularisers. Left: unregularised, centre: l_1 regularised, right: group-sparse.

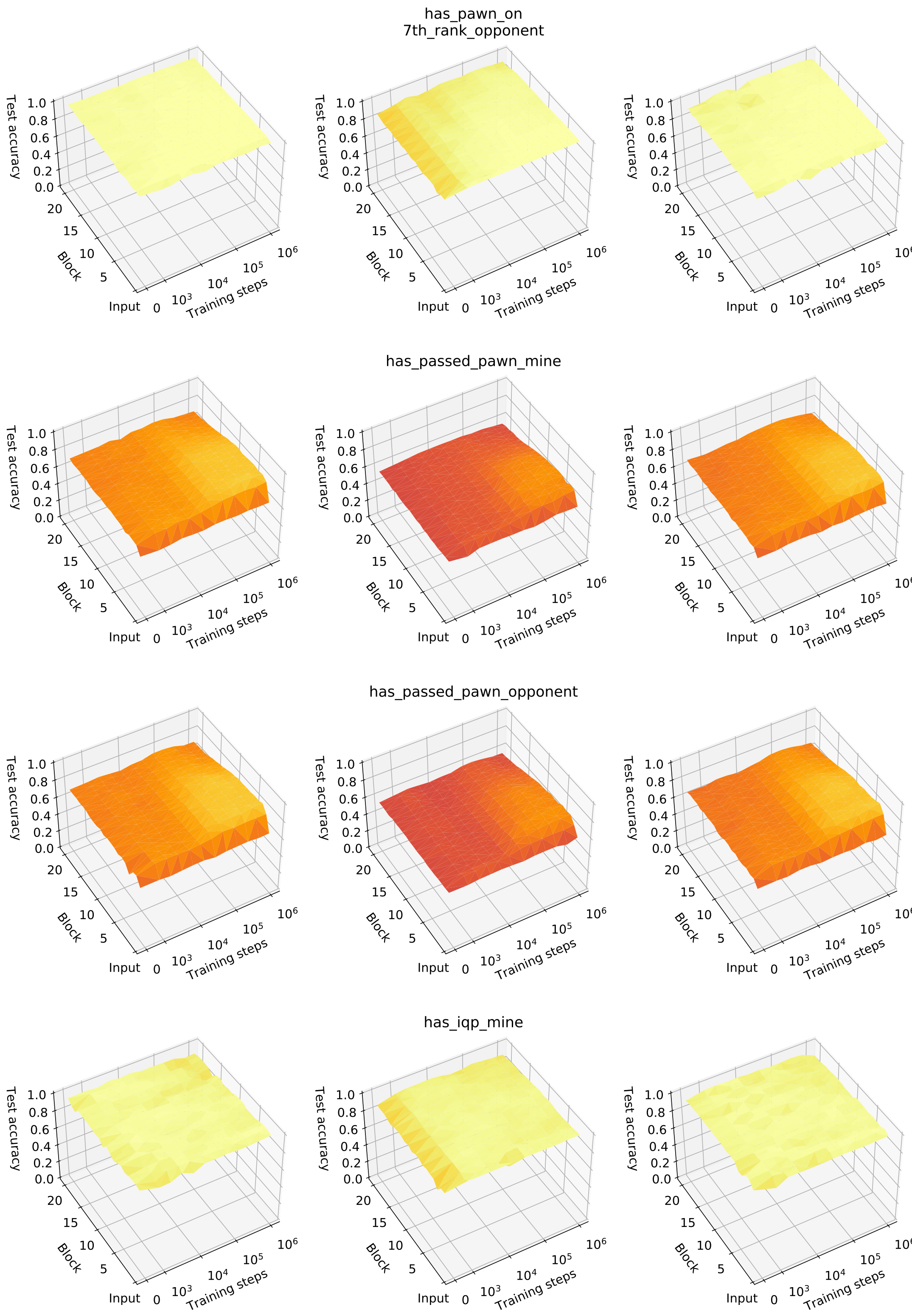

Fig. S77. Comparison of concept regression results between regularisers. Left: unregularised, centre: l_1 regularised, right: group-sparse.

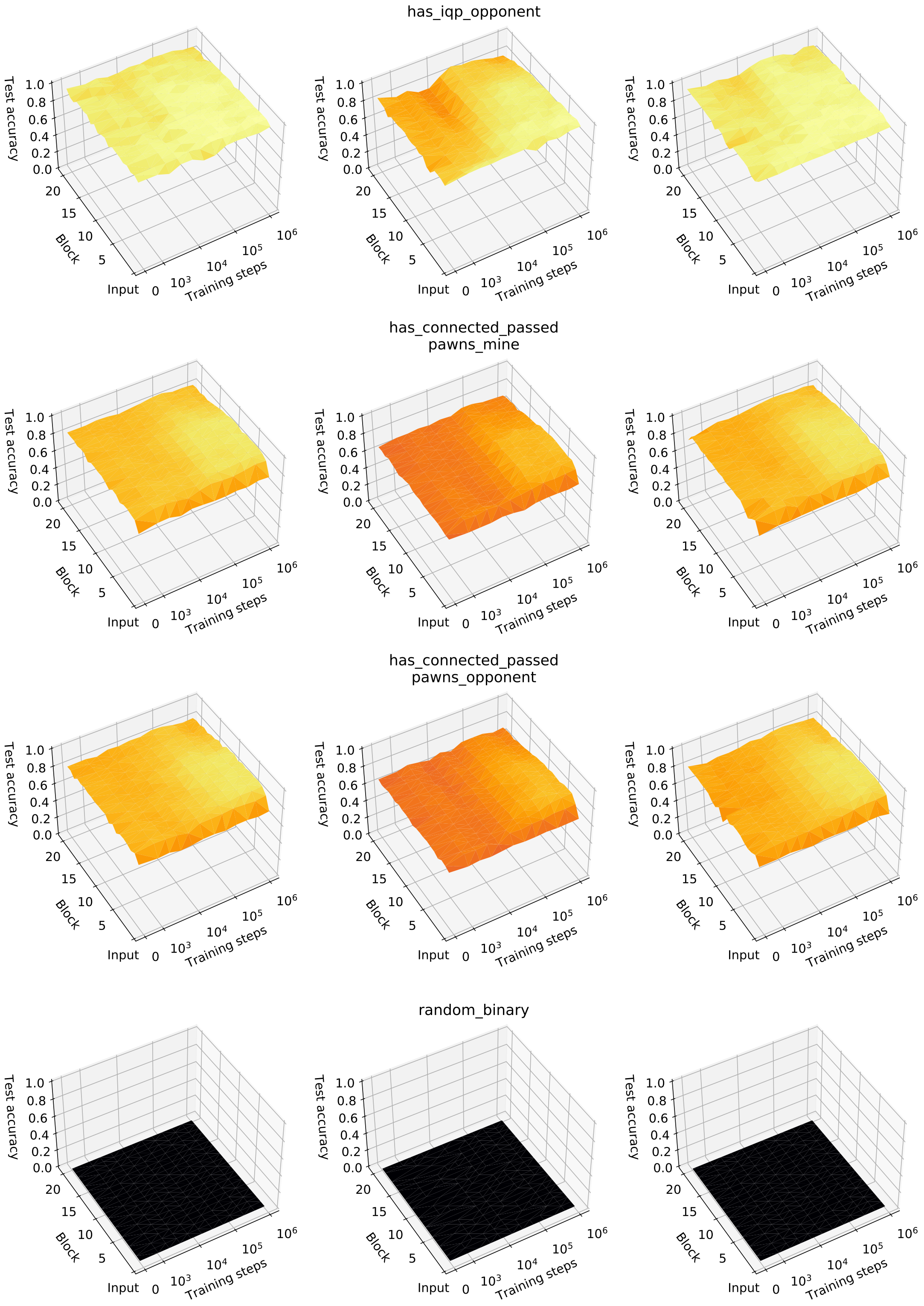

Fig. S78. Comparison of concept regression results between regularisers. Left: unregularised, centre: l_1 regularised, right: group-sparse.

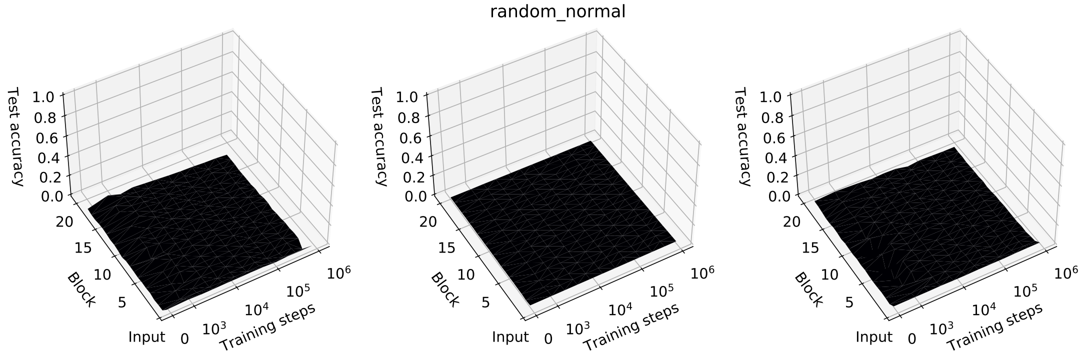

Fig. S79. Comparison of concept regression results between regularisers. Left: unregularised, centre: l_1 regularised, right: group-sparse.

References

1. T McGrath, et al., Acquisition of chess knowledge in AlphaZero. *arXiv preprint arXiv:2111.09259* (2021).
2. D Silver, et al., A general reinforcement learning algorithm that masters chess, shogi, and Go through self-play. *Science* **362**, 1140–1144 (2018).
3. K He, X Zhang, S Ren, J Sun, Deep residual learning for image recognition in *Proceedings of the IEEE Conference on Computer Vision and Pattern Recognition*. pp. 770–778 (2016).
4. CD Rosin, Multi-armed bandits with episode context. *Annals Math. Artif. Intell.* **61**, 203–230 (2011).
5. M Sadler, N Regan, *Game Changer: AlphaZero's Groundbreaking Chess Strategies and the Promise of AI.* (New In Chess), (2019).
6. DD Lee, HS Seung, Learning the parts of objects by non-negative matrix factorization. *Nature* **401**, 788–791 (1999).
7. C Olah, et al., The building blocks of interpretability. *Distill* **3**, e10 (2018).
8. J Hilton, N Cammarata, S Carter, G Goh, C Olah, Understanding rl vision. *Distill* **5**, e29 (2020).
9. ML Leavitt, A Morcos, Towards falsifiable interpretability research. *arXiv preprint arXiv:2010.12016* (2020).
